# Supplementary material for: Recrudescence of transmission of onchocerciasis in some endemic communities in Kaduna State, Nigeria: What is the way forward?
Source: PLoS Negl Trop Dis. 2025 Aug 7;19(8):e0012495. doi: 10.1371/journal.pntd.0012495 (PMC12331076; doi:10.1371/journal.pntd.0012495)
Supplement: S1 Methods — (S1_Methods.DOCX) [file pntd.0012495.s001.docx]

**Protocol for Ov16 ELISA**

The following steps were followed to carry out Ov16 enzyme-linked immunosorbent assay (ELISA) of dried blood samples (DBS) collected from children aged 5-9 years across various communities/schools in Kaduna State; this can be adopted for repeatability or other studies that required Ov16 ELISA

| 1. **Create a map:** Use the map below. The samples are run in duplicate as indicated.  \|  \| 1 \| 2 \| 3 \| 4 \| 5 \| 6 \| 7 \| 8 \| 9 \| 10 \| 11 \| 12 \| \| --- \| --- \| --- \| --- \| --- \| --- \| --- \| --- \| --- \| --- \| --- \| --- \| --- \| \| A \| STD 640 \| STD 640 \| Blank \| Blank \| S7 \| S7 \| S15 \| S15 \| S23 \| S23 \| S31 \| S31 \| \| B \| STD 320 \| STD 320 \| Blank \| Blank \| S8 \| S8 \| S16 \| S16 \| S24 \| S24 \| S32 \| S32 \| \| C \| STD 160 \| STD 160 \| S1 \| S3 \| S9 \| S9 \| S17 \| S17 \| S25 \| S25 \| S33 \| S33 \| \| D \| STD 80 \| STD 80 \| S1 \| S3 \| S10 \| S10 \| S18 \| S18 \| S26 \| S26 \| S34 \| S34 \| \| E \| STD 40 \| STD 40 \| S2 \| S4 \| S11 \| S11 \| S19 \| S19 \| S27 \| S27 \| S35 \| S35 \| \| F \| STD 20 \| STD 20 \| S2 \| S4 \| S12 \| S12 \| S20 \| S20 \| S28 \| S28 \| S36 \| S36 \| \| G \| STD 10 \| STD 10 \| S5 \| S5 \| S13 \| S13 \| S21 \| S21 \| S29 \| S29 \| S37 \| S37 \| \| H \| STD 5 \| STD 5 \| S6 \| S6 \| S14 \| S14 \| S22 \| S22 \| S30 \| S30 \| S38 \| S38 \| | |
| --- | --- | --- | --- | --- | --- | --- | --- | --- | --- | --- | --- | --- | --- | --- | --- | --- | --- | --- | --- | --- | --- | --- | --- | --- | --- | --- | --- | --- | --- | --- | --- | --- | --- | --- | --- | --- | --- | --- | --- | --- | --- | --- | --- | --- | --- | --- | --- | --- | --- | --- | --- | --- | --- | --- | --- | --- | --- | --- | --- | --- | --- | --- | --- | --- | --- | --- | --- | --- | --- | --- | --- | --- | --- | --- | --- | --- | --- | --- | --- | --- | --- | --- | --- | --- | --- | --- | --- | --- | --- | --- | --- | --- | --- | --- | --- | --- | --- | --- | --- | --- | --- | --- | --- | --- | --- | --- | --- | --- | --- | --- | --- | --- | --- | --- | --- | --- | --- | --- |
| STD = positive control dilutions (STD 640 = 50ul of 250ng/mL)  S = sample wells  **2. Elution from filter paper samples**: Punch out duplicate spots from the dried blood samples collected on the filter papers using a standard 6mm paper punch. Using your map, place the duplicate punches of the blood spots into the sample wells. Add 200 μL of PBST-BSA to each sample. Push the punches to the bottom of the well and then mix 10 times by pipetting. Cover the plates with a plate sealer and incubate them at 4°C overnight. Store the eluted serum samples at -20^o^C. |  |
| **3. Coating plate with antigen**: Dilute Ov16-GST antigen to 2.0 μg/mL in coating buffer. Add 100 μL to each well. Place the plate in a ziplock bag and incubate it overnight at 4°C. |  |
|  |  |
| **4.** **Washing plates**: Wash 4 times with PBST, using a wash bottle. Do not dry between washes. But dry the plate after last wash.  **5. Blocking plates:** Add 100 μL of PBST-BSA, place the plate in a ziplock bag (in the absence of plate sealer) and incubate it at 4°C for 1 hour.  **6. Preparation of standard curve and blank:** During the incubation of step 5 prepare 250ul of a 250ng/mL of the McAb in antibody dilution buffer. This is equivalent to 640 units (per 50ul) of the old pooled serum positive control. Prepare a series of seven two-fold dilutions of the McAb. Start with 125ul of the 250 ng/mL stock, and add it to 125ul of antibody dilution buffer. This will be a solution of 125 ng/mL or 320 units/50ul. Continue making the additional dilutions until you have a total of 8 dilutions in the series, which will range from 640 units/50ul to 5 units/50ul. Set aside 250ul of antibody dilution buffer to use as blanks. |  |
| **7. Empty plate**: After the incubation of step 5, empty the PBST-BSA in the sink and dry the plate. Do not wash.  **8. Adding samples**: Use your map, add 50μL of each serum sample (positive control dilution, blanks and eluted samples) to the corresponding wells on the plate map. Place the plate in a ziplock bag and incubate it at room temperature for 2 hours. |  |
| **9. Washing plates**: Wash 4 times with PBST, using a wash bottle. Dry the plate after the first wash, then carry out the remaining three washes without drying the plate between the wash steps. Dry the plate after the 4^th^ wash.  **10. Add conjugate**: 10 minutes before the time is up for the last incubation, prepare the conjugate. Dilute the anti-human IgG_4_ antibody conjugated to biotin 1:1000 in PBST. Add 50μL of the diluted conjugate to all wells. Place the plate in a ziplock bag and incubate it at room temperature for 1 hour. |  |
| **11. Washing plates:** Wash 4 times with PBST, using a wash bottle. Do not dry between washes. But dry the plate after last wash.  **12. Add Streptavidin-AP**: 10 minutes before the time is up for the last incubation, prepare the streptavidin-AP. Dilute streptavidin-AP 1:2000 in PBST. Add 50μL to all wells cover the plate and incubate it at room temperature for 1 hour.  **13. Washing plates:** Wash 4 times with PBST, using a wash bottle. Do not dry between washes. But dry the plate after last wash.  **WE USED BIOBASE-EL10A ELISA READER.** |  |
| **14. Substrate:** 15 min before the incubation period is over switch on the ELISA plate reader. Prepare PNPP solution with the substrate buffer by dissolving 1 tablet in 5mL of the buffer provided. Add 50μL to each well. If your reader reads the whole plate at once, then read the plate until the 1/20 standard is around OD 1.5. If your reader reads well by well, then read only the two wells of the 1/20 standard every minute, and shake the plate between readings. When they are around 0.9 OD, start reading more frequently. When you reach OD 1.1, stop the reaction by adding 25μL of 3M NaOH to each well. Shake the plates to stop the reaction. Incubate the plate for 5 minutes at room temperature and read it at 405nm. |  |
