# Supplementary material for: Recrudescence of transmission of onchocerciasis in some endemic communities in Kaduna State, Nigeria: What is the way forward?
Source: PLoS Negl Trop Dis. 2025 Aug 7;19(8):e0012495. doi: 10.1371/journal.pntd.0012495 (PMC12331076; doi:10.1371/journal.pntd.0012495)
Supplement: S2 Methods — (S2_Methods.DOCX) [file pntd.0012495.s002.docx]

**Detailed protocol for DNA isolation from black fly heads and RT-qPCR**

The following steps were followed to carry out DNA extraction and Real-Time Polymerase Chain Reaction (RT-qPCR)

1. **Extraction and purification of DNA using QIAGEN (Adopted from Hendy et al., 2017)**

QIAGEN Dneasy Blood & Tissue Kits (Qiagen, N.V.)

**Notes before starting:**

- Perform all centrifugation steps at RT
- Re-dissolve any precipitations in Buffer AL and Buffer ATL
- Add ethanol to Buffer AW1 & AW2 concentrates
- Equilibrate frozen tissue or cell plates to RT
- Preheat an incubator to 56℃

1a. For Tissue sample: Cut tissue (< 10mg spleen or <25mg other tissue) into small pieces, and place in a 1.5mL microcentrifuge tube. Add 180µL Buffer ATL, 20µL Proteinase K, mix by vortexing and incubate at 56℃ until completely lysed. Vortex at intervals during incubation. Vortex 15 secs directly before proceeding to step 2

1b. Nonnucleated blood: pipette 20µL Proteinase K into 1.5mL or 2mL microcentrifuge tube. Add 50-100µL anticoagulated blood. Adjust volume to 220µL with PBS. Proceed to step 2

2. Add 200µL of Buffer AL. Mix thoroughly by vortexing. Incubate blood samples at 56℃ for 10mins

3. Add 200µL ethanol (96-100%). Mix thoroughly by vortexing.

4. Pipette the mixture into a DNeasy Mini spin column placed in a 2mL collection tube. Centrifuge at >6000g (8000rpm) for 1min. Discard the flow through and the collection tube.

5. Place the spin column into a new 2mL collection tube. Add 500µL Buffer AW1. Centrifuge for 1min at >6000g (8000rpm). Discard the flow through and the collection tube.

6. Place the spin column into a new 2mL collection tube. Add 500µL Buffer AW2. Centrifuge for 1min at >20000g (14000rpm). Discard the flow through and the collection tube.

7. Transfer the spin column to a new 1.5mL or 2mL microcentrifuge tube.

8. Elute the DNA by adding 200µL Buffer AE to the center of the spin column membrane. Incubate for 1min at RT. Centrifuge for 1min at >6000g

9. Optional: Repeat step 8 for increased DNA yield.

1. **Real-Time Polymerase Chain Reaction (RT-qPCR) procedure for molecular detection of O. *vulvulus* and *O. ochengi* (OV ND5 qPCR) (Adopted from Hendy et al., 2017)**

**RT-qPCR Master Mix**

| **Constituent** | **Volume (µL)** |
| --- | --- |
| Template DNA | 2 |
| 5X FirePol HotTaq | 4 |
| PCR Water | 5 |
| OvOo ND5 forward | 2 |
| OvOo ND5 reverse | 2 |
| Ov probe | 2 |
| Oo probe | 2 |
| 16S rDNA forward | 2 |
| 16S rDNA reverse | 2 |
| 16S rDNA probe | 2 |
| Total Volume | 25µL |

**RT-qPCR Temperature Conditions for ND5 qPCR Pool Screening analysis (version 2.1)**

|  | **Cycle** | **Temperature** | **Time** |
| --- | --- | --- | --- |
| Segment 1 | 1cycle | 95℃ | 15mins |
| Segment 2 | 45cycles | 95℃ | 30 secs |
|  |  | 58℃ | 45 secs |

**Florescence Setting (CFX96)**

| Target | Flourophore |
| --- | --- |
| (O. volvulus) Ov | FAM |
| (O. ochengi) Oo | HEX |
| 16rDNA | Cy5 |
|  |  |

1. **Analysis Setting (CFX96)**

**Ct-Value samples less than 40 for a particular gene target will be selected as positive. All the Internal Control must show amplification.**
