# Supplementary material for: Recrudescence of transmission of onchocerciasis in some endemic communities in Kaduna State, Nigeria: What is the way forward?
Source: PLoS Negl Trop Dis. 2025 Aug 7;19(8):e0012495. doi: 10.1371/journal.pntd.0012495 (PMC12331076; doi:10.1371/journal.pntd.0012495)
Supplement: S3 Methods — (S3_Methods.DOCX) [file pntd.0012495.s003.docx]

**Statistical variation in fly population density**

| **LGA** | **Community/Catching Site** | **Number of fly caught** | **Number of black fly caught** |
| --- | --- | --- | --- |
| A | KAB | 148 | 2 |
| B | KIN | 27 | 2 |
| C | BBS | 11 | 0 |
| D | ATA | 2233 | 2072 |
| E | KUT | 2924 | 2858 |
| F | AMU | 1175 | 782 |
| G | GUR | 1264 | 1181 |

**Assessing the Variation in the number of flies caught across the catching sites Kaduna state, Nigeria**

The null hypothesis (H₀) for assessing variation in the number of flies caught at different sites is:

**H₀: There is no significant difference in the number of flies caught across the different sites.**

|  | Observed N | Expected N | Residual |
| --- | --- | --- | --- |
| KAB | 148 | 1111.7 | -963.7 |
| KIN | 27 | 1111.7 | -1084.7 |
| BBS | 11 | 1111.7 | -1100.7 |
| ATA | 2233 | 1111.7 | 1121.3 |
| KUT | 2924 | 1111.7 | 1812.3 |
| AMU | 1175 | 1111.7 | 63.3 |
| GUR | 1264 | 1111.7 | 152.3 |
| Total | 7782 |  |  |

The table presents data for the number of flies caught at different catching sites, comparing **observed counts** with **expected counts** and calculating the **residuals** (difference between observed and expected counts). Further explanation of the results is as follows:

**Key Observations:**

1. **Expected N**: The expected number (1111.7) is the same for all sites. This likely assumes flies are evenly distributed among the sites under the null hypothesis.
2. **Residual**: The residual is the difference between the observed and expected counts.

**Site-Specific Observations:**

- **KAB, KIN, BBS**: These sites have **observed counts much lower than expected** (large negative residuals), indicating fewer flies were caught here than anticipated.
- **ATA, KUT**: These sites have **observed counts significantly higher than expected** (large positive residuals), showing more flies were caught here than predicted.
- **AMU, GUR**: The observed counts are relatively close to the expected counts (small residuals), suggesting fly catches in these sites are approximately as expected.

The **total observed N** is 7782, consistent with the total across all sites. The large deviations from the expected counts across individual sites suggest uneven distribution of flies across the sites. The large residuals indicate that there is a significant variation in fly catches across the sites. This violates the assumption of equal distribution under the null hypothesis and suggests that the fly population is not uniformly distributed across the catching sites.

| **Test Statistics** | |
| --- | --- |
|  | Catching Site |
| Chi-Square | 7093.351^a^ |
| df | 6 |
| Asymp. Sig. | .0001 |

The provided test statistics indicate the results of a Chi-square goodness-of-fit test to evaluate whether the observed number of flies caught at different sites deviates significantly from the expected uniform distribution. Since p<0.05, we **reject the null hypothesis**. This means that there is a statistically significant variation in the number of flies caught across the different sites. Thus, it can be concluded that the number of flies caught varies significantly among the catching sites. This could be due to environmental factors, site-specific conditions, or other factors influencing fly distribution. Further investigation would be needed to understand the underlying causes of this variation.

**Assessing the Variation in the number of Black flies caught across the catching sites in Kaduna State, Nigeria**

The null hypothesis (H₀) for assessing variation in the number of black flies caught at different sites is:

**H₀: There is no significant difference in the number of black flies caught across the different sites.**

|  | Observed N | Expected N | Residual |
| --- | --- | --- | --- |
| KAB | 2 | 1149.5 | -1147.5 |
| KIN | 2 | 1149.5 | -1147.5 |
| ATA | 2072 | 1149.5 | 922.5 |
| KUT | 2858 | 1149.5 | 1708.5 |
| AMU | 782 | 1149.5 | -367.5 |
| GUR | 1181 | 1149.5 | 31.5 |
| Total | 6897 |  |  |

The table shows the observed and expected counts of black flies caught at different sites, along with the residuals (differences between observed and expected values).

**Key Observations:**

1. **Expected N**: The expected number (1149.5) assumed uniform distribution of black flies across the sites, calculated as the total number of black flies (6897) divided equally among the six sites.
2. **Residual**: These values indicate how much each site deviates from the expected number of flies. Positive residuals mean more flies were caught than expected, while negative residuals mean fewer were caught.

**Site-Specific Observations:**

**KAB and KIN**: These sites had significantly fewer flies than expected, with residuals indicating an extreme shortfall. These sites had almost no black flies caught, indicating they may not be suitable habitats or lack conditions favorable for black fly populations.

**ATA and KUT**: These sites caught substantially more black flies than expected, showing that they are hotspots for fly activity. These sites are major black fly hotspots, with significantly higher catches than expected, suggesting favorable conditions or high infestation levels.

**AMU**: This site caught fewer flies than expected but is closer to the expected value compared to KAB and KIN. These sites have distributions closer to uniformity, indicating more moderate activity levels.

**GUR**: The observed count is very close to the expected count, suggesting minimal deviation at this site. These sites have distributions closer to uniformity, indicating more moderate activity levels.

The **total observed N** is 6897, consistent with the total across all sites. The large deviations from the expected counts across individual sites suggest uneven distribution of flies across the sites.

| **Test Statistics** | |
| --- | --- |
|  | Catching Site |
| Chi-Square | 5689.030^a^ |
| df | 5 |
| Asymp. Sig. | .0001 |

The provided test statistics indicate the results of a Chi-square goodness-of-fit test to evaluate whether the observed number of black flies caught at different sites deviates significantly from the expected uniform distribution. Since p<0.05, we **reject the null hypothesis**. This means that there is a statistically significant variation in the number of black flies caught across the different sites. The data strongly suggest that the number of black flies varies significantly among the catching sites, with some sites showing extremely high or low activity. This uneven distribution could be influenced by environmental factors, breeding sites, or other ecological conditions.

NULL HYPOTHESIS

**H₀: There is no significant variation in the number of flies caught across different catching sites between the different periods of collection.**

| DESCRIPTIVE STATISTICS FOR THE NUMBER OF FLIES CAUGHT AT DIFFERENT SITES BETWEEN FOUR MONTHS | | | | | | | | |
| --- | --- | --- | --- | --- | --- | --- | --- | --- |
|  | N | Mean | Std. Deviation | Std. Error | 95% Confidence Interval for Mean | | Minimum | Maximum |
|  |  |  |  |  | Lower Bound | Upper Bound |  |  |
| KAB | 4 | 37.0000 | 8.24621 | 4.12311 | 23.8784 | 50.1216 | 30.00 | 46.00 |
| KIN | 4 | 6.7500 | 3.86221 | 1.93111 | .6044 | 12.8956 | 3.00 | 12.00 |
| BBS | 4 | 2.7500 | 1.89297 | .94648 | -.2621 | 5.7621 | .00 | 4.00 |
| ATA | 4 | 558.2500 | 148.30459 | 74.15229 | 322.2643 | 794.2357 | 344.00 | 683.00 |
| KUT | 4 | 731.0000 | 70.77193 | 35.38597 | 618.3861 | 843.6139 | 650.00 | 813.00 |
| AMU | 4 | 293.7500 | 113.71712 | 56.85856 | 112.8007 | 474.6993 | 130.00 | 387.00 |
| GUR | 4 | 316.0000 | 16.71327 | 8.35663 | 289.4055 | 342.5945 | 293.00 | 333.00 |
| Total | 28 | 277.9286 | 278.37796 | 52.60849 | 169.9849 | 385.8723 | .00 | 813.00 |

The above results show the descriptive statistics for the number of flies caught at different catching sites for the months under study.

| **ANOVA** | | | | | |
| --- | --- | --- | --- | --- | --- |
|  | Sum of Squares | df | Mean Square | F | Sig. |
| Between Groups | 1971444.857 | 6 | 328574.143 | 57.072 | .0001 |
| Within Groups | 120901.000 | 21 | 5757.190 |  |  |
| Total | 2092345.857 | 27 |  |  |  |

The p-value (p<0.05) is extremely small, indicating that the observed differences are statistically significant.

The null hypothesis states that there is no significant variation in the number of flies caught across catching sites in relation to the period of collection.

The analysis shows a statistically significant F-statistic with p=0.0001p = 0.0001p=0.0001, far below the typical threshold (p<0.05) for significance.We reject the null hypothesis and conclude that there is strong evidence to suggest that there is significant variation in the number of flies caught across different catching sites and collection periods.

**Post Hoc Tests (One-way ANOVA; SPSS 23.0)**

| **Multiple Comparisons** | | | | | | |
| --- | --- | --- | --- | --- | --- | --- |
| Dependent Variable: Number of Fly | | | | | | |
| LSD | | | | | | |
| (I) site | (J) site | Mean Difference (I-J) | Std. Error | Sig. | 95% Confidence Interval | |
|  |  |  |  |  | Lower Bound | Upper Bound |
| KAB | KIN | 30.25000 | 53.65254 | .579 | -81.3266 | 141.8266 |
|  | BBS | 34.25000 | 53.65254 | .530 | -77.3266 | 145.8266 |
|  | ATA | -521.25000^*^ | 53.65254 | .000 | -632.8266 | -409.6734 |
|  | KUT | -694.00000^*^ | 53.65254 | .000 | -805.5766 | -582.4234 |
|  | AMU | -256.75000^*^ | 53.65254 | .000 | -368.3266 | -145.1734 |
|  | GUR | -279.00000^*^ | 53.65254 | .000 | -390.5766 | -167.4234 |
| KIN | KAB | -30.25000 | 53.65254 | .579 | -141.8266 | 81.3266 |
|  | BBS | 4.00000 | 53.65254 | .941 | -107.5766 | 115.5766 |
|  | ATA | -551.50000^*^ | 53.65254 | .000 | -663.0766 | -439.9234 |
|  | KUT | -724.25000^*^ | 53.65254 | .000 | -835.8266 | -612.6734 |
|  | AMU | -287.00000^*^ | 53.65254 | .000 | -398.5766 | -175.4234 |
|  | GUR | -309.25000^*^ | 53.65254 | .000 | -420.8266 | -197.6734 |
| BBS | KAB | -34.25000 | 53.65254 | .530 | -145.8266 | 77.3266 |
|  | KIN | -4.00000 | 53.65254 | .941 | -115.5766 | 107.5766 |
|  | ATA | -555.50000^*^ | 53.65254 | .000 | -667.0766 | -443.9234 |
|  | KUT | -728.25000^*^ | 53.65254 | .000 | -839.8266 | -616.6734 |
|  | AMU | -291.00000^*^ | 53.65254 | .000 | -402.5766 | -179.4234 |
|  | GUR | -313.25000^*^ | 53.65254 | .000 | -424.8266 | -201.6734 |
| ATA | KAB | 521.25000^*^ | 53.65254 | .000 | 409.6734 | 632.8266 |
|  | KIN | 551.50000^*^ | 53.65254 | .000 | 439.9234 | 663.0766 |
|  | BBS | 555.50000^*^ | 53.65254 | .000 | 443.9234 | 667.0766 |
|  | KUT | -172.75000^*^ | 53.65254 | .004 | -284.3266 | -61.1734 |
|  | AMU | 264.50000^*^ | 53.65254 | .000 | 152.9234 | 376.0766 |
|  | GUR | 242.25000^*^ | 53.65254 | .000 | 130.6734 | 353.8266 |
| KUT | KAB | 694.00000^*^ | 53.65254 | .000 | 582.4234 | 805.5766 |
|  | KIN | 724.25000^*^ | 53.65254 | .000 | 612.6734 | 835.8266 |
|  | BBS | 728.25000^*^ | 53.65254 | .000 | 616.6734 | 839.8266 |
|  | ATA | 172.75000^*^ | 53.65254 | .004 | 61.1734 | 284.3266 |
|  | AMU | 437.25000^*^ | 53.65254 | .000 | 325.6734 | 548.8266 |
|  | GUR | 415.00000^*^ | 53.65254 | .000 | 303.4234 | 526.5766 |
| AMU | KAB | 256.75000^*^ | 53.65254 | .000 | 145.1734 | 368.3266 |
|  | KIN | 287.00000^*^ | 53.65254 | .000 | 175.4234 | 398.5766 |
|  | BBS | 291.00000^*^ | 53.65254 | .000 | 179.4234 | 402.5766 |
|  | ATA | -264.50000^*^ | 53.65254 | .000 | -376.0766 | -152.9234 |
|  | KUT | -437.25000^*^ | 53.65254 | .000 | -548.8266 | -325.6734 |
|  | GUR | -22.25000 | 53.65254 | .683 | -133.8266 | 89.3266 |
| GUR | KAB | 279.00000^*^ | 53.65254 | .000 | 167.4234 | 390.5766 |
|  | KIN | 309.25000^*^ | 53.65254 | .000 | 197.6734 | 420.8266 |
|  | BBS | 313.25000^*^ | 53.65254 | .000 | 201.6734 | 424.8266 |
|  | ATA | -242.25000^*^ | 53.65254 | .000 | -353.8266 | -130.6734 |
|  | KUT | -415.00000^*^ | 53.65254 | .000 | -526.5766 | -303.4234 |
|  | AMU | 22.25000 | 53.65254 | .683 | -89.3266 | 133.8266 |
| ***The mean difference is significant at the 0.05 level.** | | | | | | |
