# Supplementary material for: Recrudescence of transmission of onchocerciasis in some endemic communities in Kaduna State, Nigeria: What is the way forward?
Source: PLoS Negl Trop Dis. 2025 Aug 7;19(8):e0012495. doi: 10.1371/journal.pntd.0012495 (PMC12331076; doi:10.1371/journal.pntd.0012495)
Supplement: Attachment — Submitted filename: PNTD-D-24-01243 edited.pdf [file pntd.0012495.s006.pdf]

PLOS Neglected Tropical Diseases

Recrudescence of transmission of onchocerciasis in Kaduna State, Nigeria: What is the way forward?

--Manuscript Draft--

**Manuscript Number:**	PNTD-D-24-01243
**Full Title:**	Recrudescence of transmission of onchocerciasis in Kaduna State, Nigeria: What is the way forward?
**Short Title:**	Recrudescence of transmission of onchocerciasis in Kaduna State, Nigeria
**Article Type:**	Research Article
**Keywords:**	Onchocerciasis; Kaduna State; recrudescence; Simulium damnosum s.l.; ivermectin; Onchocerca volvulus
**Abstract:**	Background<br><br>Human infection with the filarial parasite Onchocerca volvulus causes onchocerciasis and transmitted by Simulium damnosum s.l. (black fly) in Nigeria. Kaduna State in northern Nigeria was known for infestation of S. damnosum s.l. along rivers Gurara and Kaduna as earlier as 1951. The control of onchocerciasis in the State started in 1954 with larviciding [illegible]g dichlorodiphenyltrichloroethane (DDT) with 94% reduction in S. damnosum s.l. [illegible]ulation density at the end of 1966. Kaduna State Ministry of Health and its partners used annual mass administration of medicines (MAM) of ivermectin consistently in the State between 1989 and 2017 which led to interruption of onchocerciasis transmission in 2018. This study investigated possibility of recrudescence of onchocerciasis in the face of insecurity and human migration in Kaduna State four years after stop MAM decision.<br><br>Methodology/Principal Findings<br><br>Six of the 23 Local Government Areas (LGAs) in Kaduna State were selected for assessments. Dried blood samples (DBS) were collected from children 5-9 years and analysed using alkaline phosphatase enzyme-linked immunosorbent assay (AP-ELISA) technique. Adult S. damnosum s.l. were captured in seven breeding sites using consented human landing collectors (HLCs) and subsequently subjected to pool screening assay using quantitative polymerase chain reaction (qPCR) for detection of O. volvulus. Out of 3107 DBS collected, only 1502 were analysed because of devaluation of the naira, inflation and high cost of reagents. Two children in Kagarko LGA tested positive (prevalence 0.31; 95% upper confidence limit; UCL 0.317). Twenty out of 72 pools of heads of black flies analysed were positive 27.78% (95% UCL 0.49) black flies infectivity > 1/2000 infective flies in Kagarko and Kachia LGAs.<br><br>Conclusion/Significance<br><br>The results demonstrated ongoing transmission of onchocerciasis in Kaduna State. The possible causes, the implications on the onchocerciasis elimination programme and possible way forward were discussed.
**Additional Information:**	
**Question**	**Response**
**Government Employee**<br><br>Are you or any of the contributing authors an employee of the United States government?<br><br>Manuscripts authored by one or more US	No - No authors are employees of the U.S. government.

Powered by Editorial Manager® and ProduXion Manager® from Aries Systems Corporation

Government employees are not copyrighted, but are licensed under a CC0 Public Domain Dedication, which allows unlimited distribution and reuse of the article for any lawful purpose. This is a legal requirement for US Government employees.<br><br>This will be typeset if the manuscript is accepted for publication.	
**Financial Disclosure**<br><br>Enter a financial disclosure statement that describes the sources of funding for the work included in this submission. Review the submission guidelines for detailed requirements. View published research articles from *PLOS Neglected Tropical Diseases* for specific examples.<br><br>This statement is required for submission and **will appear in the published article** if the submission is accepted. Please make sure it is accurate.<br><br>**Funded studies**<br>Enter a statement with the following details:<br>• Initials of the authors who received each award<br>• Grant numbers awarded to each author<br>• The full name of each funder<br>• URL of each funder website<br>• Did the sponsors or funders play any role in the study design, data collection and analysis, decision to publish, or preparation of the manuscript?<br><br>Did you receive funding for this work?	Yes
Please add funding details.<br>as follow-up to "**Financial Disclosure**<br><br>Enter a financial disclosure statement that describes the sources of funding for the work included in this submission. Review the submission guidelines for detailed requirements. View published research articles from *PLOS Neglected Tropical*	This work received financial support from the Coalition for Operation Research on Neglected Tropical Diseases (COR-NTD), which is funded at The Task Force for Global Health primarily by the Bill and Melinda Gates Foundation and the United States Agency for International Development through its Neglected Tropical Diseases Program. The grant (SGP159/050.2023) was administered by the African Research Network for Neglected Tropical Diseases (ARNTD).

Powered by Editorial Manager® and ProduXion Manager® from Aries Systems Corporation

Diseases for specific examples.<br><br>This statement is required for submission and **will appear in the published article** if the submission is accepted. Please make sure it is accurate.<br><br>**Funded studies**<br>Enter a statement with the following details:<br>• Initials of the authors who received each award<br>• Grant numbers awarded to each author<br>• The full name of each funder<br>• URL of each funder website<br>• Did the sponsors or funders play any role in the study design, data collection and analysis, decision to publish, or preparation of the manuscript?<br><br>Did you receive funding for this work?"	
Please select the country of your main research funder (please select carefully as in some cases this is used in fee calculation).<br>as follow-up to "**Financial Disclosure**<br><br>Enter a financial disclosure statement that describes the sources of funding for the work included in this submission. Review the submission guidelines for detailed requirements. View published research articles from *PLOS Neglected Tropical Diseases* for specific examples.<br><br>This statement is required for submission and **will appear in the published article** if the submission is accepted. Please make sure it is accurate.	UNITED STATES - US

Powered by Editorial Manager® and ProduXion Manager® from Aries Systems Corporation

**Funded studies**<br>Enter a statement with the following details:<br>• Initials of the authors who received each award<br>• Grant numbers awarded to each author<br>• The full name of each funder<br>• URL of each funder website<br>• Did the sponsors or funders play any role in the study design, data collection and analysis, decision to publish, or preparation of the manuscript?<br><br>Did you receive funding for this work?"	
**Competing Interests**<br><br>On behalf of all authors, disclose any competing interests that could be perceived to bias this work.<br><br>This statement will be typeset if the manuscript is accepted for publication.<br><br>Review the instructions link below and PLOS NTDs' competing interests policy to determine what information must be disclosed at submission.	The authors declare no conflict of interest during this study.
**Data Availability**<br><br>Provide a **Data Availability Statement** in the box below. This statement should detail where the data used in this submission can be accessed. This statement will be typeset if the manuscript is accepted for publication.<br><br>Before publication, authors are required to make all data underlying their findings fully available, without restriction. Review our PLOS Data Policy page for detailed information on this policy. Instructions for writing your Data Availability statement can be accessed via the Instructions link below.	All relevant data are within the manuscript and its Supporting Information files.

Powered by Editorial Manager® and ProduXion Manager® from Aries Systems Corporation

Manuscript

Click here to access/download;Manuscript;Olanrewaju et al_manuscript.docx

Recrudescence of transmission of onchocerciasis in Kaduna State, Nigeria: What is the way forward?

Timothy O. Olanrewaju[1*], Felicia N. Enwezor[1], , Luret A. Lar[2], Michael A. Igbe[3], Ramatu A. Abdullahi[1], Monsuru A. Adeleke[4], Oluwatosin B. Adekeye[5], Elizabeth O. Elhassan[6]

[1]Nigerian Institute for Trypanosomiasis Research (*and Onchocerciasis*) Research, Kaduna, State, Nigeria

[2]Department of Community Medicine, Faculty of Clinical Sciences, College of Health Sciences, University of Jos, Plateau State, Nigeria

[3]Federal Ministry of Health, Federal Capital Territory, Abuja, Nigeria

[4]Department of Zoology, Faculty of Basic and Applied Sciences, Osun State University, Osogbo, Osun State, Nigeria

[5]Department of Psychiatry, Ahmadu Bello University Teaching Hospital Shika, Zaria, Kaduna State,

Nigeria

[6]1A Mohammed Yusuf Lere Drive, Off Tafawa Balewa Way, Ungwan Rimi, Kaduna State, Nigeria

Corresponding author: timothyseye@gmail.com (TOO)

Abstract

Background

Human infection with the filarial parasite *Onchocerca volvulus* causes onchocerciasis and transmitted by *Simulium damnosum s.l.* (black fly) in Nigeria. Kaduna State in northern Nigeria was known for infestation of *S. damnosum s.l.* along rivers Gurara and Kaduna as earlier as 1951. The control of onchocerciasis in the State started in 1954 with larviciding using dichlorodiphenyltrichloroethane (DDT) with 94% reduction in *S. damnosum s.l.* population density at the end of 1966. Kaduna State Ministry of Health and its partners used annual mass administration of medicines (MAM) of ivermectin consistently in the State between 1989 and 2017 which led to interruption of onchocerciasis transmission in 2018. This study investigated possibility of recrudescence of onchocerciasis in the face of insecurity and human migration in Kaduna State four years after stop MAM decision.

Methodology/Principal Findings

Six of the 23 Local Government Areas (LGAs) in Kaduna State were selected for assessments. Dried blood samples (DBS) were collected from children 5-9 years and analysed using alkaline phosphatase enzyme-linked immunosorbent assay (AP-ELISA) technique. Adult *S. damnosum s.l.* were captured in seven breeding sites using consented human landing collectors (HLCs) and subsequently subjected to pool screening assay using quantitative polymerase chain reaction (qPCR) for detection of *O. volvulus.* Out of 3107 DBS collected, only 1502 were analysed because of devaluation of the naira, inflation and high cost of reagents. Two children in Kagarko LGA tested positive (prevalence 0.31; 95% upper confidence limit; UCL [illegible]17). Twenty out of 72 pools of heads of black flies analysed were positive 27.78% (95% UCL 0.49) black flies infectivity > 1/2000 infective flies in Kagarko and Kachia LGAs.

Conclusion/Significance

The results demonstrated ongoing transmission of onchocerciasis in Kaduna State. The possible causes, the implications on the onchocerciasis elimination programme and possible way forward were discussed.

Keywords: Onchocerciasis, Kaduna State, recrudescence, *Simulium damnosum s.l.*, ivermectin, *Onchocerca volvulus*

Author summary

Onchocerciasis is a disease that affects humans and is transmitted by bites of *Onchocerca volvulus* infected black flies. Nigeria accounts for the highest disease burden of onchocerciasis in sub-Saharan Africa with about 50 million persons at risk living in over 40,000 communities in the nation. Collaborative effort by various stakeholders in Kaduna State, Nigeria yielded remarkable achievement of successful interruption of onchocerciasis transmission in year 2018 through annual mass administration of medicine (MAM). However, our study established the ongoing transmission of onchocerciasis in some areas of Kaduna State after four years stop MAM decision. Kaduna State Government, partners and stakeholders should urgently take actions to mitigate the ongoing transmission of onchocerciasis in the State to safeguard the achievements attained after years of MAM. We recommend the following; assessment of entire Kaduna State to determine the extent of the spread of the recrudescence; in-depth analysis of annual therapeutic coverage rates; community-driven slash and clear approach for vector control; review of the monitoring and evaluation system across all levels; using Esperanza Window Trap at black flies breeding sites to complement the slash and clear approach;

modeling the approaches to determine the most appropriate frequency of treatment with ivermectin.

Introduction

Human infection with the filarial parasite *Onchocerca volvulus* causes onchocerciasis (also known as river blindness). *O. volvulus* is transmitted to humans by bites of *Simulium damnosum s.l.* (black flies) in Nigeria. There are nine sibling species within the *S. damnosum* complex that can transmit onchocerciasis [1, 2]. Onchocerciasis is one of the Neglected Tropical Diseases (NTDs) with high public health impact in Africa. Over 99% of all cases of onchocerciasis and onchocercal-related blindness are found in Africa; with Nigeria having about 40% of the global population at risk [3]. Onchocercal blindness ranked second leading cause of preventable blindness in the world after trachoma [4]. The disease is peculiar to the poor with economic implications resulting in abandonment of rich fertile farmlands s which resulted in food insecurity and poor school attendance, sometimes forcing children to drop out of school to assist blind parents/guardians. Other manifestations include; skin disease disfiguration and stigmatization. These impact on the wellbeing of individuals with the disease become a major challenge to the achievement of Sustainable Development Goals (SDGs); “End hunger, achieved food security and improved nutrition and promote sustainable agriculture”(SDG 2) and “Ensure health lives and promote well-being for all at all ages (SDG3) as documented by Engels [5].

Crosskey [6] documented that adult *S. damnosum s.l.* dispersal ability estimated to be 234,000 Km^2 (90,000 square miles) in Northern Nigeria. Nearly half of the areas infested by *S. damnosum s.l.* in Northern Nigeria lies within region of rivers Gurara and Kaduna which is about

37,400 Km^2 (14,400 square miles). The control of onchocerciasis in Kaduna State started in 1954 with larviciding using dichlorodiphenyltrichloroethane (DDT) along rivers Gurara and Kaduna [7, 8]. By 1966, this control effort had achieved 94% reduction in *S. damnosum s.l.* population density. The State started annual MAM of ivermectin in 1989. Tekle et al. [9] reported that after 17years of consistent annual administration of ivermectin, two foci (Birnin Gwari and Kauru/Lere) in Kaduna State achieved onchocerciasis elimination. By 2018, interruption of transmission of the disease was attained and MAM implementation was stopped [10].

However, the current security challenges which revolve around ethno-religious and farmer-herder conflicts over land access heighten violence in Kaduna State [11]; migration to the State from the border States like Niger, Kano and Federal Capital Tertiary (FCT) Abuja where transmission of onchocerciasis is still ongoing [12] and vector migration due to climate change could cause recrudescence of onchocerciasis in Kaduna State. According to International Organization for Migration report [13], Kaduna State has a minimum of 117,880 internally displaced persons (IDPs) as a result of banditry and kidnapping (45%), communal clashes (29%), natural disaster (13%), farmers-herders clashes (12%), and insurgency (2%). Our interaction with the State Emergency Management Agency (SEMA) revealed that the only State government managed IDP camp is located at Giwa LGA. However, we identified a faith-based IDP camp at Agwan Zawu, Gonin-Gora, Chikun LGA and another at Evangelical Church Winning All (ECWA), Ungwan Musa, Zonkwa, Zangon-Kataf LGA. The high number of IDPs in Kaduna State without any organized or governmental camp, increase in inter-state migration within northwest region of Nigeria and climate change might affect sustainability of the interruption of transmission attained in onchocerciasis. These challenges could jeopardise efforts towards

2030 target of elimination of onchocerciasis, other targets in the NTD Roadmap 2021-2023 and DG 2 and 3.

We investigated possible recrudescence of onchocerciasis transmission in six LGAs in Kaduna State in the face of insecurity and human migration four years after interruption of transmission.

Methods

Study area

Kaduna State lies between 10°36'33" N and 7°25'46" E and shares boundaries with Kano and Katsina States in the north; Plateau State to the east; Nasarawa State to the south; Niger State in the west and the FC) Abuja to the southwest. Some of the major rivers in the State include river Gurara and river Kaduna which serve as good breeding sites for black flies [14]. All of the States that share boundaries with Kaduna are currently treating with ivermectin except Plateau and Nasarawa States, which have interrupted transmission, and Katsina State which was hypo endemic for onchocerciasis and was ineligible for treatment requires onchocerciasis elimination mapping (OEM). Kaduna State consists of 23 LGAs with a projected population of more than six million people and estimated land area of about 46,063 Km^2. The demographic vegetation of the State is mainly savannah grassland with few areas of forest. This study was carried out in six LGAs of Kaduna State, namely Chikun, Kachia, Kaduna North, Kaduna South, Kagarko and Zangon-Kataf (Fig 1). Kaduna North and Kaduna South LGAs were selected due to the presence of black fly breeding sites at river Kaduna that runs through the two LGAs and influx of migrants from Chikun LGA. Chikun, Kachia and Kagarko were included because of the presence of black

flies breeding sites along rivers Gurara and Kaduna; insecurity and presence of migrants. Zangon-Kataf LGA was chosen based on high level of insecurity and proximity to breeding site in Kachia. Seven breeding sites across five LGAs serve as catching points of black flies namely Kabala Doki, Kwata, Tsallake, Atara, Dogon-Daji, Amuse and Gurara.

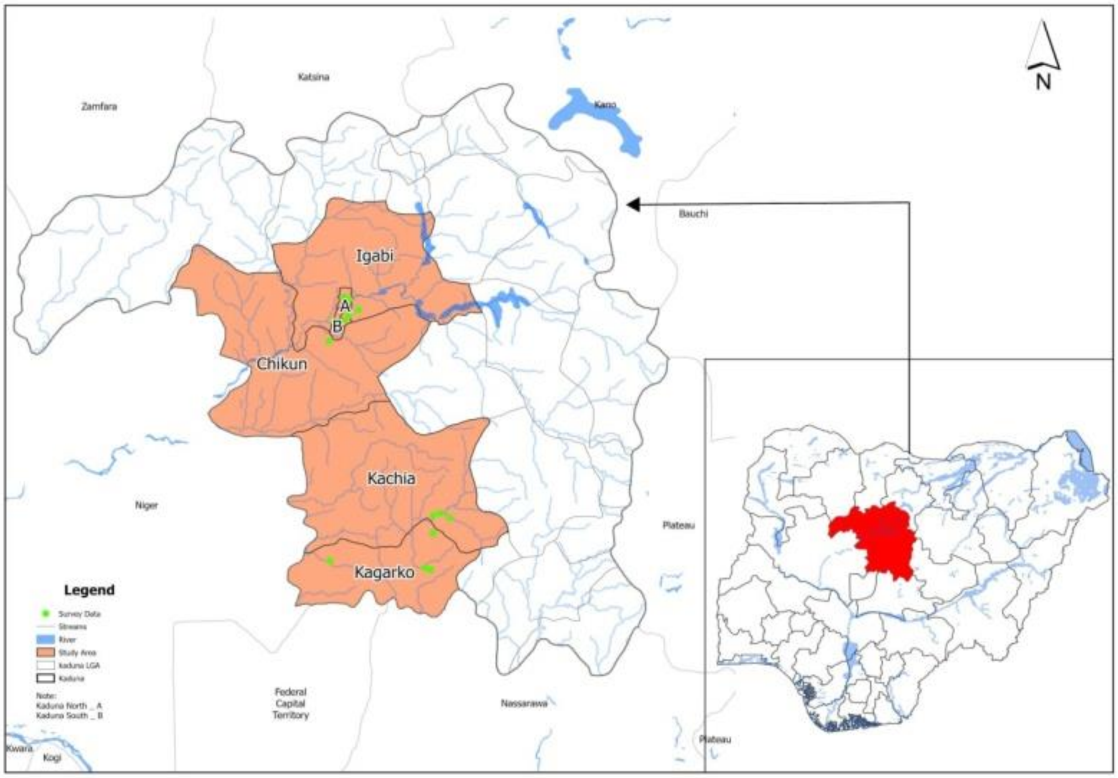

Fig 1: Map of Kaduna State showing the study areas

Epidemiological evaluation

The epidemiological study was carried out in all the selected LGAs. Sample collection was done between July and October, 2023.

Study population

Farming is the major occupation of citizens at Chikun, Kagarko, Kachia and Zangon-Kataf, but for Kaduna South and Kaduna North LGs majority are civil servants and business men/women. Dried blood samples were collected from children (male and female) between 5 and 9 years old across the study areas.

Sample size and sample collection

Dried blood samples were collected from 3107 children aged 5-9 years from various communities and primary schools across the six LGAs using World Health Organization (WHO) guidelines for epidemiological assessment of onchocerciasis transmission in children [15, 16]. The sample from each community was estimated proportionally to available children within the required age group. Blood samples were collected from all assented children aged 5-9 years through finger-prick using a sterile lancet each for a child after swabbing the finger with methylated spirit. About 100 µL of blood from each child was spotted on Whatman number 1 filter paper (Whatman International Ltd Maldstone, England); using one filter paper for a child. Each of the filter papers was fixed to a Styrofoam using pencil for air-drying. For each community, each child's sample was stored in a small ziplock bag and then put into a larger ziplock bag containing silica gel (Desicare, Reno, NV, USA), labeled appropriately and store at 25°C.

Sample analysis

The samples were analysed within maximum of six months of sample collection at Osun State University Multi-Disciplinary Research Laboratory, Osogbo, Nigeria using AP-ELISA technique as detailed in S1 Methods. Only 1502 DBS from first-line and second-line communities to black fly

breeding sites; communities with IDPs and migrants were analysed because of devaluation of the naira, inflation and high cost of reagents. The Ov16 antibody prevalence in children was determined using 95% confidence intervals of > 0.1% upper confidence limit (UCL) as described [17] using the formula:

95% Confidence interval = proportion positive (p) ± 1. 96 $\left(\frac{\sqrt{P(1-\mathrm{P})}}{\mathrm{n}}\right)$

Ethical approval

The study protocol was approved by the Health Research Ethics Committee (HREC) of Kaduna State Ministry of Health (Ref No. NHREC/17/03/2018). Verbal assent was obtained from each child with consent of either parent or guardian of the child before blood sample collection. Volunteers that acted as human attractants for black fly catching were told about the personal risks and community benefits of participation and given the option to opt out of participation at any time without any repercussions. They were offered ivermectin at the end of the study according to specifications.

Entomological evaluation

Seven known breeding sites of *S. damnosum s.l.* in five LGAs – Chikun, Kachia, Kaduna North, Kaduna South and Kagarko were assessed to ascertain if they were currently breeding the vector. The coordinates of the breeding sites were taken using Garmin™ geographical positioning information system (GPIS) machine. Based on the outcome of the assessments, community leaders of first-line and second-line communities of the seven productive sites for *S. damnosum s.l.* were sensitized on the importance of preventing blindness due to onchocerciasis. The leaders identified two persons from their communities that serve as HLCs for *S. damnosum*

s.l. The HLCs were clustered into two groups for a two days theory and practical training on capturing of black flies and recording as described [18, 19]. Black fly capturing activities were conducted between July and October 2023 being the peak of black fly breeding season. Two HLCs worked in an alternate arrangement hourly for two days per week between 07:00 h and 18:00 h. The flies collected were stored in preservative bottles containing isopropyl alcohol. The bottles were labeled accordingly for each catching site. At the end of capturing of the flies, the study entomologist sorted all the flies and confirms the flies that were *S. damnosum s.l.* morphologically. The black flies were hand carried to the Multi-disciplinary Research Laboratory, Osogbo, Nigeria to determine the *O. volvulus* infectivity rate in black flies using real-time qPCR technique assay with a mitochondrial DNA target as detailed in S2 Methods. The separation of the heads from the bodies, extraction and qPCR were carried out as described [20]. The poolscreen software 2.1 was used to determine *O. volvulus* infectivity rate in pools of black flies at 95% confidence interval.

Results

Out of the 1,502 DBS of children aged 5 – 9 years; two children from Kagarko LGA were Ov16 sero-positive with prevalence of 0.13% (95% UCL 0.317) as shown in Tables 1 & 2. These children were from first-line and second-line communities, black fly breeding sites; and communities with IDPs and migrants. The two Ov16 sero-positive samples were from Atara community (first-line community) and Kubacha community (second-line community) within Kagarko LGA.

Table 1: Blood sample collected from children aged 5-9 years at selected schools/communities

Local Government Area	Community	Number of children sampled	Total number of children sampled in each LGA
**Kaduna North**	Angwan Gwari	136	706
	Rafin Guza	160	
	Kawo	106	
	Kabala	304	
**Kaduna South**	Down Quarters	136	955
	Kinkinau	176	
	Television	134	
	Tudu Wada	276	
	Barnawa	233	
**Chikun**	Babban Saura	237	519
	Doka Mai Jama'	137	
	Bagado	122	
	Angwan Zawu IDP camp	23	
**Kagarko**	Kuratam	231	478
	Atara	76	
	Kubacha	100	
	Dogo Kurumi	71	
**Kachia**	Gurara	49	49
**Zango-Kataf**	Zonkwa	262	400
	Samaru-Kataf	138	
**TOTAL**	**20**	**3,107**	**3,107**

Table 2: Dried blood sample Ov16 antibody/antigen test using AP-enzyme linked immunosorbent assay

Local government area	Community	Proximity to black fly breeding site	No. of DBS analysed	No. of positive DBS
	Kabala Doki	First line	86	0
	Angwan-Gwari	Second line	136	0
**Kaduna North**	Rafin-Guza	Second line	100	0
	Kinkunu	First line	100	0
**Kaduna South**	Down Quarters	Second line	135	0
	Angwan Zawu IDP camp	Second line	23	0
	Doka Mai Jama'	First line	137	0
**Chikun**	Babban Saura	First line	120	0
	Kuratam	First line	231	0
	Atara	First line	76	1
	Kubacha	Second line	100	1
**Kagarko**	Dogo Kurumi	Second line	71	0
**Kachia**	Gurara	First line	49	0
**ZangonKataf**	Samaru Kataf	Second line	138	0
**TOTAL**			**1,502**	**2**
**Lower and Upper Limit CI (95%)**				**-0.051 - 0.368**

The 95% UCLof OV-16 positivity > 0.1% indicates ongoing transmission of Onchocerciasis in Kaduna State

Six thousand eight hundred and ninety-seven (6,897) flies out of 7,782 flies captured were identified as *S. damnosum s.l*. (Table 3). The highest number of black flies captured was from Kagarko LGA (71.48%) with 2,858 flies at Kuratam and 2,072 at Atara. This was followed by 1963 catches from Kachia LGAs of which 1181 flies were from Gurara and 782 from Amuse. Only four black flies were captured along river Kaduna; two each at Kabala-Doki and Kinkinau. Twenty pools of the 72 pools of heads (27.8%) of *S. damnosum s.l.* analysed were positive for *O. volvulus* at 95% UCL 0.49 of black flies infectivity (> 1/2000 infective flies) (Table 4). Four out of

the seven black flies catching sites (57.14%) tested positive with both sites in Kagarko and Kachia LGAs testing positive. We observed the highest infectivity rate (33%) in black flies captured at Amuse catching site in Kachia LGA.

Table 3: Human landing capturing of flies at various breeding sites in the study areas

Local Government Area	Community	River	Catching Site	Number of fly catchers	Number of unsorted flies captured	Number of sorted black flies	Number of pool
**Kaduna North**	Kabala	Kaduna	Kabala	2	148	2	1
**Kaduna South**	Kinkinau	Kaduna	Kwata	2	27	2	1
**Chikun**	Babban Saura	Kaduna	Tsallake	2	11	0	0
	Atara	Gurara	Atara	2	2233	2072	21
**Kagarko**	Kuratam	Rafin-Taba	Dogon Daji	2	2924	2858	29
	Amuse	Gurara	Gurara	2	1175	782	8
**Kachia**	Gurara	Gurara	Gurara	2	1264	1181	12
**TOTAL**			**7**	**14**	**7,782**	**6,897**	**72**

Table 4: Real time quantitative polymerase chain reaction assay of *S. damnosum s.l.*

Local government area	Community	No. of black flies analysed	No. of pools of 100	No. of pools < 100	No. of positive pools
**Kaduna North**	Kabala Doki	2	0	1(2)	0
**Kaduna South**	Kinkinau	2	0	1(2)	0
**Chikun**	Tsalake	0	0	0	0
**Kagarko**	Kuratam	2,858	28	1(58)	7(24%)
	Atara	2,072	20	1(72)	7(33%)
**Kachia**	Gurara	1,181	11	1(81)	3(25%)
	Amuse	782	7	1(82)	3(38%)
**TOTAL**		**6,897**	**66**	**6**	**20**
**Lower and Upper Limit CI (95%)**				**0.21 – 0.49**	

The 95% UCL of black flies infectivity of >1/2,000 infective flies/2,000 indicates ongoing transmission of onchocerciasis in Kaduna State most especially in areas with insecurity

Discussion

Our investigation on sustained interruption of transmission of *O. volvulus* in Kaduna State amidst insecurity and migration revealed *S. damnosum* infectivity rate of 27.8% (C.I. 0.21-0.49) which is more than the WHO threshold of <1/2000 infective rate in black flies. Davies et al. (1968) carried out larviciding using DDT along river Gurara from Suleja, Abuja to Tafa. This resulted to 94% reduction in the population density of *S. damnosum* in the areas. However, Tafa is about 25 km from Kagarko and Kachia breeding sites; the large number of fly recorded suggests that breeding of black fly continues along river Gurara. On the other hand, the high water level in river Kaduna resulted in low fly density in Chikun, Kaduna North and Kaduna South LGAs despite a four months attempt. The low black fly population in these areas may be the effect of urbanization, climate change or pollution. Seasonal fluctuations in black flies and high rate of infective larvae along river Kaduna has been documented [21]. Changes in the geographical distributions of different cytospecies of the *Simulium damnosum* complex was also observed in Togo and Benin [22].

Though the breeding sites along river Gurara were probably unknown during cytotaxonomic identification of members of *S. damnosum* complex [23], breeding of black fly and transmission of onchocerciasis along the river at Gantang in Kagarko LGA has been documented [14]. The high population density of black fly recorded in Amuse, Gurara, Atara and Kuratam sites could be as a result of vegetation, rocks, speed of the water and other favourable physico-chemical parameters of the river Gurara [24, 25]. The population density along river Gurara corroborates the earlier report [14]. The high infectivity rate in black flies we observed in Amuse, Atara, Gurara and Kuratam suggests that transmission cycle of onchocerciasis has been sustained for

awhile in Kachia and Kagarko LGAs. This contradicts the previous documentation [10] that no transmission of *O. volvulus* in Kaduna State; thus recrudescence of onchocerciasis established in the State. There is possibility of imported infection from the neigbouring Niger State and FCT Abuja to Kaduna State. According to Crosskey [6], rivers Gurara, Kaduna, Sarkin Pawa (in Niger State) are always infested with black fly with rivers Gurara and Kaduna form major fly area of approximately 140 miles (225 km). The ability of *S. damnosum* complex to migrate >400 km away in large numbers [1, 26, 27] can threaten the interruption of transmission of onchocerciasis and elimination of the disease in Kaduna State.

To curb the black fly population density, slash and clear can be adopted. This approach requires removal of vegetation from breeding sites (fast flowing well oxygenated sediment free water) along rivers to disrupt *S. damnosum* breeding activities. Smith et al. [28] recommended slash and clear using community-driven implementation strategy at least once a year for accelerating achievement of onchocerciaisis elimination. Slash and clear vector control using community-driven implementation strategy at Maridi dam spillway in South Sudan reduced *S. damnosum* by >90% six months post intervention and <50% twelve months post intervention [29]. The approach has also been used in Uganda with 97% reduction in biting rate after single intervention during early wet season [30] and Cameroon with 32.9% reduction in black fly population density [31]. Combination of CDTi with slash and clear vector control will accelerate achievement of onchocerciasis in Africa. The use of Esperanza Window Trap can also be deployed along black flies breeding sites to complement the slash and clear approach as described [32].

Despite the on-going treatment with ivemectin for Lymphatic filariasis in Kachia and Kagarko LGAs after stopping treatment for onchocerciasis in 2018, the 0.13% Ov16 serological test result in children aged 5-9 years we obtained in this study indicates ongoing transmission of onchocerciasis in human. This implies that Kaduna State could not sustain the interruption of transmission of onchocerciasis recorded in year 2018. Reason for the ongoing transmission could be the insensitivity of the O-150 PCR used in earlier study [10] compared to real-time qPCR assay with a mitochondrial DNA target (OvND5) used in this study. The sensitivity of OvND5 has been reported elsewhere [33] and recrudescence of onchocerciasis in Nasarawa and Abia States [32]. Also, climate change with great impact on black flies' movement and human migration (intra and interstate) as a result of communal conflicts, political crisis, kidnapping and insurgency could setback elimination of onchocerciasis [34, 35, 36]. The earlier therapeutic coverage of 65% with ivermectin attained in Kaduna State before stopping MAM [10] might be insufficient to break the transmission cycle considering the high infectivity rate of black flies in some part of the State.

Kagarko, Kachia and other LGAs in southern part of Kaduna State have had several episodes of insecurity since 2011 [37]. During the period of insecurity, the population in the affected communities moved out to other LGAs within the State and Niger State then returned when the situation is calm. The migration of people from affected communities poses a threat to elimination of onchocerciasis in other LGAs that share boundaries with the LGAs in Kaduna; Tafa and Suleja in Niger States and Bwari in FCT, Abuja. This calls for a review of geographic and therapeutic coverage rates, implementation of community self monitoring and intensify monitoring and evaluation of treatment. There is need for development of model that will

determine appropriate frequency of treatment for Kaduna State. Adoption of biannual treatment in River Gambia, Senegal (April or May and October or November) for more than 10 years has been successfully implemented with desired outcomes [38]. Also, the biannual community directed treatment with ivermectin resulted to elimination of onchocerciasis in Colombia in 2013 [39]; Ecuador in 2014 [40] and Guatemala in 2016 [41]. To achieve elimination of onchocerciasis, Mexico changed frequency of treatment with ivermectin to quarterly and achieved elimination in year 2015 [42].

In conclusion, our study has established ongoing transmission of onchocerciasis and recrudescence of the disease after interruption of transmission in the study areas. This study only covered the southern and central Kaduna onchocerciasis transmission foci. Other transmission foci in Birnin Gwari and Lere/Saminaka foci were not included in the study due to limited research funding. We suggest a multi-pronged approach to address the recrudescence of onchocerciasis in Kaduna State as follows:

(1) Assessment of the entire State to determine the extent of recrudescence and to develop frame work for onchocerciasis elimination

(2) In-depth analysis of therapeutic and geographic coverage rates to identify if there are any lapses in the rates.

(3) Adoption of Slash and clear of S. damunsum s.l. breeding sites using community-driven implementation.

(4) Deployment of Esperanza Window Trap can be deployed to black flies breeding sites to complement the slash and clear approach.

(5) Implementation of community self monitoring of treatment coverage.

(6) Commencement of multiple treatments with Ivermectin.

(7) Treatment of adult population with Moxidectin can be used in areas with high black flies infectivity rate. Moxidectin has a rapid absorption, high volume of distribution and longer half-life [43].

(8) Modeling of the above approaches to determine the most appropriate frequency of treatment with ivermectin and combination of vector control. Multiple treatments with ivermectin have successfully eliminated onchocerciasis in Colombia, Ecuador, Mexico and Guatemala.

Kaduna State Government, partners and stakeholders should urgently take actions to mitigate the ongoing transmission of onchocerciasis in the State to safeguard the achievement attained after years of MAM.

Supporting information

S1 Methods: Detailed protocol for Ov16 ELISA

S2 Methods: Detailed protocol for DNA isolation from black fly heads and qPCR

Author contributions

Conceptualization: Felicia N. Enwezor, Timothy O. Olanrewaju, Elizabeth O. Elhassan

Data Curation: Timothy O. Olanrewaju, Felicia N. Enwezor, Oluwatosin B. Adekeye

Methodology: Timothy O. Olanrewaju, Felicia N. Enwezor, Michael Igbe

Project Administration: Timothy O. Olanrewaju, Felicia N. Enwezor

Funding Acquisition: Timothy O. Olanrewaju, Felicia N. Enwezor, Elizabeth O. Elhassan, Luret A. Lar, Oluwatosin B. Adekeye

Investigation: Timothy O. Olanrewaju, Felicia N. Enwezor, Michael Igbe, Ramatu A. Abdullahi

Formal Analysis: Monsuru A. Adeleke, Michael Igbe

Supervision: Felicia N. Enwezor, Elizabeth O. Elhassan, Luret A. Lar

Validation: Monsuru A. Adeleke, Luret A. Lar, Felicia N. Enwezor, Oluwatosin B. Adekeye

Writing-Original Draft: Timothy O. Olanrewaju, Michael Igbe, Luret A. Lar

Writing-Review and Editing: Timothy O. Olanrewaju, Felicia N. Enwezor, Luret A. Lar, Ramatu A. Abdullahi, Micheal Igbe, Monsuru A. Adeleke, Oluwatosin B. Adekeye, Elizabeth O. Elhassan.

Funding

This work received financial support from the Coalition for Operation Research on Neglected Tropical Diseases (COR-NTD), which is funded at The Task Force for Global Health primarily by the Bill and Melinda Gates Foundation and the United States Agency for International Development through its Neglected Tropical Diseases Program. The grant (SGP159/050.2023) was administered by the African Research Network for Neglected Tropical Diseases (ARNTD). The funders had no role in study design, data collection and analysis, decision to publish, or preparation of the manuscript.

Acknowledgment

We acknowledge Sightsavers, Kaduna Office and Kaduna State Ministry of Health for availing us essential information during the design of the study protocols and sample collection. Also, Nigerian Institute for Trypanosomiasis Research, Kaduna for the provision of the vehicle used during field activities. Osun State University Multi-disciplinary Research Laboratory, Osogbo, Nigeria provided additional reagents for Ov16 serological test and qPCR analysis of *S. damnosum s.l.*

Conflict of Interest

The authors declare no conflict of interest during this study.

References

1. Post KJ, Onyenwe E, Somiari SA, Mafuyai HB, Crainey JL, Ubachakwu PO. A guide to the *Simulium damnosum* complex (Diptera: Simuliidae) in Nigeria, with a cyto taxonomic key for the identification of the sibling species. *Ann. Trop. Med Parasitol*. 2011 Jun 1; 105(4):277-297. https://doi.org/10.1179/136485911x12987676649700PMID: 21871165
2. Chikekie FM, Opara KN, Ubulom PME, Yaro CA, Al-Akeel RK, Osei-Atweneboana MY et al. Onchocerciasis transmission status in some endemic communities of mass drug administration with ivermectin.*Sci Rep*. 2023; 13:5413doi: 10.1038/s41598-023-31446-6PMID: 37012274
3. World Health Organization. Ending the neglect to attain the Sustainable Development Goals: A roadmap for neglected tropical diseases 2021–2030. Available: https://apps.who.int/iris/rest/bitstreams/1326801/retrieve [Accessed June 15, 2023].
4. World Health Organization. Onchocerciasis: Fact sheet 2022. https://www.who.int/news-room/fact [Accessed September 12, 2023]
5. Engels D. Neglected tropical diseases in the Sustainable Development Goals. *The Lancet* 2016 January 16;387(10015), 223-224. https://doi:1016/s104-6736(16)00043-x
6. Crosskey RW. The distribution of *Simulium damnosum* Theobald in northern Nigeria. *Trans. Royal Society Trop. Med. Hyg*. 1956; 50: 379–392
7. Davies JB. The *Simulium* control scheme at Abuja, Northern Nigeria and its effect on the prevalence of onchocerciasis in the area. *Bull. World Health Org*. 1968; 39:187-207

8. Bump JB. The Crosskey-Davies Experiment and Onchocerciasis Control in West Africa. *PLoS Negl Trop Dis* 2014 October 9; 8(10): e3223. https://doi:10.1371/journal.pntd.0003223
9. Tekle AH, Elhassan E, Isiyaku S, Amazigo UV, Bush S, et al. Impact of long-term treatment of onchocerciasis with ivermectin in Kaduna State, Nigeria: first evidence of the potential for elimination in the operational area of the African Programme for Onchocerciasis Control. *Parasit Vectors* 2012 Feb 7; 5:28. https://doi:10.1186/1756-3305-5-28
10. Isiyaku S, Igbe M, Madaki S, Hamill LC, Ndongmo P, Adamani W, et al. The interruption of transmission of onchocerciasis in Kaduna, Kebbi and Zamfara State, Nigeria: another milestone achievement. *Intern. Health* 2022 May 12; 14(2):ii43-ii54. https://doi.org/10.1093/inthealth/ihac036
11. Olajumoke A. (2021). Confronting Nigeria's Kaduna crisis https://africacenter.org/spotlight/confronting-nigerias-kaduna-crisis [Accessed February 20, 2022].
12. Yahaya A, Kela SL, Suleiman MM, Sama'ila AB. Prevalence studies on Onchocerciaisis (River Blindness) in some villages from Three Senatorial Districts, Kano State, Nigeria. *South Asian Journal Parasitol.* 2021*;* 5(4):141-152; Article no. SAJP 80294
13. Displacement Tracking Matrix (DTM) of the International Organization for Migration (IOM). IOM Nigeria-North-central and North-west zones displacement report. 2023; Round 12 needs monitoring. Available: https://dtm.iom.int/Nigeria [Accessed May 8, 2024]
14. Isiyaku S, Adamani W, Louise H, Ndams IS, Ezekiel K, Kal, A, et al. Entomological assessment of the Impact of long-term mass administration of ivermectin in onchocerciasis-endemic communities in Kaduna State, Nigeria. [Preprint]. 2021 [cited 2022 August 21]. Available from: https://doi.org/10.21203/rs.3.rs-840823/v1
15. World Health Organization. Guidelines for Stopping Mass Drug Administration and Verifying Elimination of Human Onchocerciasis: Criteria and Procedures. *Geneva, Switzerland*: WHO 2016.[Google Scholar]
16. Nigerian Federal Ministry of Health. Nigeria Onchocerciasis elimination plan. *Abuja: Federal Ministry of Health*; 2017.
17. Richard FO, Katabarwa M, Bekele F, Tadesse Z, Mohammed A, Sauerbrey M, *et al*. Operational performance of the *Onchocerca volvulus* "OEPA" OV16 ELISA serological assay in mapping, stop mass drug administration and post treatment surveillance survey. *Am J Trop Med Hyg.*2018;99(3):747–752 doi: 10.4269/ajtmh.18-0341
18. Service MW. Methods for sampling adult *Simuliidae,* with special reference to the *Simulium damnosum* complex*. Tropical Pest Bulletin* 1977 (5). Centre for Overseas Pest Research, London, UK; ISBN 0-85135-087-9 http://gala.gre.ac.uk/12219
19. Adeleke MA, Mafiana CF, Sam-Wobo SO, Olatunde GO, Ekpo UF,Akinwale OP et al. Biting behaviour of *Simulium damnosum* complexand *Onchocerca volvulus* infection along Osun River, Southwest Nigeria. *Parasit Vector 2010 October 7;* 3(93): 1-5. doi:10.1186/1756-3305-3-93 http://www.parasitesandvectors.com/content/3/1/93

20. Hendy A, Kruger A, Pfarr K, De Witte J, Kibweja A, Mwingira U, et al. The black fly vectors and transmission of *Onchocerca volvulus* in Mahenge, south eastern Tanzania. *Acta Trop.* 2018 February 2; 181:50–9. https://doi.org/10.1016/j.actatropica.2018.01.009
21. Gaiya DD, Jonathan B, Udu SK, Hassan G. Possible recrudescence of onchocerciasis within Kaduna metropolis: microfilariae load and transmission potential posed by blackfly. DYSONA-Life Sci. 2021; 2:33-38
22. Cheke RA, Post RJ, Boakye DA. Seasonal variations and other changes in the geographical distributions of different cytospecies of the *Simulium damnosum* complex (Diptera: Simuliidae) in Togo and Benin. Acta Trop. 2023 June 18; 245. https://doi.org/10.1016/j.actatropica.2023.106970
23. Mafuyai HB, Post RJ, Vajime CG, Molyneux DH. Cytotaxonomic identifications of the *Simulium damnosum* complex (Diptera: Simuliidae) from Nigeria. *Trop. Med Intern. Health* 1996; 1(6):779-785
24. Ibeh OO, Nwoke BEB, Adegoke JA. Distribution and ecology of breeding sites of *Simulium damnosum s.l*. in south–eastern primary health zone of Nigeria. Nigerian Journal of Parasitology 2007; 28:32–38.
25. Oforka LC, Adeleke MA, Anikwe JC, Makanjuola WA. Population fluctuations and effect of climatic factors on the relative abundance of *Simulium damnosum* complex (Diptera: Simuliidae). Environ. Entomol. 2019; 48: 284–290.
26. Walsh JF, Davies JB, Garms R. Further studies on the reinvasion of the onchocerciasis control programme by *Simulium damnosum s.l.*: the effects of an extension of control activities into southern Ivory Coast during 1979. Tropenmedizin und Parasitologie 1981; 32, 269–273.
27. Baker RHA, Guillet P, Se´ke´te´li A, Poudiougo P, Boakye D et al. Progress in controlling the reinvasion of windborne vectors into the western area of the Onchocerciasis Control Programme in West Africa. Philosophical Transactions of the Royal Society of London. 1990; Series B, 328, 731–750.
28. Smith ME, Bilal S, Lakwo TL, Habomugisha P, Tukahebwa E, Byamukama E, et al. Accelerating river blindness elimination by supplementing MDA with a vegetation “slash and clear” vector control strategy: a data-driven modeling analysis. *Sci Rep*. 2019; 9:15274. https://doi:10.1038/s41598-019-51835-0
29. Raimon S, Lakwo TL, Sebit WJ, Siewe Fodjo JN, Alinda P, et al. “Slash and Clear”, a Community- Based Vector Control Method to Reduce Onchocerciasis Transmission by Simulium sirbanum in Maridi, South Sudan: A Prospective Study. Pathogens 2021 October 15; 10, 1329. https://doi.org/10.3390/pathogens10101329
30. Jacob B, Loum D, Munu D, Lakwo T, Byamukama, E, Habomugisha P, et al. Optimization of Slash and Clear Community-Directed Control of *Simulium damnosum Sensu Stricto* in Northern Uganda. Am. J. Trop. Med. Hyg. 2021, 104, 1394–1403. https://doi:10.4269/ajtmh.20-1104
31. Domche A, Djeunga HCN, Nwane PB, Njitchouang GR, Fesuh BN, Njiokou F, et al. Significant reduction of blackfly densities in persistent onchocerciasis area following pilot implementation of an environment friendly approach (slash and clear). *Sci. Reports* 2024; 14:408 https://doi.org/10.1038/s41598-023-50747-4

32. Adeleke MA, Opara KN, Mafuyai HB, Nwoke beb, Surakat OA, Akinde SB, et al. Improving onchocerciasis elimination surveillance: trials of odour baited Esperanza Window Traps to collect black fly vectors and real-time qPCR detection of Onchocerca olvulus in black fly pools. [Preprint]. 2024 [Cited 2024 August 16]. Available from: https://doi.org/10.21203/rs.3.rs-4226239/v1
33. Doherty M, Grant JR, Pilotte N, Bennuru S, Fischer K, Fischer PU, et al. Optimized strategy for real-time qPCR detection of *Onchocerca volvulus* DNA in pooled *Simulium sp*. blackfly vectors. *PLoS Negl Trop Dis* 2023 17(12):e0011815. https://doi.org/10.1371/journal. pntd.00118 15
34. Coninx R. Tuberculosis in complex emergencies. *Bull World Health Organ.* 2007; 85:637–40.
35. Meteke S, Stefopulos M, Als D, Gaffey M F, Kamali M, *et al*. Delivering infectious disease interventions to women and children in conflict settings: a systematic review. *BMJ Global Health* 2020; 5:e001967. https://doi:10.1136/bmjgh-2019-001967
36. Lakwo T, Oguttu D, Ukety T, Post R, Bakajika D. Onchocerciasis Elimination: Progress and Challenges. *Res Rep Trop Med.* 2020;11:81-95 http://doi.org/10.2147/RRTM.S224364
37. News. We've paid some Fulani to stop killings in Southern Kaduna - El-Rufai. Vanguard Newspaper 2016 December 3 [Cited 2023 September 10]. Available from https://www.google.com/url?sa=t&source=web&rct=j&opi=89978449&url=https://www.vanguardngr.com/2016/12/weve-paid-fulani-stop-killings-southern-kaduna-el-rufai/&ved=2ahUKEwjFuLv1s5OIAxVyQkEAHVPaCngQFnoECB0QAQ&usg=AOvVaw2jsptX99yvtN0xdxpwb3MT
38. Traore MO, Sarr MD, Badji A, Bissan Y, Diawara L, Doumbia K, et al. Proof-of-Principle of Onchocerciasis Elimination with Ivermectin Treatment in Endemic Foci in Africa: Final Results of a Study in Mali and Senegal. PLoS Negl Trop Dis 2012; 6(9): e1825. https://doi:10.1371/journal.pntd.0001825
39. Nicholls RS, Duque S, Olaya LA, Lopez MC, Sanchez SB, Morales AL, et al. Elimination of onchocerciasis from Colombia: first proof of concept of river blindness elimination in the world. *Parasit. Vectors* 2018 April 11; 11:237 https://doi.org/10.1186/s13071-018-2821-9
40. Guevara A, Lovato R, Proaño R, Rodriguez-Perez MA, Unnasch T, Cooper PJ, et al. Elimination of onchocerciasis in Ecuador: findings of post-treatment surveillance. *Parasit Vector* 2018 April 24e; 11:265 https://doi.org/10.1186/s13071-018-2851-3
41. Richard F, Rizzo N, Espinoza CED, Monroy ZM, Valdez CGC, de Cabrera RM, et al. One hundred years after its discovery in Guatemala by Rodolfo Robles, *Onchocerca volvulus* transmission has been eliminated from the Central Endemic Zone. Am. J. Trop. Med. Hyg. 2015 October 26, 93(6):1295–1304. https://doi:10.4269/ajtmh.15-0364
42. Rodriguez-Perez MA, Fernandez-Santos NA, Orozco-Algarra ME, Rodríguez-Atanacio JA, Domínguez-Vázquez A, Rodríguez-Morales KB, et al. Elimination of onchocerciasis from Mexico. *PLoS Negl Trop Dis*. 2015; 9(7):e0003922. https://doi:10.1371/journal.pntd.0003922
43. Milton P, Hamley JID, Walker M, Basáñez MG. Moxidectin: an oral treatment for human onchocerciasis, Expert Review of Anti-infective Therapy 2020 July 26; https://doi.org/10.1080/14787210.2020.1792772

Click here to access/download
Supporting Information
Supporting information S1 _Methods_Ov16 ELISA.docx

Click here to access/download
Supporting Information
Suppoting information S2_Methods_qPCR.docx
