# Supplementary material for: Recrudescence of transmission of onchocerciasis in some endemic communities in Kaduna State, Nigeria: What is the way forward?
Source: PLoS Negl Trop Dis. 2025 Aug 7;19(8):e0012495. doi: 10.1371/journal.pntd.0012495 (PMC12331076; doi:10.1371/journal.pntd.0012495)
Supplement: Attachment — Submitted filename: PNTD-D-24-01243_R1 FMC edited.pdf [file pntd.0012495.s009.pdf]

PLOS Neglected Tropical Diseases

Recrudescence of transmission of onchocerciasis in some endemic communities in Kaduna State, Nigeria: What is the way forward?

--Manuscript Draft--

**Manuscript Number:**	PNTD-D-24-01243R1
**Full Title:**	Recrudescence of transmission of onchocerciasis in some endemic communities in Kaduna State, Nigeria: What is the way forward?
**Short Title:**	Recrudescence of transmission of onchocerciasis in Kaduna State, Nigeria: The way forward
**Article Type:**	Research Article
**Keywords:**	Onchocerciasis; Kaduna State; recrudescence; Simulium damnosum s.l.; ivermectin; Onchocerca volvulus
**Abstract:**	Background<br>Onchocerciasis caused by the filarial parasite Onchocerca volvulus and transmitted by Simulium damnosum s.l. remains a public health concern in Nigeria. Infestation of S. damnosum s.l. along rivers Gurara and Kaduna; and heavy intensity of O. volvulus infection in Kaduna were documented in 1956. Control of onchocerciasis in Kaduna started 1954 with larviciding using dichlorodiphenyltrichloroethane which reduced S. damnosum s.l. population by 94% in 1966. Diethylcarbamazine was also used for human treatment but discontinued. Kaduna State Ministry of Health and its partners built on this achievement; used annual mass administration of ivermectin consistently between 1989 and 2017 which led to interruption of onchocerciasis transmission in 2018. This study investigated possibility of recrudescence of onchocerciasis with the hypothesis that insecurity-induced migration could cause recrudescence of onchocerciasis.<br>Methodology/Principal Findings<br>Six out of the 23 Local Government Areas (LGAs) in Kaduna State were selected for evaluation. Adult S. damnosum s.l. were captured across seven breeding sites using human landing collectors from July to October 2023. Pooled screen assays of 72 pools heads of black flies were conducted using quantitative polymerase chain reaction (qPCR) for O. volvulus detection. Dried blood samples from 3107 children aged 5–9 years were collected; with 1502 samples analysed using alkaline phosphatase enzyme-linked immunosorbent assay due to financial constraints. Twenty pools of the 72 pools of heads (27.8%) of S. damnosum s.l. analysed were positive for O. volvulus in Kagarko and Kachia LGAs at 95% UCL 0.49 infectivity (> 1/2000 infective flies) with overall prevalence of 0.32. Two children from security compromised communities tested seropositive (prevalence 0.31%; 95% UCL 0.317).<br>Conclusion/Significance<br>The findings demonstrated ongoing onchocerciasis transmission in Kaduna State despite the interruption in 2018. This calls for programmatic evaluation of the extent of recrudescence and identification of key drivers such as human migration, fly movement and insecurity.
**Additional Information:**	
**Question**	**Response**
**Government Employee**<br><br>Are you or any of the contributing authors an employee of the United States government?<br><br>Manuscripts authored by one or more US Government employees are not	No - No authors are employees of the U.S. government.

copyrighted, but are licensed under a CC0 Public Domain Dedication, which allows unlimited distribution and reuse of the article for any lawful purpose. This is a legal requirement for US Government employees.<br><br>This will be typeset if the manuscript is accepted for publication.	
**Financial Disclosure**<br><br>Enter a financial disclosure statement that describes the sources of funding for the work included in this submission. Review the submission guidelines for detailed requirements. View published research articles from *PLOS Neglected Tropical Diseases* for specific examples.<br><br>This statement is required for submission and **will appear in the published article** if the submission is accepted. Please make sure it is accurate.<br><br>**Funded studies**<br>Enter a statement with the following details:<br>• Initials of the authors who received each award<br>• Grant numbers awarded to each author<br>• The full name of each funder<br>• URL of each funder website<br>• Did the sponsors or funders play any role in the study design, data collection and analysis, decision to publish, or preparation of the manuscript?<br><br>Did you receive funding for this work?	Yes
Please add funding details.<br>as follow-up to "**Financial Disclosure**<br><br>Enter a financial disclosure statement that describes the sources of funding for the work included in this submission. Review the submission guidelines for detailed requirements. View published research articles from *PLOS Neglected Tropical Diseases* for specific examples.	This work received financial support from the Coalition for Operation Research on Neglected Tropical Diseases (COR-NTD), which is funded at The Task Force for Global Health primarily by the Bill and Melinda Gates Foundation and the United States Agency for International Development through its Neglected Tropical Diseases Program. The grant was administered by the African Research Network for Neglected Tropical Diseases under Small Grant Program-VI (SGP159/050.2023) to TOO. The funders had no role in study design, data collection and analysis, decision to publish, or preparation of the manuscript.<br><br>https://www.gatesfoundation.org/

Powered by Editorial Manager® and ProduXion Manager® from Aries Systems Corporation

This statement is required for submission and **will appear in the published article** if the submission is accepted. Please make sure it is accurate.<br><br>**Funded studies**<br>Enter a statement with the following details:<br>• Initials of the authors who received each award<br>• Grant numbers awarded to each author<br>• The full name of each funder<br>• URL of each funder website<br>• Did the sponsors or funders play any role in the study design, data collection and analysis, decision to publish, or preparation of the manuscript?<br><br>Did you receive funding for this work?"	https://www.usaid.gov/
Please select the country of your main research funder (please select carefully as in some cases this is used in fee calculation).<br>as follow-up to "**Financial Disclosure**<br><br>Enter a financial disclosure statement that describes the sources of funding for the work included in this submission. Review the submission guidelines for detailed requirements. View published research articles from *PLOS Neglected Tropical Diseases* for specific examples.<br><br>This statement is required for submission and **will appear in the published article** if the submission is accepted. Please make sure it is accurate.	UNITED STATES - US

**Funded studies**<br>Enter a statement with the following details:<br>• Initials of the authors who received each award<br>• Grant numbers awarded to each author<br>• The full name of each funder<br>• URL of each funder website<br>• Did the sponsors or funders play any role in the study design, data collection and analysis, decision to publish, or preparation of the manuscript?<br><br>Did you receive funding for this work?"	
**Competing Interests**<br><br>On behalf of all authors, disclose any competing interests that could be perceived to bias this work.<br><br>This statement will be typeset if the manuscript is accepted for publication.<br><br>Review the instructions link below and PLOS NTDs' competing interests policy to determine what information must be disclosed at submission.	The authors declare no conflict of interest during this study.
**Data Availability**<br><br>Provide a **Data Availability Statement** in the box below. This statement should detail where the data used in this submission can be accessed. This statement will be typeset if the manuscript is accepted for publication.<br><br>Before publication, authors are required to make all data underlying their findings fully available, without restriction. Review our PLOS Data Policy page for detailed information on this policy. Instructions for writing your Data Availability statement can be accessed via the Instructions link below.	All relevant data are within the manuscript and its Supporting Information files.

Powered by Editorial Manager® and ProduXion Manager® from Aries Systems Corporation

Manuscript Click here to access/download;Manuscript;Manuscript.docx

Recrudescence of transmission of onchocerciasis in some endemic communities in Kaduna State, Nigeria: What is the way forward?

Timothy O. Olanrewaju[1*], Felicia N. C. Enwezor[1], , Luret A. Lar[2], Michael A. Igbe[3], Ramatu A. Abdullahi[1],Monsuru A. Adeleke[4], Oluwatosin B. Adekeye[5], Elizabeth O. Elhassan[6]

[1]Nigerian Institute for Trypanosomiasis Research (*and Onchocerciasis*) Research, Kaduna, State, Nigeria

[2]Department of Community Medicine, Faculty of Clinical Sciences, College of Health Sciences, University of Jos, Plateau State, Nigeria

[3]Federal Ministry of Health, Federal Capital Territory, Abuja, Nigeria

[4]Department of Zoology, Faculty of Basic and Applied Sciences, Osun State University, Osogbo, Osun State, Nigeria

[5]Department of Psychiatry, Ahmadu Bello University Teaching Hospital Shika, Zaria, Kaduna State, Nigeria

[6]1A Mohammed Yusuf Lere Drive, Off Tafawa Balewa Way, Ungwan Rimi, Kaduna State, Nigeria

Corresponding author: timothyseye@gmail.com (TOO)

Abstract

Background

Onchocerciasis caused by the filarial parasite *Onchocerca volvulus* and transmitted by *Simulium damnosum* s.l. remains a public health concern in Nigeria. Infestation of *S. damnosum s.l.* along rivers Gurara and Kaduna; and heavy intensity of *O. volvulus* infection in Kaduna were documented in 1956. Control of onchocerciasis in Kaduna started 1954 with larviciding using dichlorodiphenyltrichloroethane which reduced *S. damnosum s.l.* population by 94% in 1966. Diethylcarbamazine was also used for human treatment but discontinued. Kaduna State Ministry of Health and its partners built on this achievement; used annual mass administration of ivermectin consistently between 1989 and 2017 which led to interruption of onchocerciasis transmission in 2018. This study investigated possibility of recrudescence of onchocerciasis with the hypothesis that insecurity-induced migration could cause recrudescence of onchocerciasis.

Methodology/Principal Findings

Six out of the 23 Local Government Areas (LGAs) in Kaduna State were selected for evaluation. Adult *S. damnosum* s.l. were captured across seven breeding sites using human landing collectors from July to October 2023. Pooled screen assays of 72 pools heads of black flies were conducted using quantitative polymerase chain reaction (qPCR) for *O. volvulus* detection. Dried blood samples from 3107 children aged 5–9 years were collected; with 1502 samples analysed using alkaline phosphatase enzyme-linked immunosorbent assay due to financial constraints. Twenty pools of the 72 pools of heads (27.8%) of *S. damnosum s.l.* analysed were positive for *O.*

volvulus in Kagarko and Kachia LGAs at 95% UCL 0.49 infectivity (> 1/2000 infective flies) with overall prevalence of 0.32. Two children from security compromised communities tested seropositive (prevalence 0.31%; 95% UCL 0.317).

Conclusion/Significance

The findings demonstrated ongoing onchocerciasis transmission in Kaduna State despite the interruption in 2018. This calls for programmatic evaluation of the extent of recrudescence and identification of key drivers such as human migration, fly movement and insecurity.

Keywords: Onchocerciasis, Kaduna State, recrudescence, *Simulium damnosum s.l.*, ivermectin, *Onchocerca volvulus*

Author summary

Onchocerciasis is a disease that affects humans and is transmitted by bites of *Onchocerca volvulus*infected black flies. Nigeria accounts for the highest onchocercal blindness in sub-Saharan Africa. Collaborative effort by various stakeholders in Kaduna State, Nigeria yielded remarkable achievement of successful interruption of onchocerciasis transmission in year 2018 through annual mass administration of medicine (MAM). However, our study established the ongoing transmission of onchocerciasis in some areas of Kaduna State after four years stop MAM decision. Kaduna State Government, partners and stakeholders should urgently take actions to mitigate the ongoing transmission of onchocerciasis in the State to safeguard the achievements attained after years of MAM. We recommend the following; assessment of entire Kaduna State to determine the extent of the spread of the recrudescence; in-depth analysis of

annual therapeutic coverage rates; community-driven slash and clear approach for vector control; review of the monitoring and evaluation system across all levels; using Esperanza Window Trap at black flies breeding sites to complement the slash and clear approach; modelling the approaches to determine the most appropriate frequency of treatment with ivermectin.

Introduction

Human infection with the filarial parasite *Onchocerca volvulus* causes onchocerciasis (also known as river blindness). *Onchocerca volvulus* is transmitted to humans by bites of *Simulium damnosum s.l.* (black flies) in Nigeria. There are nine sibling species within the *S. damnosum* complex that can transmit onchocerciasis [1, 2]. Onchocerciasis is one of the Neglected Tropical Diseases (NTDs) with high public health impact in Africa. Over 99% of all cases of onchocerciasis and onchocercal-related blindness are found in Africa; with Nigeria having about 40% of the global population at risk [3]. Onchocercal blindness ranked second leading cause of preventable blindness in the world after trachoma [4]. The disease is peculiar to the poor with economic implications resulting in abandonment of rich fertile farmlands which resulted in food insecurity and poor school attendance, sometimes forcing children to drop out of school to assist blind parents/guardians. Other manifestations include; skin disfiguration and stigmatization. These impact on the wellbeing of individuals with the disease become a major challenge to the achievement of Sustainable Development Goals (SDGs); “End hunger, achieve food security and improved nutrition and promote sustainable agriculture”(SDG 2) and “Ensure healthy lives and promote well-being for all at all ages (SDG3) as documented by Engels [5].

Crosskey [6] documented that adult *S. damnosum s.l.* dispersal ability is estimated to be 234,000 Km^2 (90,000 square miles) in Northern Nigeria. Nearly half of the areas infested by *S. damnosum s.l.* in Northern Nigeria lies within region of rivers Gurara and Kaduna which is about 37,400 Km^2 (14,400 square miles). The control of onchocerciasis in Kaduna State started in 1954 with larviciding using dichlorodiphenyltrichloroethane (DDT) along rivers Gurara and Kaduna [7, 8]. By 1966, this control effort had achieved 94% reduction in *S. damnosum s.l.* population density. Diethylcarbamazine was also used for human treatment but discontinued due to high toxicity. The State started annual MAM of ivermectin in 1989. Tekle et al. [9] reported that 17years of consistent annual administration of ivermectin could potentially interrupt transmission of onchocerciasis in two foci (Birnin Gwari and Kauru/Lere) within Kaduna State. By 2018, interruption of transmission of the disease was attained and MAM implementation was stopped [10].

However, the current security challenges which revolve around ethno-religious and farmer-herder conflicts over land access heighten violence in Kaduna State [11]; migration to the State from the border States like Niger, Kano and Federal Capital Tertiary (FCT) Abuja where transmission of onchocerciasis is still ongoing [12] and vector migration due to climate change could cause recrudescence of onchocerciasis in Kaduna State. According to the International Organization for Migration report [13], Kaduna State has a minimum of 117,880 internally displaced persons (IDPs) as a result of banditry and kidnapping (45%), communal clashes (29%), natural disaster (13%), farmers-herders clashes (12%), and insurgency (2%).Our interaction with the State Emergency Management Agency (SEMA) revealed that the only State government managed IDP camp is located at Giwa Local Government Area (LGA). However, we identified a

faith-based IDP camp at Agwan Zawu, Gonin-Gora, Chikun LGA and another at Evangelical Church Winning All (ECWA), Ungwan Musa, Zonkwa, Zangon-Kataf LGA. Interruption of transmission of onchocerciasis in Kaduna State might be difficult to sustain because of high number of IDPs within the State without any organized or governmental camp. Also, increase in inter-state migration within northwest region of Nigeria and climate change could affect transmission of onchocerciasis. These challenges could jeopardise efforts towards 2030 target of elimination of onchocerciasis, other targets in the NTD Roadmap 2021-2023 and SDG 2 and 3.

We investigated possible recrudescence of onchocerciasis transmission in six LGAs in Kaduna State in the face of insecurity and human migration four years after interruption of transmission.

Methods

Study area

Kaduna State lies between 10°36’33” N and 7°25’46” E and shares boundaries with Kano and Katsina States in the north; Plateau State to the east; Nasarawa State to the south; Niger State in the west and the FCT Abuja to the southwest. Some of the major rivers in the State include river Gurara and river Kaduna which serve as good breeding sites for black flies [14]. All of the States that share boundaries with Kaduna are currently treating with ivermectin except Plateau and Nasarawa States, which have interrupted transmission, and Katsina State which was hypo endemic for onchocerciasis and was ineligible for treatment requires onchocerciasis elimination mapping (OEM). Kaduna State consists of 23 Local Government Areas (LGAs) with a projected

population of more than six million people and estimated land area of about 46,063 Km^2. The demographic vegetation of the State is mainly savannah grassland with few areas of forest. This study was carried out in six LGAs of Kaduna State, namely Chikun, Kachia, Kaduna North, Kaduna South, Kagarko and Zangon-Kataf (Fig. 1). Kaduna North and Kaduna South LGAs were selected due to the presence of black fly breeding sites at river Kaduna that runs through the two LGAs and influx of migrants from Chikun LGA. Chikun, Kachia and Kagarko LGAs were included because of the presence of black flies breeding sites along riversGurara and Kaduna; insecurity and presence of migrants. Zangon-Kataf LGA was chosen based on high level of insecurity and proximity to breeding site in Kachia. Seven breeding sites across five LGAs serve as catching points of black flies namely Kabala Doki (Kaduna North LGA), Kwata (Kaduna South LGA), Tsallake (Chikun LGA), Atara and Dogon-Daji (Kagarko LGA), Amuse and Gurara (Kachia LGA) as shown in Table 1.

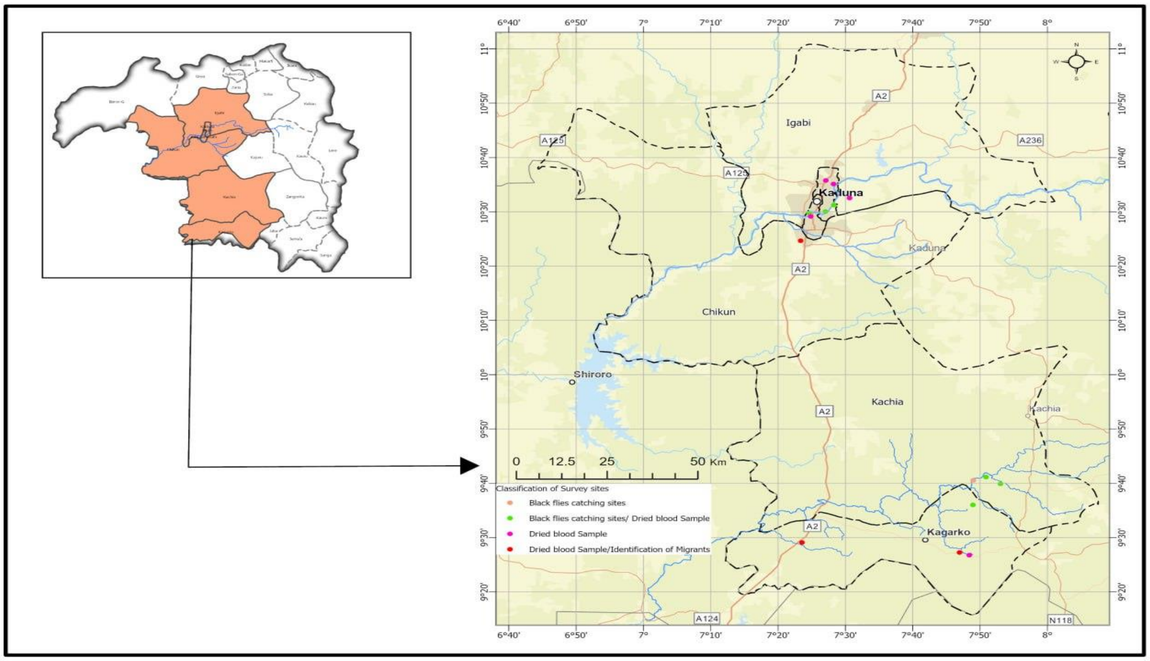

Fig 1: Map of Kaduna State showing the study locations (ArcGIS Pro Software version 3.2.0)

Epidemiological evaluation

The epidemiological study was carried out in all the selected LGAs. Sample collection was done between July and October, 2023.

Study population

Farming is the major occupation of citizens at Chikun, Kagarko, Kachia and Zangon-Kataf, but for Kaduna South and Kaduna North LGAs majority are civil servants and business men/women. Dried blood samples were collected from children (male and female) between 5 and 9 years old across the study areas.

Sample size and sample collection

Dried blood samples (DBS) were collected from 3107 children aged 5-9 years from various communities and primary schools across the six LGAs using World Health Organization (WHO) guidelines for epidemiological assessment of onchocerciasis transmission in children [15, 16]. The sample from each community was estimated proportionally to available children within the required age group. Blood samples were collected from all assented children aged 5-9 years through finger-prick using sterile lancet after swabbing the finger with methylated spirit; a lancet to each child. About 100 µL of blood from each child was spotted on Whatman number 1 filter paper (Whatman International Ltd Maldstone, England); using one filter paper for a child. Each of the filter papers was fixed to a Styrofoam using pencil for air-drying. For each community, each child's sample was stored in a small ziplock bag and then put into a larger ziplock bag containing silica gel (Desicare, Reno, NV, USA), labeled appropriately and store at 25°C.

Sample analysis

The samples were analysed within maximum of six months of sample collection at Osun State University Multi-Disciplinary Research Laboratory, Osogbo, Nigeria using AP-ELISA technique as detailed in S1 Methods

Ethical approval

The study protocol was approved by the Health Research Ethics Committee (HREC) of Kaduna State Ministry of Health (Ref No. NHREC/17/03/2018). Verbal assent was obtained from each child with consent of either parent or guardian of the child before blood sample collection. Volunteers that acted as human attractants for black fly catching were told about the personal risks and community benefits of participation and given the option to opt out of participation at any time without any repercussions after signing the participant consent form. They were offered ivermectin at the end of the study according to specifications.

Entomological evaluation

Seven known breeding sites of *S. damnosum s.l.* in five LGAs – Chikun, Kachia, Kaduna North, Kaduna South and Kagarko were assessed to ascertain if they were currently breeding the vector. The coordinates of the breeding sites were taken using Garmin™ geographical positioning information system (GPIS) machine. Based on the outcome of the assessments, community leaders of first-line communities (communities within 20km to *S. damnosum s.l.* breeding site) and second-line communities (communities within 20km – 40km away *S. damnosum s.l.* breeding site) were sensitized on the importance of preventing blindness due to onchocerciasis. The leaders identified two persons from their communities that serve as human

landing collectors (HLCs) for *S. damnosum s.l.* The HLCs were clustered into two groups for a two days theory and practical training on capturing of black flies and recording as described [17, 18]. Black fly capturing activities were conducted between July and October 2023 being the peak of black fly breeding season. Two HLCs worked in an alternate arrangement hourly for two days per week between 07:00 h and 18:00 h. The flies collected were stored in preservative bottles containing isopropyl alcohol. The bottles were labelled accordingly for each catching site. At the end of capturing of the flies, the study entomologist sorted all the flies and confirms the flies that were *S. damnosum s.l.* using morphological characteristics such as conspicuous compound eyes, short horn-like antennae, hump-backed at midsection of the thorax and broad, colourless and transparent wings. The black flies were stored in isopropyl alcohol and hand carried to the Multi-disciplinary Research Laboratory, Osogbo, Nigeria to determine the *O. volvulus* infectivity rate in black flies using real-time qPCR technique assay with a mitochondrial DNA target as detailed in S2 Methods. The separation of the heads from the bodies, extraction and qPCR were carried out as described [19].

Statistical Analysis

The number of black fly collected in each location was normalized and then subjected to One - way Analysis of Variance using SPSS version 23.0 to determine the significant difference in fly variation across catching sites and also the months of collection. The Ov16 antibody prevalence in children was determined using 95% confidence intervals of > 0.1% upper confidence limit (UCL) as described [20] using the formula:

95% Confidence interval = proportion positive (p) ± 1.96($\frac{\sqrt{P(1-\mathrm{P})}}{\mathrm{n}}$). The poolscreen software 2.1 was used to determine *O. volvulus* infectivity rate in pools of black flies at 95% confidence interval.

Results

Out of the 1,502 DBS of children aged 5 – 9 years; two children from Kagarko LGA were Ov16 sero-positive with prevalence of 0.13% (95% UCL 0.317) as shown in Tables 1. These children were from first-line and second-line communities, black fly breeding sites; and communities with IDPs and migrants. The two Ov16 sero-positive samples were from Atara community (first-line community) and Kubacha community (second-line community) within Kagarko LGA. Six thousand eight hundred and ninety-seven (6,897) flies out of 7,782 reported flies from the field were identified as *S. damnosum s.l.* (Table 2).The highest number 2,858 (71.48%) of black flies captured was from Kuratam community followed by 2,072 (30.04%) in Atara within Kagarko LGA. Gurara and Amuse communities had 1,181 (17.12%) and 782 (11.33%) respectively in Kachia LGA. Only four black flies were captured along river Kaduna; two at Kabala and two at Kinkinau. We observed a significant variation in the number of flies captured at various catching sites during the wet season as the number of black fly collected at Kuratam, Atara, Gurara and Amuse were significantly higher (*p>0.05*) than those collected in Kabala, Kinkinau and Babban Saura (Table 2). There was significant variation (*p>0.05*) in the number of flies collected during the four months catching period (Table 3).

Twenty pools of the 72 pools of heads (27.8%) of *S. damnosum s.l.* analysed were positive for *O. volvulus* at 95% UCL 0.49 of black flies infectivity (> 1/2000 infective flies) with overall prevalence of 0.32 (Table 4). Four out of the seven black flies catching sites (57.14%) tested

positive with both sites in Kagarko and Kachia LGAs testing positive. We observed the highest infectivity rate (33%) in black flies captured at Amuse catching site in Kachia LGA.

Table 1: Dried blood sample Ov16 antibody/antigen test using AP-enzyme linked immunosorbent assay

Local government area	Community	Proximity to black fly breeding site	Number of children sampled	No. of DBS analysed	No. of positive DBS
**Kaduna North**	Kabala	First line	304	86	0
	Angwan-Gwari	Second line	136	136	0
	Rafin-Guza	Second line	160	100	0
	Kawo	Second line	106	0	0
**Kaduna South**	Kinkunu	First line	176	100	0
	Sheilk Gumi Primary School	Second line	233	0	0
	Down Quarters	Second line	136	135	0
	Television	Second line	134	0	0
	Aliyu Makarma Primary School	Second line	276	0	0
**Chikun**	Angwan Zawu IDP camp	Second line	23	23	0
	Doka Mai Jama'	First line	137	137	0
	UBE Bagado Primary School	Second line	122	0	0
	Babban Saura	First line	237	120	0
**Kagarko**	Kuratam	First line	231	231	0
	Atara	First line	76	76	1
	Kubacha	Second line	100	100	1
	Dogo Kurumi	Second line	71	71	0
**Kachia**	Gurara	First line	49	49	0
	Zonkwa Primary School	Second line	262	0	0
**ZangonKataf**	Samaru Kataf	Second line	138	138	0
**TOTAL**			**3107**	**1,502**	**2**
	**Lower and Upper Limit CI (95%)**				**-0.051 - 0.368**

The 95% UCL of OV-16 positivity > 0.1% indicates ongoing transmission of Onchocerciasis in Kaduna State

Table 2: Human landing capturing of flies at various breeding sites in the study areas

Local Government Area	Community	Catching Site	Number of fly catchers	Number of flies captured in the field	Number of confirmed black flies in the laboratory	Number of pool	Black fly population density in wet season (%)	Mean + SD ($p < 0.05$)
**Kaduna North**	Kabala	Kabala	2	148	2	1	1.4	37.00+8.25[a]
**Kaduna South**	Kinkinau	Kwata	2	27	2	1	7.4	6.75+3.86[a]
**Chikun**	Babban Saura	Tsallake	2	11	0	0	0	2.75+1.89[a]
	Atara	Atara	2	2233	2072	21	92.8	558.25+148.30[b]
**Kagarko**	Kuratam	Dogon Daji	2	2924	2858	29	97.7	731.00+70.77[c]
	Amuse	Gurara	2	1175	782	8	66.6	293.75+113.72[d]
**Kachia**	Gurara	Gurara	2	1264	1181	12	93.4	316.00+8.36[d]
	**TOTAL**	**7**	**14**	**7,782**	**6,897**	**72**		

The mean ± SD (standard deviation) with different superscript along the same column indicates statistically significant difference ($p < 0.05$).

Table 3: Variation in fly population density during wet season

Local Government Area	Community	July	August	September	October
**Kaduna North**	Kabala Doki	30	42	46	30
**Kaduna South**	Kinkinau	12	7	5	3
**Chikun**	Babban Saura	0	4	3	4
**Kagarko**	Atara	587	619	683	344
	Kuratam	650	760	813	701
**Kachia**	Amuse	387	349	309	130
	Gurara	318	333	293	320
	**Total**	**1984 (25.5%)**	**2114 (27.2%)**	**2152 (27.7%)**	**1532 (19.70%)**

Table 4: Detection of *Onchocerca volvulus* DNA signal in *S. damnosum s.l.* pools using qPCR

Local government area	Community	No. of black flies	No. of pools of	No. of pools <	No. of positive	Prevalence by Ov ND5 qPCR (95% confidence

		analysed	100	100	pools	interval)
**Kaduna North**	Kabala Doki	2	0	1(2)	0	0.00 (0.00)
**Kaduna South**	Kinkinau	2	0	1(2)	0	0.00 (0.00)
**Chikun**	Tsalake	0	0	0	0	0.00 (0.00)
**Kagarko**	Kuratam	2,858	28	1(58)	7(24%)	0.0275 (0.13-0.545)
	Atara	2,072	20	1(72)	7(33%)	0.044 (0.19 – 0.79)
**Kachia**	Gurara	1,181	11	1(81)	3(25%)	0.0287 (0.09-0.76)
	Amuse	782	7	1(82)	3(38%)	0.047 (0.15-1.18)
**TOTAL**		**6,897**	**66**	**6**	**20**	**0.32 (0.21-0.49)**

The 95% UCL of black flies infectivity of >1/2,000 infective flies/2,000 indicates ongoing transmission. CT-values for pools of black flies are detailed in S1 Results

Discussion

Black flies prefer breeding in conducive river systems where the physico-chemical parameters of the rivers support their establishment [7]. The results of the present study showed that the river system in Kaduna indeed support the breeding of the black flies, though with variation as evident in the current study. Historical data showed that Davies et al. [7] carried out larviciding using DDT along river Gurara from Suleja, Abuja to Tafa due to the high biting density of black fly along the river. This resulted to 94% reduction in the population density of *S. damnosum* in the area. On the one hand, the high number of flies collected along river Gurara still suggests that breeding of black fly continues along the river. On the other hand, the high water level in river Kaduna resulted in low density of black fly in Chikun, Kaduna North and Kaduna South LGAs despite a four months attempt to capture black flies. The low density of black fly could be attributed to flooding, inadequate rocky basements and low velocity of water. According to Opara et al [21] and Eyo et al [22] heavy rainfall and stormy weather play major role in washing away most black flies breeding sites during the rainy season. Inadequate rapids and rocky basements which form favourable breeding sites for black flies [23] might also affect the population density. Furthermore, high water level during heavy rain can slow down the velocity

of water which reduces the favourable conditions for breeding of *Simulium* species [24]. Though the breeding sites along river Gurara were probably not visited during cytotaxonomic identification of members of *S. damnosum* complex [25], breeding of black fly and transmission of onchocerciasis along the river at Gantang in Kagarko LGA has been documented [14]. The high population density of black fly recorded in Amuse, Gurara, Atara and Kuratam sites could be as a result of vegetation, rocks, speed of the water and other favourable physico-chemical parameters of the river Gurara [26, 27]. The population density along river Gurara corroborates the earlier report [14]. The high infectivity rate in black flies we observed in Amuse, Atara, Gurara and Kuratam suggests that transmission cycle of onchocerciasis has been sustained for awhile in Kachia and Kagarko LGAs. This contradicts the previous documentation [10] that no transmission of *O. volvulus* in Kaduna State; thus recrudescence of onchocerciasis established in the State. There is possibility of imported infection from the neighbouring Niger State and FCT Abuja to Kaduna State. According to Crosskey [6], rivers Gurara, Kaduna, Sarkin Pawa (in Niger State) are always infested with black fly with rivers Gurara and Kaduna forming major fly area of approximately 140 miles (225 km). The ability of *S. damnosum* complex to migrate >400 km away in large numbers [1, 28, 29] due to seasonal variation can threaten the interruption of transmission of onchocerciasis and elimination of the disease in Kaduna State.

To curb the black fly population density, slash and clear approach in Kaduna and the neighbouring endemic areas can be adopted. This approach requires removal of vegetation from breeding sites (fast flowing well oxygenated sediment free water) along rivers to disrupt *S. damnosum* breeding activities. Smith et al. [30] recommended slash and clear using community-driven implementation strategy at least once a year for accelerating achievement

of onchocerciasis elimination. Slash and clear vector control using community-driven implementation strategy at Maridi dam spillway in South Sudan reduced *S. damnosum* by >90% six months post intervention and <50% twelve months post intervention [31]. The approach has also been used in Uganda with 97% reduction in biting rate after single intervention during early wet season [32] and Cameroon with 32.9% reduction in black fly population density [33]. Combination of community directed treatment with ivermectin (CDTi) with slash and clear vector control will accelerate achievement of onchocerciasis in Africa. The use of Esperanza Window Trap can also be deployed along black flies breeding sites to complement the slash and clear approach as described [34].

Despite the on-going treatment with ivemectin for Lymphatic filariasis in Kachia and Kagarko LGAs after stopping treatment for onchocerciasis in 2018, the 0.13% Ov16 serological test result in children aged 5-9 years coupled with overall infectivity rate of 0.32 (C.I. 0.21-0.49) which is above the WHO threshold of <1/2000 infective rate in black flies) indicates ongoing transmission of onchocerciasis in the study area. This implies that there is recrudescence of onchocerciasis in Kaduna State despite the interruption of transmission of onchocerciasis recorded in year 2018. The reasons for these observations may be attributed to insecurity, human migration and diagnostic technique used. The WHO onchocerciasis technical advisory subgroup in its sixth meeting [35] observed that real time qPCR assay used in this study has a higher sensitive than O-150 PCR ELISA and could detect residual transmission in fly pools negative for O-150 PCR ELISA. Furthermore, in a recent publication by Adeleke et al [34] on a comparative study of OvND5 qPCR and O-150 PCR ELISA in three ecological zones in Nigeria, they reported that OvND5 qPCR is more sensitive. More black fly pools were positive for *O.*

volvulus by OvND5 qPCR compared with O-150 PCR in derived savannah (31.15 vs. 15.57%), montane forest (11.54 vs. 0%) and rainforest (23.08 vs. 2.56%).

Searching beyond the diagnostic technique used, the climate change with great impact on black flies' movement and human migration (intra and interstate) as a result of communal conflicts, political crisis, kidnapping and insurgency are also potential threats to elimination of onchocerciasis [37, 38, 39]. The earlier therapeutic coverage of 65% with ivermectin attained in Kaduna State before stopping MAM [10] might be insufficient to break the transmission cycle considering the high infectivity rate of black flies in some part of the State.

Kagarko, Kachia and other LGAs in southern part of Kaduna State have had several episodes of insecurity since 2011 [40]. During the period of insecurity, the population in the affected communities moved out to other LGAs within the State and Niger State then returned when the situation is calm. The migration of people from affected communities poses a threat to elimination of onchocerciasis in other LGAs that share boundaries with the LGAs in Kaduna; Tafa and Suleja in Niger States and Bwari in FCT, Abuja. This calls for a review of geographic and therapeutic coverage rates, implementation of community self monitoring and intensify monitoring and evaluation of treatment. There is need for development of model that will determine appropriate frequency of treatment for Kaduna State. Adoption of biannual treatment in River Gambia, Senegal (April or May and October or November) for more than 10 years has been successfully implemented with desired outcomes [41]. Also, the biannual community directed treatment with ivermectin resulted to elimination of onchocerciasis in Colombia in 2013 [42]; Ecuador in 2014 [43] and Guatemala in 2016 [44]. To achieve

elimination of onchocerciasis, Mexico changed frequency of treatment with ivermectin to quarterly and achieved elimination in year 2015 [45].

Limitations of the study

The limitations of the study include inability to analyse the 3107 dried blood samples (DBS) collected from children aged 5-7years because of limited resources, devaluation of the naira, inflation and high cost of reagents during this study. Other limitations were failure to collect DBS from children in Amuse; a first-line community with the highest infectivity rate in black flies due to insecurity during the period of the study and inability to collect black flies in the dry season as a result of the short duration of the study.

Conclusion

In conclusion, the high *O. volvulus* infectivity rate (above WHO threshold) in black flies and positive samples of DBS in children in some of the communities sampled showed that there is an ongoing transmission in the study area. This, by implication established the recrudescence of the disease after interruption of the disease was achieved in Kaduna in 2018. This calls for urgent programmatic evaluation of the extent of recrudescence (since our study only covered the southern Kaduna) and the key drivers of the recrudescence. Does the recrudescence being driven by socio-cultural, human migration, fly movement or large scale of insecurity are issues awaiting elucidation through further studies.

Supporting information

S1 Methods: Detailed protocol for Ov16 ELISA

S2 Methods: Detailed protocol for DNA isolation from black fly heads and qPCR

S1 Results: Detailed CT-values for pools of black flies examined

Author contributions

Conceptualization: Felicia N. C. Enwezor, Timothy O. Olanrewaju, Elizabeth O. Elhassan

Data Curation: Timothy O. Olanrewaju, Felicia N. C. Enwezor, Oluwatosin B. Adekeye

Methodology: Timothy O. Olanrewaju, Felicia N. C. Enwezor, Michael Igbe

Project Administration: Timothy O. Olanrewaju, Felicia N. C. Enwezor

Funding Acquisition: Timothy O. Olanrewaju, Felicia N. C. Enwezor, Elizabeth O. Elhassan, Luret A. Lar, Oluwatosin B. Adekeye

Investigation: Timothy O. Olanrewaju, Felicia N. C. Enwezor, Michael Igbe, Ramatu A. Abdullahi

Formal Analysis: Monsuru A. Adeleke, Michael Igbe

Supervision: Felicia N. C. Enwezor, Elizabeth O. Elhassan, Luret A. Lar

Validation: Monsuru A. Adeleke, Luret A. Lar, Felicia N. C. Enwezor, Oluwatosin B. Adekeye

Writing-Original Draft: Timothy O. Olanrewaju, Michael Igbe, Luret A. Lar

Writing-Review and Editing: Timothy O. Olanrewaju, Felicia N. C. Enwezor, Luret A. Lar, Ramatu A. Abdullahi, Micheal Igbe, Monsuru A. Adeleke, Oluwatosin B. Adekeye, Elizabeth O. Elhassan.

Funding

This work received financial support from the Coalition for Operation Research on Neglected Tropical Diseases (COR-NTD), which is funded at The Task Force for Global Health primarily by the Bill and Melinda Gates Foundation and the United States Agency for International Development through its Neglected Tropical Diseases Program. The grant (SGP159/050.2023) was administered by the African Research Network for Neglected Tropical Diseases (ARNTD). The funders had no role in study design, data collection and analysis, decision to publish, or preparation of the manuscript.

Acknowledgment

We acknowledge Sightsavers, Kaduna Office and Kaduna State Ministry of Health for availing us essential information during the design of the study protocols and sample collection. Also, Nigerian Institute for Trypanosomiasis Research, Kaduna for the provision of the vehicle used during field activities. Osun State University Multi-disciplinary Research Laboratory, Osogbo, Nigeria provided additional reagents for Ov16 serological test and qPCR analysis of *S. damnosum s.l.*

Conflict of Interest

The authors declare no conflict of interest during this study.

References

1. Post KJ, Onyenwe E, Somiari SA, Mafuyai HB, Crainey JL, Ubachakwu PO. A guide to the *Simulium damnosum* complex (Diptera: Simuliidae) in Nigeria, with a cyto taxonomic key for the identification of the sibling species. *Ann. Trop. Med. Parasitol*. 2011 Jun 1; 105(4):277-297. https://doi.org/10.1179/136485911x12987676649700PMID: 21871165
2. Chikekie FM, Opara KN, Ubulom PME, Yaro CA, Al-Akeel RK, Osei-Atweneboana MY et al. Onchocerciasis transmission status in some endemic communities of mass drug

administration with ivermectin. *Sci Rep*. 2023; 13:5413 doi: 10.1038/s41598-023-31446-6PMID: 37012274

3. World Health Organization. Ending the neglect to attain the Sustainable Development Goals: A roadmap for neglected tropical diseases 2021–2030. Available: https://apps.who.int/iris/rest/bitstreams/1326801/retrieve [Accessed June 15, 2023].
4. World Health Organization. Onchocerciasis: Fact sheet 2022. https://www.who.int/news-room/fact [Accessed September 12, 2023]
5. Engels D. Neglected tropical diseases in the Sustainable Development Goals. *The Lancet* 2016 January 16; 387(10015), 223-224. https://doi:1016/s104-6736(16)00043-x
6. Crosskey RW. The distribution of *Simulium damnosum* Theobald in northern Nigeria. *Trans. Royal Society Trop. Med. Hyg*. 1956; 50: 379–392
7. Davies JB. The *Simulium* control scheme at Abuja, Northern Nigeria and its effect on the prevalence of onchocerciasis in the area. *Bull. World Health Org*. 1968; 39:187-207
8. Bump JB. The Crosskey-Davies Experiment and Onchocerciasis Control in West Africa. *PLoS Negl Trop Dis* 2014 October 9; 8(10): e3223. https://doi:10.1371/journal.pntd.0003223
9. Tekle AH, Elhassan E, Isiyaku S, Amazigo UV, Bush S, et al. Impact of long-term treatment of onchocerciasis with ivermectin in Kaduna State, Nigeria: first evidence of the potential for elimination in the operational area of the African Programme for Onchocerciasis Control. *Parasit Vectors* 2012 Feb 7; 5:28. https://doi:10.1186/1756-3305-5-28
10. Isiyaku S, Igbe M, Madaki S, Hamill LC, Ndongmo P, Adamani W, et al. The interruption of transmission of onchocerciasis in Kaduna, Kebbi and Zamfara State, Nigeria: another milestone achievement. *Intern. Health* 2022 May 12; 14(2):ii43-ii54. https://doi.org/10.1093/inthealth/ihac036
11. Olajumoke A. (2021). Confronting Nigeria's Kaduna crisis https://africacenter.org/spotlight/confronting-nigerias-kaduna-crisis [Accessed February 20, 2022].
12. Yahaya A, Kela SL, Suleiman MM, Sama'ila AB. Prevalence studies on Onchocerciaisis (River Blindness) in some villages from Three Senatorial Districts, Kano State, Nigeria. *South Asian Journal Parasitol.* 2021*;* 5(4):141-152; Article no. SAJP 80294
13. Displacement Tracking Matrix (DTM) of the International Organization forMigration (IOM). IOM Nigeria-North-central and North-west zones displacement report. 2023; Round 12 needs monitoring. Available: https://dtm.iom.int/Nigeria [Accessed May 8, 2024]
14. Isiyaku S, Adamani W, Louise H, Ndams IS, Ezekiel K, Kal, A, et al. Entomological assessment of the Impact of long-term mass administration of ivermectin in onchocerciasis-endemic communities in Kaduna State, Nigeria. [Preprint]. 2021 [cited 2022 August 21]. Available from: https://doi.org/10.21203/rs.3.rs-840823/v1

15. World Health Organization. Guidelines for Stopping Mass Drug Administration and Verifying Elimination of Human Onchocerciasis: Criteria and Procedures. *Geneva, Switzerland*: WHO 2016.[Google Scholar]
16. Nigerian Federal Ministry of Health. Nigeria Onchocerciasis elimination plan. *Abuja: Federal Ministry of Health*; 2017.
17. Service MW. Methods for sampling adult *Simuliidae,* with special reference to the *Simulium damnosum* complex*. Tropical Pest Bulletin* 1977 (5). Centre for Overseas Pest Research, London, UK; ISBN 0-85135-087-9 http://gala.gre.ac.uk/12219
18. Adeleke MA, Mafiana CF, Sam-Wobo SO, Olatunde GO, Ekpo UF, Akinwale OP et al. Biting behaviour of *Simulium damnosum* complex and *Onchocerca volvulus* infection along Osun River, Southwest Nigeria. *Parasit Vector 2010 October 7;* 3(93): 1-5. doi:10.1186/1756-3305-3-93 http://www.parasitesandvectors.com/content/3/1/93
19. Hendy A, Kruger A, Pfarr K, De Witte J, Kibweja A, Mwingira U, et al. The black fly vectors and transmission of *Onchocerca volvulus* in Mahenge, south eastern Tanzania. *Acta Trop.* 2018 February 2; 181:50–9. https://doi.org/10.1016/j.actatropica.2018.01.009
20. Richard FO, Katabarwa M, Bekele F, Tadesse Z, Mohammed A, Sauerbrey M, et al. Operational performance of the *Onchocerca volvulus* "OEPA" OV16 ELISA serological assay in mapping, stop mass drug administration and post treatment surveillance survey. *Am J Trop Med Hyg.*2018;99(3):747–752 doi: 10.4269/ajtmh.18-0341
21. Opara, K.N., Fagbemi, O.B., Ekwe, A., and Okenu, D.M (2005). Status of forest Onchocerciasis In the lower cross river basin Nigeria. Entomologic profile after five years of Ivermectin Intervention. *Am. J. Trop. Med. Hyg*73(2) pp 371-376.
22. Eyo, E.J., Ikechukwu, E.O., Ubachukwu, P.O., Ivoke, N., Ekeh, F.N (2014). Effects of climatic conditions on the biting density and relative abundance of *Simulium damnosum* complex in a rural Nigerian farm settlement. *Annals of Agricutural and Environmental Medicine vol 21 no 4 697-700*.
23. Nanjul GO, Chinedu OU, Job N, Meshach JS, Apollos WP, Adams C, et al. Oviposition and Cues in black flies: Possible clues for boom of adult population. *Inter. J. Mosquito Res.* 2017 February 17; 4(2):111-124. ISSN: 2348-5906
24. Ikpeama CA, Nwoke BEB, Anosike, JC, Orji, NM and Onyirioha CN. Studies on the biting behaviours female *Simulium damnosum* complex in some endemic areas of Imo State, Nigeria. *Inter. Wld. J. Sci. Tech.* 2007; 3:1-5
25. Mafuyai HB, Post RJ, Vajime CG, Molyneux DH. Cytotaxonomic identifications of the *Simulium damnosum* complex (Diptera: Simuliidae) from Nigeria. *Trop. Med Intern. Health* 1996; 1(6):779-785
26. Ibeh OO, Nwoke BEB, Adegoke JA. Distribution and ecology of breeding sites of *Simulium damnosum s.l*. in south–eastern primary health zone of Nigeria. Nigerian Journal of Parasitology 2007; 28:32–38.
27. Oforka LC, Adeleke MA, Anikwe JC, Makanjuola WA. Population fluctuations and effect of climatic factors on the relative abundance of *Simulium damnosum* complex (Diptera: Simuliidae). Environ. Entomol. 2019; 48: 284–290.

28. Walsh JF, Davies JB, Garms R. Further studies on the reinvasion of the onchocerciasis control programme by *Simulium damnosum s.l.*: the effects of an extension of control activities into southern Ivory Coast during 1979. Tropenmedizin und Parasitologie 1981; 32, 269–273.
29. Baker RHA, Guillet P, Se´ke´te´li A, Poudiougo P, Boakye D et al. Progress in controlling the reinvasion of windborne vectors into the western area of the Onchocerciasis Control Programme in West Africa. Philosophical Transactions of the Royal Society of London. 1990; Series B, 328, 731–750.
30. Smith ME, Bilal S, Lakwo TL, Habomugisha P, Tukahebwa E, ByamukamaE, et al. Accelerating river blindness elimination by supplementing MDA with a vegetation "slash and clear" vector control strategy: a data-driven modeling analysis. *Sci Rep*. 2019; 9:15274. https://doi:10.1038/s41598-019-51835-0
31. Raimon S, Lakwo TL, Sebit WJ, Siewe Fodjo JN, Alinda P, et al. "Slash and Clear", a Community- Based Vector Control Method to Reduce Onchocerciasis Transmission by *Simulium sirbanum* in Maridi, South Sudan: A Prospective Study. Pathogens 2021 October 15; 10, 1329. https://doi.org/10.3390/pathogens10101329
32. Jacob B, Loum D, Munu D, Lakwo T, Byamukama, E, Habomugisha P, et al. Optimization of Slash and Clear Community-Directed Control of *Simulium damnosum Sensu Stricto* in Northern Uganda. Am. J. Trop. Med. Hyg. 2021, 104, 1394–1403. https://doi:10.4269/ajtmh.20-1104
33. Domche A, Djeunga HCN, Nwane PB, Njitchouang GR, Fesuh BN, Njiokou F, et al. Significant reduction of black fly densities in persistent onchocerciasis area following pilot implementation of an environment friendly approach (slash and clear). *Sci. Reports* 2024; 14:408 https://doi.org/10.1038/s41598-023-50747-4
34. Adeleke MA, Opara KN, Mafuyai HB, Nwoke BEB, Surakat OA, Akinde SB, et al. Improving onchocerciasis elimination surveillance: trials of odour baited Esperanza Window Traps to collect black fly vectors and real-time qPCR detection of Onchocerca volvulus in black fly pools. *Parasit Vector* 2024 November 18, 17:471 https://doi.org/10.1186/s13071-024-06554-5
35. WHO Report of the Sixth meeting of the WHO onchocerciasis technical advisory subgroup: virtual meeting, 19 – 21 December 2022. Geneva: WHO; 2023
36. Doherty M, Grant JR, Pilotte N, Bennuru S, Fischer K, Fischer PU, et al. Optimized strategy for real-time qPCR detection of *Onchocerca volvulus* DNA in pooled *Simulium sp*. Black fly vectors. *PLoS Negl Trop Dis* 2023 17(12):e0011815. https://doi.org/10.1371/journal. pntd.00118 15
37. Coninx R. Tuberculosis in complex emergencies. *Bull World Health Organ.* 2007; 85:637–40.
38. Meteke S, Stefopulos M, Als D, Gaffey M F, Kamali M, *et al*. Delivering infectious disease interventions to women and children in conflict settings: a systematic review. *BMJ Global Health* 2020; 5:e001967. https://doi:10.1136/bmjgh-2019-001967
39. Lakwo T, Oguttu D, Ukety T, Post R, Bakajika D. Onchocerciasis Elimination: Progress and Challenges. *Res Rep Trop Med.* 2020;11:81-95 http://doi.org/10.2147/RRTM.S224364
40. News. We've paid some Fulani to stop killings in Southern Kaduna - El-Rufai. Vanguard Newspaper 2016 December 3 [Cited 2023 September 10]. Available

fromhttps://www.google.com/url?sa=t&source=web&rct=j&opi=89978449&url=https://www.vanguardngr.com/2016/12/weve-paid-fulani-stop-killings-southern-kaduna-el-rufai/&ved=2ahUKEwjFuLv1s5OIAxVyQkEAHVPaCngQFnoECB0QAQ&usg=AOvVaw2jsptX99yvtN0xdxpwb3MT

41. Traore MO, Sarr MD, Badji A, Bissan Y, Diawara L, Doumbia K, et al. Proof-of-Principle of Onchocerciasis Elimination with Ivermectin Treatment in Endemic Foci in Africa: Final Results of a Study in Mali and Senegal. PLoS Negl Trop Dis 2012; 6(9): e1825. https://doi:10.1371/journal.pntd.0001825
42. Nicholls RS, Duque S, Olaya LA, Lopez MC, Sanchez SB, Morales AL, et al. Elimination of onchocerciasis from Colombia: first proof of concept of river blindness elimination in the world. *Parasit. Vectors* 2018 April 11; 11:237 https://doi.org/10.1186/s13071-018-2821-9
43. Guevara A, Lovato R, Proaño R, Rodriguez-Perez MA, Unnasch T, Cooper PJ, et al. Elimination of onchocerciasis in Ecuador: findings of post-treatment surveillance. *Parasit Vector*2018 April 24e; 11:265 https://doi.org/10.1186/s13071-018-2851-3
44. Richard F, Rizzo N, Espinoza CED, Monroy ZM, Valdez CGC, de Cabrera RM, et al. One hundred years after its discovery in Guatemala by Rodolfo Robles, *Onchocerca volvulus* transmission has been eliminated from the Central Endemic Zone. Am. J. Trop. Med. Hyg. 2015 October 26, 93(6):1295–1304. https://doi:10.4269/ajtmh.15-0364
45. Rodriguez-Perez MA, Fernandez-Santos NA, Orozco-Algarra ME, Rodríguez-Atanacio JA, Domínguez-Vázquez A, Rodríguez-Morales KB, et al. Elimination of onchocerciasis from Mexico. *PLoS Negl Trop Dis*. 2015; 9(7):e0003922. https://doi:10.1371/journal.pntd.0003922

Latest Manuscript Click here to access/download;Manuscript;Latest Manuscript.docx

Recrudescence of transmission of onchocerciasis in some endemic communities in Kaduna State, Nigeria: What is the way forward?

Timothy O. Olanrewaju[1*], Felicia N. C. Enwezor[1], , Luret A. Lar[2], Michael A. Igbe[3], Ramatu A. Abdullahi[1],Monsuru A. Adeleke[4], Oluwatosin B. Adekeye[5], Elizabeth O. Elhassan[6]

[1]Nigerian Institute for Trypanosomiasis Research (*and Onchocerciasis*) Research, Kaduna, State, Nigeria

[2]Department of Community Medicine, Faculty of Clinical Sciences, College of Health Sciences, University of Jos, Plateau State, Nigeria

[3]Federal Ministry of Health, Federal Capital Territory, Abuja, Nigeria

[4]Department of Zoology, Faculty of Basic and Applied Sciences, Osun State University, Osogbo, Osun State, Nigeria

[5]Department of Psychiatry, Ahmadu Bello University Teaching Hospital Shika, Zaria, Kaduna State, Nigeria

[6]1A Mohammed Yusuf Lere Drive, Off Tafawa Balewa Way, Ungwan Rimi, Kaduna State, Nigeria

Corresponding author: timothyseye@gmail.com (TOO)

Abstract

Background

Onchocerciasis caused by the filarial parasite *Onchocerca volvulus* and transmitted by *Simulium damnosum* s.l. remains a public health concern in Nigeria. Infestation of *S. damnosum s.l.* along rivers Gurara and Kaduna; and heavy intensity of *O. volvulus* infection in Kaduna were documented in 1956. Control of onchocerciasis in Kaduna started 1954 with larviciding using dichlorodiphenyltrichloroethane which reduced *S. damnosum s.l.* population by 94% in 1966. Diethylcarbamazine was also used for human treatment but discontinued. Kaduna State Ministry of Health and its partners built on this achievement; used annual mass administration of ivermectin consistently between 1989 and 2017 which led to interruption of onchocerciasis transmission in 2018. This study investigated possibility of recrudescence of onchocerciasis with the hypothesis that insecurity-induced migration could cause recrudescence of onchocerciasis.

Methodology/Principal Findings

Six out of the 23 Local Government Areas (LGAs) in Kaduna State were selected for evaluation. Adult *S. damnosum* s.l. were captured across seven breeding sites using human landing collectors from July to October 2023. Pooled screen assays of 72 pools heads of black flies were conducted using quantitative polymerase chain reaction (qPCR) for *O. volvulus* detection. Dried blood samples from 3107 children aged 5–9 years were collected; with 1502 samples analysed using alkaline phosphatase enzyme-linked immunosorbent assay due to financial constraints. Twenty pools of the 72 pools of heads (27.8%) of *S. damnosum s.l.* analysed were positive for *O.*

volvulus in Kagarko and Kachia LGAs at 95% UCL 0.49 infectivity (> 1/2000 infective flies) with overall prevalence of 0.32. Two children from security compromised communities tested seropositive (prevalence 0.31%; 95% UCL 0.317).

Conclusion/Significance

The findings demonstrated ongoing onchocerciasis transmission in Kaduna State despite the interruption in 2018. This calls for programmatic evaluation of the extent of recrudescence and identification of key drivers such as human migration, fly movement and insecurity.

Keywords: Onchocerciasis, Kaduna State, recrudescence, *Simulium damnosum s.l.*, ivermectin, *Onchocerca volvulus*

Author summary

Onchocerciasis is a disease that affects humans and is transmitted by bites of *Onchocerca volvulus* infected black flies. Nigeria accounts for the highest onchocercal blindness in sub-Saharan Africa. Collaborative effort by various stakeholders in Kaduna State, Nigeria yielded remarkable achievement of successful interruption of onchocerciasis transmission in year 2018 through annual mass administration of medicine (MAM). However, our study established the ongoing transmission of onchocerciasis in some areas of Kaduna State after four years stop MAM decision. Kaduna State Government, partners and stakeholders should urgently take actions to mitigate the ongoing transmission of onchocerciasis in the State to safeguard the achievements attained after years of MAM. We recommend the following; assessment of entire Kaduna State to determine the extent of the spread of the recrudescence; in-depth analysis of

annual therapeutic coverage rates; community-driven slash and clear approach for vector control; review of the monitoring and evaluation system across all levels; using Esperanza Window Trap at black flies breeding sites to complement the slash and clear approach; modelling the approaches to determine the most appropriate frequency of treatment with ivermectin.

Introduction

Human infection with the filarial parasite *Onchocerca volvulus* causes onchocerciasis (also known as river blindness). *Onchocerca volvulus* is transmitted to humans by bites of *Simulium damnosum s.l.* (black flies) in Nigeria. There are nine sibling species within the *S. damnosum* complex that can transmit onchocerciasis [1, 2]. Onchocerciasis is one of the Neglected Tropical Diseases (NTDs) with high public health impact in Africa. Over 99% of all cases of onchocerciasis and onchocercal-related blindness are found in Africa; with Nigeria having about 40% of the global population at risk [3]. Onchocercal blindness ranked second leading cause of preventable blindness in the world after trachoma [4]. The disease is peculiar to the poor with economic implications resulting in abandonment of rich fertile farmlands which resulted in food insecurity and poor school attendance, sometimes forcing children to drop out of school to assist blind parents/guardians. Other manifestations include; skin disfiguration and stigmatization. These impact on the wellbeing of individuals with the disease become a major challenge to the achievement of Sustainable Development Goals (SDGs); “End hunger, achieve food security and improved nutrition and promote sustainable agriculture”(SDG 2) and “Ensure healthy lives and promote well-being for all at all ages (SDG3) as documented by Engels [5].

Crosskey [6] documented that adult *S. damnosum s.l.* dispersal ability is estimated to be 234,000 Km^2 (90,000 square miles) in Northern Nigeria. Nearly half of the areas infested by *S. damnosum s.l.* in Northern Nigeria lies within region of rivers Gurara and Kaduna which is about 37,400 Km^2 (14,400 square miles). The control of onchocerciasis in Kaduna State started in 1954 with larviciding using dichlorodiphenyltrichloroethane (DDT) along rivers Gurara and Kaduna [7, 8]. By 1966, this control effort had achieved 94% reduction in *S. damnosum s.l.* population density. Diethylcarbamazine was also used for human treatment but discontinued due to high toxicity. The State started annual MAM of ivermectin in 1989. Tekle et al. [9] reported that 17years of consistent annual administration of ivermectin could potentially interrupt transmission of onchocerciasis in two foci (Birnin Gwari and Kauru/Lere) within Kaduna State. By 2018, interruption of transmission of the disease was attained and MAM implementation was stopped [10].

However, the current security challenges which revolve around ethno-religious and farmer-herder conflicts over land access heighten violence in Kaduna State [11]; migration to the State from the border States like Niger, Kano and Federal Capital Tertiary (FCT) Abuja where transmission of onchocerciasis is still ongoing [12] and vector migration due to climate change could cause recrudescence of onchocerciasis in Kaduna State. According to the International Organization for Migration report [13], Kaduna State has a minimum of 117,880 internally displaced persons (IDPs) as a result of banditry and kidnapping (45%), communal clashes (29%), natural disaster (13%), farmers-herders clashes (12%), and insurgency (2%).Our interaction with the State Emergency Management Agency (SEMA) revealed that the only State government managed IDP camp is located at Giwa Local Government Area (LGA). However, we identified a

faith-based IDP camp at Agwan Zawu, Gonin-Gora, Chikun LGA and another at Evangelical Church Winning All (ECWA), Ungwan Musa, Zonkwa, Zangon-Kataf LGA. Interruption of transmission of onchocerciasis in Kaduna State might be difficult to sustain because of high number of IDPs within the State without any organized or governmental camp. Also, increase in inter-state migration within northwest region of Nigeria and climate change could affect transmission of onchocerciasis. These challenges could jeopardise efforts towards 2030 target of elimination of onchocerciasis, other targets in the NTD Roadmap 2021-2023 and SDG 2 and 3.

We investigated possible recrudescence of onchocerciasis transmission in six LGAs in Kaduna State in the face of insecurity and human migration four years after interruption of transmission.

Methods

Study area

Kaduna State lies between 10°36’33” N and 7°25’46” E and shares boundaries with Kano and Katsina States in the north; Plateau State to the east; Nasarawa State to the south; Niger State in the west and the FCT Abuja to the southwest. Some of the major rivers in the State include river Gurara and river Kaduna which serve as good breeding sites for black flies [14]. All of the States that share boundaries with Kaduna are currently treating with ivermectin except Plateau and Nasarawa States, which have interrupted transmission, and Katsina State which was hypo endemic for onchocerciasis and was ineligible for treatment requires onchocerciasis elimination mapping (OEM). Kaduna State consists of 23 Local Government Areas (LGAs) with a projected population of more than six million people and estimated land area of about 46,063 Km^{2}. The

demographic vegetation of the State is mainly savannah grassland with few areas of forest. This study was carried out in six LGAs of Kaduna State, namely Chikun, Kachia, Kaduna North, Kaduna South, Kagarko and Zangon-Kataf (Fig. 1). Kaduna North and Kaduna South LGAs were selected due to the presence of black fly breeding sites at river Kaduna that runs through the two LGAs and influx of migrants from Chikun LGA. Chikun, Kachia and Kagarko LGAs were included because of the presence of black flies breeding sites along rivers Gurara and Kaduna; insecurity and presence of migrants. Zangon-Kataf LGA was chosen based on high level of insecurity and proximity to breeding site in Kachia. Seven breeding sites across five LGAs serve as catching points of black flies namely Kabala Doki (Kaduna North LGA), Kwata (Kaduna South LGA), Tsallake (Chikun LGA), Atara and Dogon-Daji (Kagarko LGA), Amuse and Gurara (Kachia LGA) as shown in Table 1.

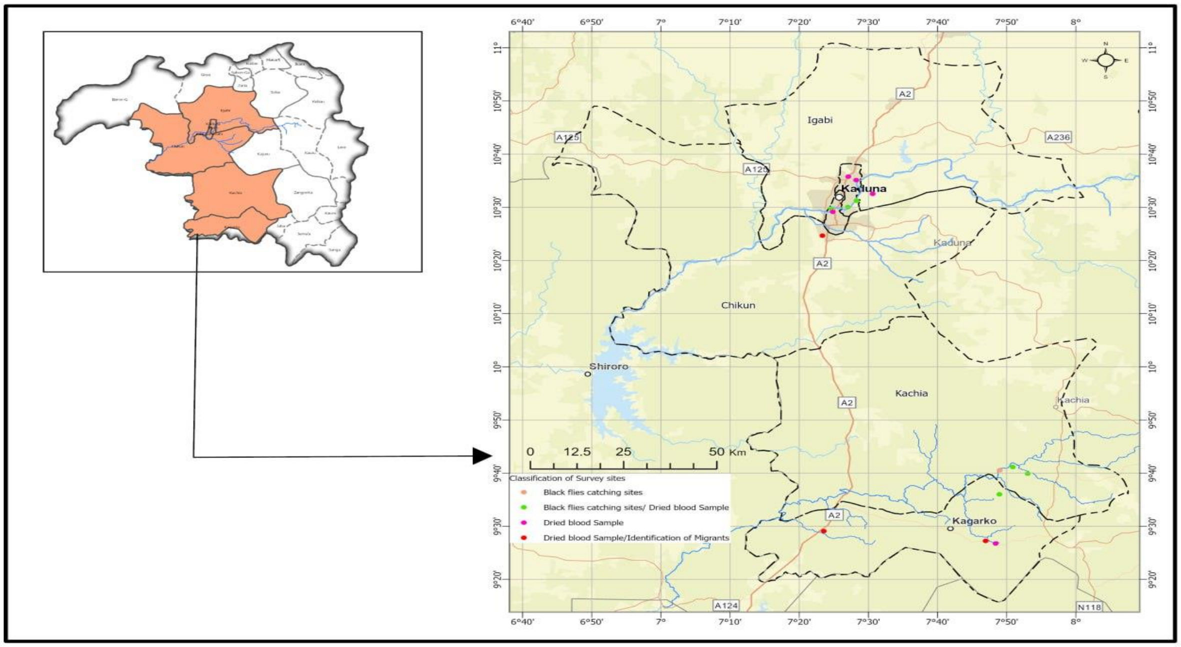

Fig 1: Map of Kaduna State showing the study locations (ArcGIS Pro Software version 3.2.0; https://www.arcgis.com/home/item.html?id=0387d2dd20204e51b382f871aff0fb9d)

Epidemiological evaluation

The epidemiological study was carried out in all the selected LGAs. Sample collection was done between July and October, 2023.

Study population

Farming is the major occupation of citizens at Chikun, Kagarko, Kachia and Zangon-Kataf, but for Kaduna South and Kaduna North LGAs majority are civil servants and business men/women. Dried blood samples were collected from children (male and female) between 5 and 9 years old across the study areas.

Sample size and sample collection

Dried blood samples (DBS) were collected from 3107 children aged 5-9 years from various communities and primary schools across the six LGAs using World Health Organization (WHO) guidelines for epidemiological assessment of onchocerciasis transmission in children [15, 16]. The sample from each community was estimated proportionally to available children within the required age group. Blood samples were collected from all assented children aged 5-9 years through finger-prick using sterile lancet after swabbing the finger with methylated spirit; a lancet to each child. About 100 µL of blood from each child was spotted on Whatman number 1 filter paper (Whatman International Ltd Maldstone, England); using one filter paper for a child. Each of the filter papers was fixed to a Styrofoam using pencil for air-drying. For each community, each child's sample was stored in a small ziplock bag and then put into a larger ziplock bag containing silica gel (Desicare, Reno, NV, USA), labelled appropriately and store at 25°C.

Sample analysis

The samples were analysed within maximum of six months of sample collection at Osun State University Multi-Disciplinary Research Laboratory, Osogbo, Nigeria using AP-ELISA technique as detailed in S1 Methods

Ethical approval

The study protocol was approved by the Health Research Ethics Committee (HREC) of Kaduna State Ministry of Health (Ref No. NHREC/17/03/2018). Verbal assent was obtained from each child with verbal consent of either parent or guardian of the child before blood sample collection. Volunteers that acted as human attractants for black fly catching were told about the personal risks and community benefits of participation and given the option to opt out of participation at any time without any repercussions after signing the participant consent form. They were offered ivermectin at the end of the study according to specifications.

Entomological evaluation

Seven known breeding sites of *S. damnosum s.l.* in five LGAs – Chikun, Kachia, Kaduna North, Kaduna South and Kagarko were assessed to ascertain if they were currently breeding the vector. The coordinates of the breeding sites were taken using Garmin geographical positioning information system (GPIS) machine. Based on the outcome of the assessments, community leaders of first-line communities (communities within 20km to *S. damnosum s.l.* breeding site) and second-line communities (communities within 20km – 40km away *S. damnosum s.l.* breeding site) were sensitized on the importance of preventing blindness due to onchocerciasis. The leaders identified two persons from their communities that serve as human landing

collectors (HLCs) for *S. damnosum s.l.* The HLCs were clustered into two groups for a two days theory and practical training on capturing of black flies and recording as described [17, 18]. Black fly capturing activities were conducted between July and October 2023 being the peak of black fly breeding season. Two HLCs worked in an alternate arrangement hourly for two days per week between 07:00 h and 18:00 h. The flies collected were stored in preservative bottles containing isopropyl alcohol. The bottles were labelled accordingly for each catching site. At the end of capturing of the flies, the study entomologist sorted all the flies and confirms the flies that were *S. damnosum s.l.* using morphological characteristics such as conspicuous compound eyes, short horn-like antennae, hump-backed at midsection of the thorax and broad, colourless and transparent wings. The black flies were stored in isopropyl alcohol and hand carried to the Multi-disciplinary Research Laboratory, Osogbo, Nigeria to determine the *O. volvulus* infectivity rate in black flies using real-time qPCR technique assay with a mitochondrial DNA target as detailed in S2 Methods. The separation of the heads from the bodies, extraction and qPCR were carried out as described [19].

Statistical Analysis

The number of black fly collected in each location was normalized and then subjected to One - way Analysis of Variance using SPSS version 23.0 to determine the significant difference in fly variation across catching sites and also the months of collection as detailed in S3 Methods. The Ov16 antibody prevalence in children was determined using 95% confidence intervals of > 0.1% upper confidence limit (UCL) as described [20] using the formula:

95% Confidence interval = proportion positive (p) ± $1.96(\frac{\sqrt{P(1-\mathrm{P})}}{\mathrm{n}})$. The poolscreen software 2.1 was used to determine *O. volvulus* infectivity rate in pools of black flies at 95% confidence interval.

Results

Out of the 1,502 DBS of children aged 5 – 9 years; two children from Kagarko LGA were Ov16 sero-positive with prevalence of 0.13% (95% UCL 0.317) as shown in Tables 1. These children were from first-line and second-line communities, black fly breeding sites; and communities with IDPs and migrants. The two Ov16 sero-positive samples were from Atara community (first-line community) and Kubacha community (second-line community) within Kagarko LGA. Six thousand eight hundred and ninety-seven (6,897) flies out of 7,782 reported flies from the field were identified as *S. damnosum s.l.* (Table 2).The highest number 2,858 (71.48%) of black flies captured was from Kuratam community followed by 2,072 (30.04%) in Atara within Kagarko LGA. Gurara and Amuse communities had 1,181 (17.12%) and 782 (11.33%) respectively in Kachia LGA. Only four black flies were captured along river Kaduna; two at Kabala and two at Kinkinau. We observed a significant variation in the number of flies captured at various catching sites during the wet season as the number of black fly collected at Kuratam, Atara, Gurara and Amuse were significantly higher (*p>0.05*) than those collected in Kabala, Kinkinau and Babban Saura (Table 2). There was significant variation (*p>0.05*) in the number of flies collected during the four months catching period (Table 3).

Twenty pools of the 72 pools of heads (27.8%) of *S. damnosum s.l.* analysed were positive for *O. volvulus* at 95% UCL 0.49 of black flies infectivity (> 1/2000 infective flies) with overall prevalence of 0.32 (Table 4). Four out of the seven black flies catching sites (57.14%) tested

positive with both sites in Kagarko and Kachia LGAs testing positive. We observed the highest infectivity rate (33%) in black flies captured at Amuse catching site in Kachia LGA.

Table 1: Dried blood sample Ov16 antibody/antigen test using AP-enzyme linked immunosorbent assay

Local government area	Community	Proximity to black fly breeding site	Number of children sampled	No. of DBS analysed	No. of positive DBS
**Kaduna North**	Kabala	First line	304	86	0
	Angwan-Gwari	Second line	136	136	0
	Rafin-Guza	Second line	160	100	0
	Kawo	Second line	106	0	0
**Kaduna South**	Kinkunu	First line	176	100	0
	Sheilk Gumi Primary School	Second line	233	0	0
	Down Quarters	Second line	136	135	0
	Television	Second line	134	0	0
	Aliyu Makarma Primary School	Second line	276	0	0
**Chikun**	Angwan Zawu IDP camp	Second line	23	23	0
	Doka Mai Jama'	First line	137	137	0
	UBE Bagado Primary School	Second line	122	0	0
	Babban Saura	First line	237	120	0
**Kagarko**	Kuratam	First line	231	231	0
	Atara	First line	76	76	1
	Kubacha	Second line	100	100	1
	Dogo Kurumi	Second line	71	71	0
**Kachia**	Gurara	First line	49	49	0
	Zonkwa Primary School	Second line	262	0	0
**ZangonKataf**	Samaru Kataf	Second line	138	138	0
**TOTAL**			**3107**	**1,502**	**2**
	**Lower and Upper Limit CI (95%)**				**-0.051 - 0.368**

The 95% UCL of OV-16 positivity > 0.1% indicates ongoing transmission of Onchocerciasis in Kaduna State

Table 2: Human landing capturing of flies at various breeding sites in the study areas

Local Government Area	Community	Catching Site	Number of fly catchers	Number of flies captured in the field	Number of confirmed black flies in the laboratory	Number of pool	Black fly population density in wet season (%)	Mean + SD ($p < 0.05$)
**Kaduna North**	Kabala	Kabala	2	148	2	1	1.4	37.00+8.25^{a}
**Kaduna South**	Kinkinau	Kwata	2	27	2	1	7.4	6.75+3.86^{a}
**Chikun**	Babban Saura	Tsallake	2	11	0	0	0	2.75+1.89^{a}
	Atara	Atara	2	2233	2072	21	92.8	558.25+148.30^{b}
**Kagarko**	Kuratam	Dogon Daji	2	2924	2858	29	97.7	731.00+70.77^{c}
	Amuse	Gurara	2	1175	782	8	66.6	293.75+113.72^{d}
**Kachia**	Gurara	Gurara	2	1264	1181	12	93.4	316.00+8.36^{d}
**TOTAL**		**7**	**14**	**7,782**	**6,897**	**72**		

The mean ± SD (standard deviation) with different superscript along the same column indicates statistically significant difference ($p < 0.05$).

Table 3: Variation in fly population density during wet season

Local Government Area	Community	July	August	September	October
**Kaduna North**	Kabala Doki	30	42	46	30
**Kaduna South**	Kinkinau	12	7	5	3
**Chikun**	Babban Saura	0	4	3	4
**Kagarko**	Atara	587	619	683	344
	Kuratam	650	760	813	701
**Kachia**	Amuse	387	349	309	130
	Gurara	318	333	293	320
**Total**		**1984 (25.5%)**	**2114 (27.2%)**	**2152 (27.7%)**	**1532 (19.70%)**

Table 4: Detection of *Onchocerca volvulus* DNA signal in *S. damnosum s.l.* pools using qPCR

Local government area	Community	No. of black flies analysed	No. of pools of 100	No. of pools < 100	No. of positive pools	Prevalence by Ov ND5 qPCR (95% confidence interval)
**Kaduna North**	Kabala Doki	2	0	1(2)	0	0.00 (0.00)
**Kaduna South**	Kinkinau	2	0	1(2)	0	0.00 (0.00)
**Chikun**	Tsalake	0	0	0	0	0.00 (0.00)
**Kagarko**	Kuratam	2,858	28	1(58)	7(24%)	0.0275 (0.13-0.545)
	Atara	2,072	20	1(72)	7(33%)	0.044 (0.19 – 0.79)
**Kachia**	Gurara	1,181	11	1(81)	3(25%)	0.0287 (0.09-0.76)
	Amuse	782	7	1(82)	3(38%)	0.047 (0.15-1.18)
**TOTAL**		**6,897**	**66**	**6**	**20**	**0.32 (0.21-0.49)**

The 95% UCL of black flies infectivity of >1/2,000 infective flies/2,000 indicates ongoing transmission. CT-values for pools of black flies are detailed in S1 Results

Discussion

Black flies prefer breeding in conducive river systems where the physico-chemical parameters of the rivers support their establishment [7]. The results of the present study showed that the river system in Kaduna indeed support the breeding of the black flies, though with variation as evident in the current study. Historical data showed that Davies et al. [7] carried out larviciding using DDT along river Gurara from Suleja, Abuja to Tafa due to the high biting density of black fly along the river. This resulted to 94% reduction in the population density of *S. damnosum* in the area. On the one hand, the high number of flies collected along river Gurara still suggests that breeding of black fly continues along the river. On the other hand, the high water level in river Kaduna resulted in low density of black fly in Chikun, Kaduna North and Kaduna South LGAs despite a four months attempt to capture black flies. The low density of black fly could be attributed to flooding, inadequate rocky basements and low velocity of water. According to Opara et al [21] and Eyo et al [22] heavy rainfall and stormy weather play major role in washing away most black flies breeding sites during the rainy season. Inadequate rapids and rocky

basements which form favourable breeding sites for black flies [23] might also affect the population density. Furthermore, high water level during heavy rain can slow down the velocity of water which reduces the favourable conditions for breeding of *Simulium* species [24]. Though the breeding sites along river Gurara were probably not visited during cytotaxonomic identification of members of *S. damnosum* complex [25], breeding of black fly and transmission of onchocerciasis along the river at Gantang in Kagarko LGA has been documented [14]. The high population density of black fly recorded in Amuse, Gurara, Atara and Kuratam sites could be as a result of vegetation, rocks, speed of the water and other favourable physico-chemical parameters of the river Gurara [26, 27]. The population density along river Gurara corroborates the earlier report [14]. The high infectivity rate in black flies we observed in Amuse, Atara, Gurara and Kuratam suggests that transmission cycle of onchocerciasis has been sustained for awhile in Kachia and Kagarko LGAs. This contradicts the previous documentation [10] that no transmission of *O. volvulus* in Kaduna State; thus recrudescence of onchocerciasis established in the State. There is possibility of imported infection from the neighbouring Niger State and FCT Abuja to Kaduna State. According to Crosskey [6], rivers Gurara, Kaduna, Sarkin Pawa (in Niger State) are always infested with black fly with rivers Gurara and Kaduna forming major fly area of approximately 140 miles (225 km). The ability of *S. damnosum* complex to migrate >400 km away in large numbers [1, 28, 29] due to seasonal variation can threaten the interruption of transmission of onchocerciasis and elimination of the disease in Kaduna State.

To curb the black fly population density, slash and clear approach in Kaduna and the neighbouring endemic areas can be adopted. This approach requires removal of vegetation from breeding sites (fast flowing well oxygenated sediment free water) along rivers to disrupt *S.*

damnosum breeding activities. Smith et al. [30] recommended slash and clear using community-driven implementation strategy at least once a year for accelerating achievement of onchocerciasis elimination. Slash and clear vector control using community-driven implementation strategy at Maridi dam spillway in South Sudan reduced *S. damnosum* by >90% six months post intervention and <50% twelve months post intervention [31]. The approach has also been used in Uganda with 97% reduction in biting rate after single intervention during early wet season [32] and Cameroon with 32.9% reduction in black fly population density [33]. Combination of community directed treatment with ivermectin (CDTi) with slash and clear vector control will accelerate achievement of onchocerciasis in Africa. The use of Esperanza Window Trap can also be deployed along black flies breeding sites to complement the slash and clear approach as described [34].

Despite the on-going treatment with ivemectin for Lymphatic filariasis in Kachia and Kagarko LGAs after stopping treatment for onchocerciasis in 2018, the 0.13% Ov16 serological test result in children aged 5-9 years coupled with overall infectivity rate of 0.32 (C.I. 0.21-0.49) which is above the WHO threshold of <1/2000 infective rate in black flies) indicates ongoing transmission of onchocerciasis in the study area. This implies that there is recrudescence of onchocerciasis in Kaduna State despite the interruption of transmission of onchocerciasis recorded in year 2018. The reasons for these observations may be attributed to insecurity, human migration and diagnostic technique used. The WHO onchocerciasis technical advisory subgroup in its sixth meeting [35] observed that real time qPCR assay used in this study has a higher sensitive than O-150 PCR ELISA and could detect residual transmission in fly pools negative for O-150 PCR ELISA. Furthermore, in a recent publication by Adeleke et al [34] on a

comparative study of OvND5 qPCR and O-150 PCR ELISA in three ecological zones in Nigeria, they reported that OvND5 qPCR is more sensitive. More black fly pools were positive for *O. volvulus* by OvND5 qPCR compared with O-150 PCR in derived savannah (31.15 vs. 15.57%), montane forest (11.54 vs. 0%) and rainforest (23.08 vs. 2.56%).

Searching beyond the diagnostic technique used, the climate change with great impact on black flies' movement and human migration (intra and interstate) as a result of communal conflicts, political crisis, kidnapping and insurgency are also potential threats to elimination of onchocerciasis [37, 38, 39]. The earlier therapeutic coverage of 65% with ivermectin attained in Kaduna State before stopping MAM [10] might be insufficient to break the transmission cycle considering the high infectivity rate of black flies in some part of the State.

Kagarko, Kachia and other LGAs in southern part of Kaduna State have had several episodes of insecurity since 2011 [40]. During the period of insecurity, the population in the affected communities moved out to other LGAs within the State and Niger State then returned when the situation is calm. The migration of people from affected communities poses a threat to elimination of onchocerciasis in other LGAs that share boundaries with the LGAs in Kaduna; Tafa and Suleja in Niger States and Bwari in FCT, Abuja. This calls for a review of geographic and therapeutic coverage rates, implementation of community self monitoring and intensify monitoring and evaluation of treatment. There is need for development of model that will determine appropriate frequency of treatment for Kaduna State. Adoption of biannual treatment in River Gambia, Senegal (April or May and October or November) for more than 10 years has been successfully implemented with desired outcomes [41]. Also, the biannual

community directed treatment with ivermectin resulted to elimination of onchocerciasis in Colombia in 2013 [42]; Ecuador in 2014 [43] and Guatemala in 2016 [44]. To achieve elimination of onchocerciasis, Mexico changed frequency of treatment with ivermectin to quarterly and achieved elimination in year 2015 [45].

Limitations of the study

The limitations of the study include inability to analyse the 3107 dried blood samples (DBS) collected from children aged 5-7years because of limited resources, devaluation of the naira, inflation and high cost of reagents during this study. Other limitations were failure to collect DBS from children in Amuse; a first-line community with the highest infectivity rate in black flies due to insecurity during the period of the study and inability to collect black flies in the dry season as a result of the short duration of the study.

Conclusion

In conclusion, the high *O. volvulus* infectivity rate (above WHO threshold) in black flies and positive samples of DBS in children in some of the communities sampled showed that there is an ongoing transmission in the study area. This, by implication established the recrudescence of the disease after interruption of the disease was achieved in Kaduna in 2018. This calls for urgent programmatic evaluation of the extent of recrudescence (since our study only covered the southern Kaduna) and the key drivers of the recrudescence. Does the recrudescence being driven by socio-cultural, human migration, fly movement or large scale of insecurity are issues awaiting elucidation through further studies.

Supporting information

S1 Methods: Detailed protocol for Ov16 ELISA

S2 Methods: Detailed protocol for DNA isolation from black fly heads and qPCR

S3 Methods: Detailed statistical variation in fly population density at various sites and across various months

S1 Results: Detailed CT-values for pools of black flies examined

Author contributions

Conceptualization: Felicia N. C. Enwezor, Timothy O. Olanrewaju, Elizabeth O. Elhassan

Data Curation: Timothy O. Olanrewaju, Felicia N. C. Enwezor, Oluwatosin B. Adekeye

Methodology: Timothy O. Olanrewaju, Felicia N. C. Enwezor, Michael Igbe

Project Administration: Timothy O. Olanrewaju, Felicia N. C. Enwezor

Funding Acquisition: Timothy O. Olanrewaju, Felicia N. C. Enwezor, Elizabeth O. Elhassan, Luret A. Lar, Oluwatosin B. Adekeye

Investigation: Timothy O. Olanrewaju, Felicia N. C. Enwezor, Michael Igbe, Ramatu A. Abdullahi

Formal Analysis: Monsuru A. Adeleke, Michael Igbe

Supervision: Felicia N. C. Enwezor, Elizabeth O. Elhassan, Luret A. Lar

Validation: Monsuru A. Adeleke, Luret A. Lar, Felicia N. C. Enwezor, Oluwatosin B. Adekeye

Writing-Original Draft: Timothy O. Olanrewaju, Michael Igbe, Luret A. Lar

Writing-Review and Editing: Timothy O. Olanrewaju, Felicia N. C. Enwezor, Luret A. Lar, Ramatu A. Abdullahi, Micheal Igbe, Monsuru A. Adeleke, Oluwatosin B. Adekeye, Elizabeth O. Elhassan.

Funding

This work received financial support from the Coalition for Operation Research on Neglected Tropical Diseases (COR-NTD), which is funded at The Task Force for Global Health primarily by the Bill and Melinda Gates Foundation and the United States Agency for International Development through its Neglected Tropical Diseases Program. The grant was administered by the African Research Network for Neglected Tropical Diseases under Small Grant Program-VI (SGP159/050.2023) to TOO. The funders had no role in study design, data collection and analysis, decision to publish, or preparation of the manuscript.

Acknowledgment

We acknowledge Sightsavers, Kaduna Office and Kaduna State Ministry of Health for availing us essential information during the design of the study protocols and sample collection. Also, Nigerian Institute for Trypanosomiasis Research, Kaduna for the provision of the vehicle used during field activities. Osun State University Multi-disciplinary Research Laboratory, Osogbo, Nigeria provided additional reagents for Ov16 serological test and qPCR analysis of *S. damnosum s.l.*

Conflict of Interest

The authors declare no conflict of interest during this study.

References

1. Post KJ, Onyenwe E, Somiari SA, Mafuyai HB, Crainey JL, Ubachakwu PO. A guide to the *Simulium damnosum* complex (Diptera: Simuliidae) in Nigeria, with a cyto taxonomic key for the identification of the sibling species. *Ann. Trop. Med. Parasitol*. 2011 Jun 1; 105(4):277-297. https://doi.org/10.1179/136485911x12987676649700PMID: 21871165
2. Chikekie FM, Opara KN, Ubulom PME, Yaro CA, Al-Akeel RK, Osei-Atweneboana MY et al. Onchocerciasis transmission status in some endemic communities of mass drug administration with ivermectin. *Sci Rep*. 2023; 13:5413 doi: 10.1038/s41598-023-31446-6PMID: 37012274
3. World Health Organization. Ending the neglect to attain the Sustainable Development Goals: A roadmap for neglected tropical diseases 2021–2030. Available: https://apps.who.int/iris/rest/bitstreams/1326801/retrieve [Accessed June 15, 2023].
4. World Health Organization. Onchocerciasis: Fact sheet 2022. https://www.who.int/news-room/fact [Accessed September 12, 2023]
5. Engels D. Neglected tropical diseases in the Sustainable Development Goals. *The Lancet* 2016 January 16; 387(10015), 223-224. https://doi:1016/s104-6736(16)00043-x
6. Crosskey RW. The distribution of *Simulium damnosum* Theobald in northern Nigeria. *Trans. Royal Society Trop. Med. Hyg*. 1956; 50: 379–392
7. Davies JB. The *Simulium* control scheme at Abuja, Northern Nigeria and its effect on the prevalence of onchocerciasis in the area. *Bull. World Health Org*. 1968; 39:187-207
8. Bump JB. The Crosskey-Davies Experiment and Onchocerciasis Control in West Africa. *PLoS Negl Trop Dis* 2014 October 9; 8(10): e3223. https://doi:10.1371/journal.pntd.0003223
9. Tekle AH, Elhassan E, Isiyaku S, Amazigo UV, Bush S, et al. Impact of long-term treatment of onchocerciasis with ivermectin in Kaduna State, Nigeria: first evidence of the potential for elimination in the operational area of the African Programme for Onchocerciasis Control. *Parasit Vectors* 2012 Feb 7; 5:28. https://doi:10.1186/1756-3305-5-28
10. Isiyaku S, Igbe M, Madaki S, Hamill LC, Ndongmo P, Adamani W, et al. The interruption of transmission of onchocerciasis in Kaduna, Kebbi and Zamfara State, Nigeria: another milestone achievement. *Intern. Health* 2022 May 12; 14(2):ii43-ii54. https://doi.org/10.1093/inthealth/ihac036
11. Olajumoke A. (2021). Confronting Nigeria's Kaduna crisis https://africacenter.org/spotlight/confronting-nigerias-kaduna-crisis [Accessed February 20, 2022].
12. Yahaya A, Kela SL, Suleiman MM, Sama'ila AB. Prevalence studies on Onchocerciaisis (River Blindness) in some villages from Three Senatorial Districts, Kano State, Nigeria. *South Asian Journal Parasitol.* 2021*;* 5(4):141-152; Article no. SAJP 80294
13. Displacement Tracking Matrix (DTM) of the International Organization forMigration (IOM). IOM Nigeria-North-central and North-west zones displacement report. 2023; Round 12 needs monitoring. Available: https://dtm.iom.int/Nigeria [Accessed May 8, 2024]

14. Isiyaku S, Adamani W, Louise H, Ndams IS, Ezekiel K, Kal, A, et al. Entomological assessment of the Impact of long-term mass administration of ivermectin in onchocerciasis-endemic communities in Kaduna State, Nigeria. [Preprint]. 2021 [cited 2022 August 21]. Available from: https://doi.org/10.21203/rs.3.rs-840823/v1
15. World Health Organization. Guidelines for Stopping Mass Drug Administration and Verifying Elimination of Human Onchocerciasis: Criteria and Procedures. *Geneva, Switzerland*: WHO 2016.[Google Scholar]
16. Nigerian Federal Ministry of Health. Nigeria Onchocerciasis elimination plan. *Abuja: Federal Ministry of Health*; 2017.
17. Service MW. Methods for sampling adult *Simuliidae,* with special reference to the *Simulium damnosum* complex*. Tropical Pest Bulletin* 1977 (5). Centre for Overseas Pest Research, London, UK; ISBN 0-85135-087-9 http://gala.gre.ac.uk/12219
18. Adeleke MA, Mafiana CF, Sam-Wobo SO, Olatunde GO, Ekpo UF, Akinwale OP et al. Biting behaviour of *Simulium damnosum* complex and *Onchocerca volvulus* infection along Osun River, Southwest Nigeria. *Parasit Vector 2010 October 7;* 3(93): 1-5. doi:10.1186/1756-3305-3-93 http://www.parasitesandvectors.com/content/3/1/93
19. Hendy A, Kruger A, Pfarr K, De Witte J, Kibweja A, Mwingira U, et al. The black fly vectors and transmission of *Onchocerca volvulus* in Mahenge, south eastern Tanzania. *Acta Trop.* 2018 February 2; 181:50–9. https://doi.org/10.1016/j.actatropica.2018.01.009
20. Richard FO, Katabarwa M, Bekele F, Tadesse Z, Mohammed A, Sauerbrey M, et al. Operational performance of the *Onchocerca volvulus* "OEPA" OV16 ELISA serological assay in mapping, stop mass drug administration and post treatment surveillance survey. *Am J Trop Med Hyg.*2018;99(3):747–752 doi: 10.4269/ajtmh.18-0341
21. Opara, K.N., Fagbemi, O.B., Ekwe, A., and Okenu, D.M (2005). Status of forest Onchocerciasis In the lower cross river basin Nigeria. Entomologic profile after five years of Ivermectin Intervention. *Am. J. Trop. Med. Hyg*73(2) pp 371-376.
22. Eyo, E.J., Ikechukwu, E.O., Ubachukwu, P.O., Ivoke, N., Ekeh, F.N (2014). Effects of climatic conditions on the biting density and relative abundance of *Simulium damnosum* complex in a rural Nigerian farm settlement. *Annals of Agricutural and Environmental Medicine vol 21 no 4 697-700*.
23. Nanjul GO, Chinedu OU, Job N, Meshach JS, Apollos WP, Adams C, et al. Oviposition and Cues in black flies: Possible clues for boom of adult population. *Inter. J. Mosquito Res.* 2017 February 17; 4(2):111-124. ISSN: 2348-5906
24. Ikpeama CA, Nwoke BEB, Anosike, JC, Orji, NM and Onyirioha CN. Studies on the biting behaviours female *Simulium damnosum* complex in some endemic areas of Imo State, Nigeria. *Inter. Wld. J. Sci. Tech.* 2007; 3:1-5
25. Mafuyai HB, Post RJ, Vajime CG, Molyneux DH. Cytotaxonomic identifications of the *Simulium damnosum* complex (Diptera: Simuliidae) from Nigeria. *Trop. Med Intern. Health* 1996; 1(6):779-785

26. Ibeh OO, Nwoke BEB, Adegoke JA. Distribution and ecology of breeding sites of *Simulium damnosum s.l*. in south–eastern primary health zone of Nigeria. Nigerian Journal of Parasitology 2007; 28:32–38.
27. Oforka LC, Adeleke MA, Anikwe JC, Makanjuola WA. Population fluctuations and effect of climatic factors on the relative abundance of *Simulium damnosum* complex (Diptera: Simuliidae). Environ. Entomol. 2019; 48: 284–290.
28. Walsh JF, Davies JB, Garms R. Further studies on the reinvasion of the onchocerciasis control programme by *Simulium damnosum s.l.*: the effects of an extension of control activities into southern Ivory Coast during 1979. Tropenmedizin und Parasitologie 1981; 32, 269–273.
29. Baker RHA, Guillet P, Se´ke´te´li A, Poudiougo P, Boakye D et al. Progress in controlling the reinvasion of windborne vectors into the western area of the Onchocerciasis Control Programme in West Africa. Philosophical Transactions of the Royal Society of London. 1990; Series B, 328, 731–750.
30. Smith ME, Bilal S, Lakwo TL, Habomugisha P, Tukahebwa E, ByamukamaE, et al. Accelerating river blindness elimination by supplementing MDA with a vegetation "slash and clear" vector control strategy: a data-driven modeling analysis. *Sci Rep*. 2019; 9:15274. https://doi:10.1038/s41598-019-51835-0
31. Raimon S, Lakwo TL, Sebit WJ, Siewe Fodjo JN, Alinda P, et al. "Slash and Clear", a Community- Based Vector Control Method to Reduce Onchocerciasis Transmission by *Simulium sirbanum* in Maridi, South Sudan: A Prospective Study. Pathogens 2021 October 15; 10, 1329. https://doi.org/10.3390/pathogens10101329
32. Jacob B, Loum D, Munu D, Lakwo T, Byamukama, E, Habomugisha P, et al. Optimization of Slash and Clear Community-Directed Control of *Simulium damnosum Sensu Stricto* in Northern Uganda. Am. J. Trop. Med. Hyg. 2021, 104, 1394–1403. https://doi:10.4269/ajtmh.20-1104
33. Domche A, Djeunga HCN, Nwane PB, Njitchouang GR, Fesuh BN, Njiokou F, et al. Significant reduction of black fly densities in persistent onchocerciasis area following pilot implementation of an environment friendly approach (slash and clear). *Sci. Reports* 2024; 14:408 https://doi.org/10.1038/s41598-023-50747-4
34. Adeleke MA, Opara KN, Mafuyai HB, Nwoke BEB, Surakat OA, Akinde SB, et al. Improving onchocerciasis elimination surveillance: trials of odour baited Esperanza Window Traps to collect black fly vectors and real-time qPCR detection of Onchocerca volvulus in black fly pools. *Parasit Vector* 2024 November 18, 17:471 https://doi.org/10.1186/s13071-024-06554-5
35. WHO Report of the Sixth meeting of the WHO onchocerciasis technical advisory subgroup: virtual meeting, 19 – 21 December 2022. Geneva: WHO; 2023
36. Doherty M, Grant JR, Pilotte N, Bennuru S, Fischer K, Fischer PU, et al. Optimized strategy for real-time qPCR detection of *Onchocerca volvulus* DNA in pooled *Simulium sp*. Black fly vectors. *PLoS Negl Trop Dis* 2023 17(12):e0011815. https://doi.org/10.1371/journal. pntd.00118 15
37. Coninx R. Tuberculosis in complex emergencies. *Bull World Health Organ.* 2007; 85:637–40.

38. Meteke S, Stefopulos M, Als D, Gaffey M F, Kamali M, *et al*. Delivering infectious disease interventions to women and children in conflict settings: a systematic review. *BMJ Global Health* 2020; 5:e001967. https://doi:10.1136/bmjgh-2019-001967
39. Lakwo T, Oguttu D, Ukety T, Post R, Bakajika D. Onchocerciasis Elimination: Progress and Challenges. *Res Rep Trop Med.* 2020;11:81-95 http://doi.org/10.2147/RRTM.S224364
40. News. We've paid some Fulani to stop killings in Southern Kaduna - El-Rufai. Vanguard Newspaper 2016 December 3 [Cited 2023 September 10]. Available fromhttps://www.google.com/url?sa=t&source=web&rct=j&opi=89978449&url=https://www.vanguardngr.com/2016/12/weve-paid-fulani-stop-killings-southern-kaduna-el-rufai/&ved=2ahUKEwjFuLv1s5OIAxVyQkEAHVPaCngQFnoECB0QAQ&usg=AOvVaw2jsptX99yvtN0xdxpwb3MT
41. Traore MO, Sarr MD, Badji A, Bissan Y, Diawara L, Doumbia K, et al. Proof-of-Principle of Onchocerciasis Elimination with Ivermectin Treatment in Endemic Foci in Africa: Final Results of a Study in Mali and Senegal. PLoS Negl Trop Dis 2012; 6(9): e1825. https://doi:10.1371/journal.pntd.0001825
42. Nicholls RS, Duque S, Olaya LA, Lopez MC, Sanchez SB, Morales AL, et al. Elimination of onchocerciasis from Colombia: first proof of concept of river blindness elimination in the world. *Parasit. Vectors* 2018 April 11; 11:237 https://doi.org/10.1186/s13071-018-2821-9
43. Guevara A, Lovato R, Proaño R, Rodriguez-Perez MA, Unnasch T, Cooper PJ, et al. Elimination of onchocerciasis in Ecuador: findings of post-treatment surveillance. *Parasit Vector*2018 April 24e; 11:265 https://doi.org/10.1186/s13071-018-2851-3
44. Richard F, Rizzo N, Espinoza CED, Monroy ZM, Valdez CGC, de Cabrera RM, et al. One hundred years after its discovery in Guatemala by Rodolfo Robles, *Onchocerca volvulus* transmission has been eliminated from the Central Endemic Zone. Am. J. Trop. Med. Hyg. 2015 October 26, 93(6):1295–1304. https://doi:10.4269/ajtmh.15-0364
45. Rodriguez-Perez MA, Fernandez-Santos NA, Orozco-Algarra ME, Rodríguez-Atanacio JA, Domínguez-Vázquez A, Rodríguez-Morales KB, et al. Elimination of onchocerciasis from Mexico. *PLoS Negl Trop Dis*. 2015; 9(7):e0003922. https://doi:10.1371/journal.pntd.0003922

Figure

Click here to access/download;Figure;Map of Kaduna State showing sampling points.tif

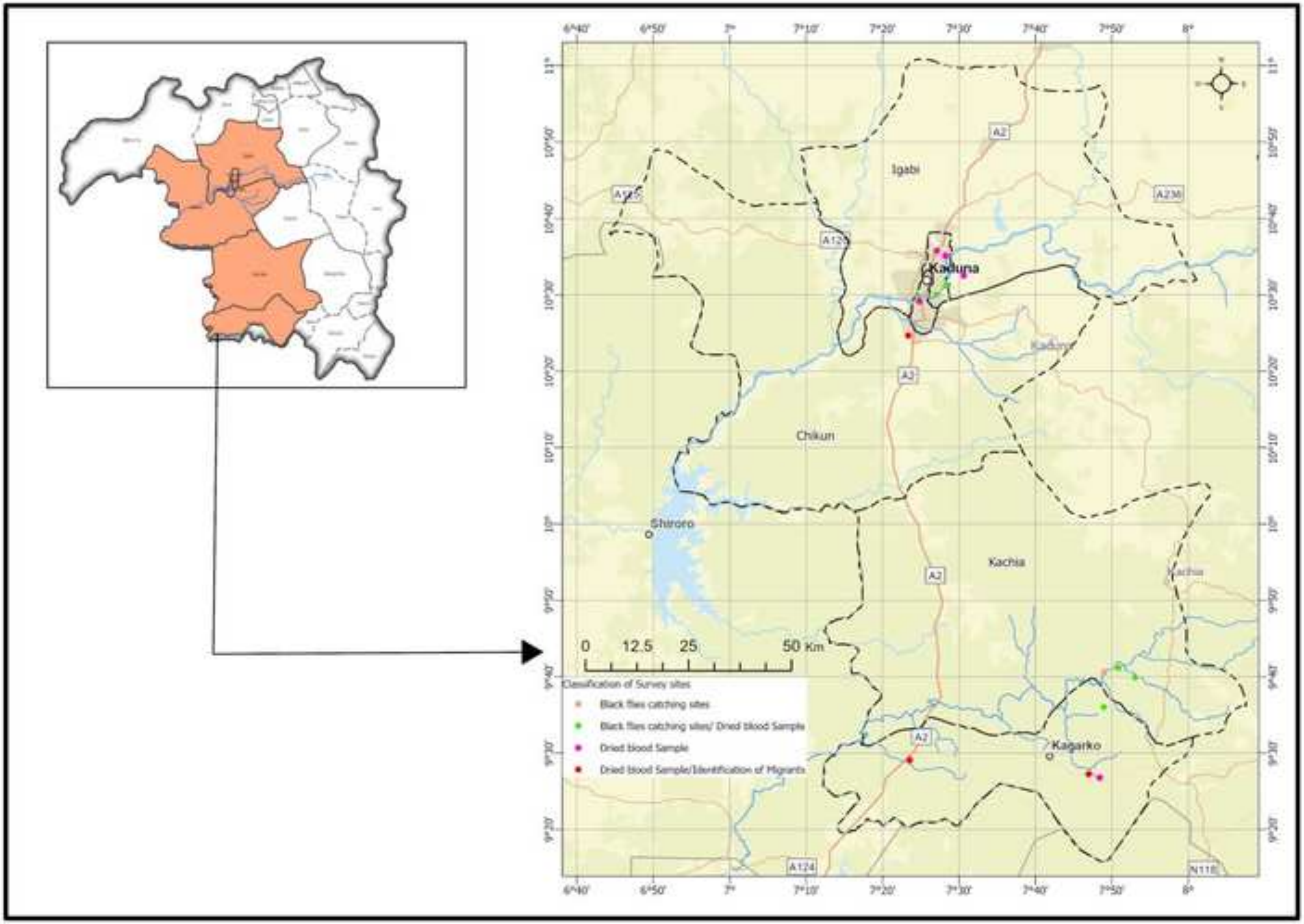

 Click here to access/download;Table;Table 1.docx

Table 1: Dried blood sample Ov16 antibody/antigen test using AP-enzyme linked immunosorbent assay

Local government area	Community	Proximity to black fly breeding site	Number of children sampled	No. of DBS analysed	No. of positive DBS
Kaduna North	Kabala	First line	304	86	0
	Angwan-Gwari	Second line	136	136	0
	Rafin-Guza	Second line	160	100	0
	Kawo	Second line	106	0	0
Kaduna South	Kinkunu	First line	176	100	0
	Sheilk Gumi Primary School	Second line	233	0	0
	Down Quarters	Second line	136	135	0
	Television	Second line	134	0	0
	Aliyu Makarma Primary School	Second line	276	0	0
Chikun	Angwan Zawu IDP camp	Second line	23	23	0
	Doka Mai Jama'	First line	137	137	0
	UBE Bagado Primary School	Second line	122	0	0
	Babban Saura	First line	237	120	0
Kagarko	Kuratam	First line	231	231	0
	Atara	First line	76	76	1
	Kubacha	Second line	100	100	1
	Dogo Kurumi	Second line	71	71	0
Kachia	Gurara	First line	49	49	0
	Zonkwa Primary School	Second line	262	0	0
ZangonKataf	Samaru Kataf	Second line	138	138	0
**TOTAL**			**3107**	**1,502**	**2**
	**Lower and Upper Limit CI (95%)**				**-0.051 - 0.368**

The 95% UCL of OV-16 positivity > 0.1% indicates ongoing transmission of Onchocerciasis in Kaduna State

Table 2: Human landing capturing of flies at various breeding sites in the study areas

Local Government Area	Community	Catching Site	Number of fly catchers	Number of flies captured in the field	Number of confirmed black flies in the laboratory	Number of pool	Black fly population density in wet season (%)	Mean + SD ($p < 0.05$)
**Kaduna North**	Kabala	Kabala	2	148	2	1	1.4	37.00+8.25[a]
**Kaduna South**	Kinkinau	Kwata	2	27	2	1	7.4	6.75+3.86[a]
**Chikun**	Babban Saura	Tsallake	2	11	0	0	0	2.75+1.89[a]
	Atara	Atara	2	2233	2072	21	92.8	558.25+148.30[b]
**Kagarko**	Kuratam	Dogon Daji	2	2924	2858	29	97.7	731.00+70.77[c]
	Amuse	Gurara	2	1175	782	8	66.6	293.75+113.72[d]
**Kachia**	Gurara	Gurara	2	1264	1181	12	93.4	316.00+8.36[d]
	**TOTAL**	**7**	**14**	**7,782**	**6,897**	**72**		

The mean ± SD (standard deviation) with different superscript along the same column indicates statistically significant difference ($p < 0.05$).

 Click here to access/download;Table;Table 3.docx

Table 3: Variation in fly population density during wet season

Local Government Area	Community	July	August	September	October
**Kaduna North**	Kabala Doki	30	42	46	30
**Kaduna South**	Kinkinau	12	7	5	3
**Chikun**	Babban Saura	0	4	3	4
**Kagarko**	Atara	587	619	683	344
	Kuratam	650	760	813	701
**Kachia**	Amuse	387	349	309	130
	Gurara	318	333	293	320
**Total**		**1984 (25.5%)**	**2114 (27.2%)**	**2152 (27.7%)**	**1532 (19.70%)**

Table Click here to access/download;Table;Table 4.docx

Table 4: Detection of *Onchocerca volvulus* DNA signal in *S. damnosum s.l.* pools using qPCR

Local government area	Community	No. of black flies analysed	No. of pools of 100	No. of pools < 100	No. of positive pools	Prevalence by Ov ND5 qPCR (95% confidence interval)
**Kaduna North**	Kabala Doki	2	0	1(2)	0	0.00 (0.00)
**Kaduna South**	Kinkinau	2	0	1(2)	0	0.00 (0.00)
**Chikun**	Tsalake	0	0	0	0	0.00 (0.00)
**Kagarko**	Kuratam	2,858	28	1(58)	7(24%)	0.0275 (0.13-0.545)
	Atara	2,072	20	1(72)	7(33%)	0.044 (0.19 – 0.79)
**Kachia**	Gurara	1,181	11	1(81)	3(25%)	0.0287 (0.09-0.76)
	Amuse	782	7	1(82)	3(38%)	0.047 (0.15-1.18)
**TOTAL**		**6,897**	**66**	**6**	**20**	**0.32 (0.21-0.49)**

The 95% UCL of black flies infectivity of >1/2,000 infective flies/2,000 indicates ongoing transmission. CT-values for pools of black flies are detailed in S1 Results

Click here to access/download
Supporting Information
Supporting information S1 _Methods_Ov16 ELISA_edited.docx

Click here to access/download
Supporting Information
Suppoting information S2_Methods_qPCR_edited.docx

Click here to access/download
Supporting Information
Suppoting information R1_Results_qPCR_PNTD-D-edited.xlsx

Click here to access/download
Supporting Information
Supporting information S3_Methods_Statistic_PNTD-D-24-01243.docx

NIGERIAN INSTITUTE FOR TRYPANOSOMIASIS (*AND ONCHOCERCIASIS*) RESEARCH

No. 1 Surame Road, UngwanRimi GRA, Kaduna, Kaduna State, Nigeria

10th January, 2025

Dear Editor,
PLoS NTD

Responses to Reviewers' Comments: PNTD-D-24-01243

Reference to the reviewers' comments on our manuscript with reference PNTD-D-24-01243, the authors responses are as follow:

Manuscript Number: PNTD-D-24-01243

Reviewers' Comment	Authors' responses
Suggested Title: **Recrudescence of transmission of onchocerciasis in some endemic communities in Kaduna State, Nigeria: What is the way forward**	Accepted
The background of the abstract on the cover page is not well written and should be revised.	The Abstract has been revised accordingly
**Introduction**<br><br>**Lines 89-91:** Note that Tekle et al., 2012 did not claim the foci to have attained elimination based on skin snip microscopy. The survey did not follow the prevailing WHO Certification guidelines for onchocerciasis elimination. However, it is safe to mention that annual treatment with ivermectin could potentially interrupt transmission and eliminate onchocerciasis.<br><br>**Methods:**<br>(1) DBS samples were not collected from some communities, especially, Amuse community in the Kachia LGA. It is interesting to note that Amuse recorded the highest black fly infectivity rate in this	This has been revised accordingly (Lines 89 – 91)<br><br>**Limitation of the study**:<br>Samples were not collected from children in Amuse; first-line community with the highest infectivity rate in black flies due to high level

study but had no data on DBS. Can the authors explain why no DBS samples were collected from this community?	of insecurity around the community during sample collection
(2) The Ov16 AP-ELISA methods was adequately described, however, the protocol requires some revision due to some omissions in its description, particularly when compared with the Golden et al. (2016) protocol. For example, certain wash steps are missing, and point 9 is unclear, it states that it was only "after incubation, that the plate was washed four times with PBST using a wash bottle. Also, the rationale for using zip-lock bags during incubation should be clarified, whether this is due to the absence of plate sealers or if there is a specific purpose for their use. The manuscript does not specify the wavelength at which the plates were read, which is crucial for reproducibility. Therefore, the authors should clarify whether the protocol is adapted from an existing method or they are presenting a novel approach for Ov16 AP-ELISA.	The detail protocol has been attached as supporting document.
**Results**:	
(1) The worth of data obtained from this study does not commensurate the results generated in this manuscript. A detailed statistical analysis of the data would have presented some more interesting results. Therefore, data analysis by the authors are not adequate for the kind of study carried out, unless the authors have already published some of the data elsewhere or have plans of publishing some of the data elsewhere.	Results obtained from detail statistical analysis has been included in the manuscript.
(2) The authors should provide some images on the qPCR results indicating the Ct – values.	Real time qPCR Ct-values for pools of black flies at various catching sites has been attached as supporting document for results

(3) The map of the study area is unclear and should be replaced.	A clearer map of the study area has been included in the manuscript.
(4) A comparison between AP-ELISA results (the OV16 sero-prevalence from DBS) and qPCR results (from pooled black fly heads) varied significantly. For instance, sero-prevalence of 0.31% compared to black flies infectivity rates of 27.78%. Can the authors provide some explanation for these results?	The statistical analysis of black flies included indicates overall prevalence of 0.32 with infectivity rate of 27.78%. Usually infectivity rate will be higher in the vector compared to human because more than twenty flies can feed on one positive human.
**Discussion:**	
(1) Lines 235-246: The authors attributed the low fly density in Chikun, Kaduna North and Kaduna South LGAs during the four months black fly sampling period to the high-water level in river Kaduna, and further indicated that these were as a result of the effect of urbanization, climate change and pollution. However, the authors did not consider the possible effect of seasonal changes on black fly densities, especially low water level during dry seasons. It has been observed that at high-water level, some rivers produce few fly numbers, but at moderate to low river levels, some rapids and breeding sites are created leading to production of more black fly numbers.The author should therefore check previous data on fly density during dry seasons in Chikun, Kaduna North and Kaduna South LGAs, when water level in river Kaduna were moderate to low.	It has been revised accordingly (Lines 249 – 257)
(2) Line 278-283: The claim by authors that O-150 PCR-ELISA is not sensitive to establish interruption of onchocerciasis transmission is incorrect, and cannot be assigned as a reason for ongoing transmission in the Kaduna State. It must be noted that the same test	This has been revised accordingly with references including WHO report on OvND5 qPCR assays (288- 302)

had been used to confirm interruption of transmission in many countries. Moreover, recent and currently studies using O-150 and OvND5qPCR assays collaborate very well. There is a standard recommendation by the WHO for onchocerciasis elimination. Is OvND5 PCR that standard? Has it been approved for elimination decision making? There are indications that this particular target does not separate *O. volvulus* from the animal *Onchocerca ochengi* as it pools for both species<br><br>**Conclusion**:<br>Some of the conclusions are not well supported by the data presented. The authors did not effectively link some of their findings to the evidence they provided, this should be done.<br><br>Authors should clearly state the limitations of the study.	The conclusion has been revised accordingly and limitations of the study clearly stated.

Yours sincerely,

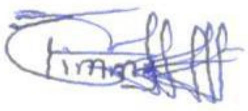

Timothy O. OLANREWAJU
Corresponding Author
timothyseye@gmail.com

Click here to access/download
Revised Article with Changes Highlighted
Revised Manuscript with Track Changes.docx

Click here to access/download
Revised Article with Changes Highlighted
Latest Revised Manuscript with Track Changes.docx
