# Supplementary material for: Recrudescence of transmission of onchocerciasis in some endemic communities in Kaduna State, Nigeria: What is the way forward?
Source: PLoS Negl Trop Dis. 2025 Aug 7;19(8):e0012495. doi: 10.1371/journal.pntd.0012495 (PMC12331076; doi:10.1371/journal.pntd.0012495)
Supplement: Attachment — Submitted filename: Review - attachment.docx [file pntd.0012495.s005.docx]

**Recrudescence of transmission of onchocerciasis in Kaduna State, Nigeria: What is the way forward?**

**Introduction**

**Lines 89-91:** Note that Tekle et al., 2012 did not claim the foci to have attained elimination based on skin snip microscopy. The survey did not follow the prevailing WHO Certification guidelines for onchocerciasis elimination. However, it is safe to mention that annual treatment with ivermectin could potentially interrupt transmission and eliminate onchocerciasis.

**Line 217:**

**Discussion**

**Lines 242-244:** The high-water level and low fly density in Chukun could imply flooding of breeding sites, and it takes some time for them to recover. Is Chukun an urban area? If so, then urbanization and pollution can also be a problem for Simulium breeding. I think making these statements clear will make the paragraph easy to understand.

**Lines 259-264:** It was unclear whether the two foci mentioned above had been properly delineated and well-isolated from other transmission zones. If it is true that they were isolated, could it be human or fly population migration from areas with ongoing transmission? It seems this area requires further investigation in the future if you are to get to the bottom of the problem. I think the authors have hinted at insecurity that could be responsible for the human population movements, but what conditions are there to influence fly migration? It seems that the success of ivermectin treatment complemented by EWTs and Slash and Clear depends on applying the same intervention approaches in Kaduna and the neighboring endemic areas with ongoing transmission.

Models sound like a good idea, but they require adequate data, which takes a long time to generate. You may be better off to recommend delineating your study areas and the neighboring endemic areas likely responsible for the recrudescence. Then, you recommend deploying appropriate and well-coordinated interventions to achieve transmission interruption across all affected areas in the same but large transmission zone.
