# Supplementary material for: Recrudescence of transmission of onchocerciasis in some endemic communities in Kaduna State, Nigeria: What is the way forward?
Source: PLoS Negl Trop Dis. 2025 Aug 7;19(8):e0012495. doi: 10.1371/journal.pntd.0012495 (PMC12331076; doi:10.1371/journal.pntd.0012495)
Supplement: Attachment — Submitted filename: Reviewers Comments_ Osei-Atweneboana_(1).docx [file pntd.0012495.s007.docx]

**Reviewer’s name : Professor Mike Yaw Osei-Atweneboana**

Manuscript Number: PNTD-D-24-01243

**Manuscript title: Recrudescence of transmission of onchocerciasis in Kaduna State,**

**Nigeria: What is the way forward**

**General Comments**

This is a comprehensive study, with the goal of determining recrudescence of onchocerciasis transmission in Kaduna State of Nigeria. The study employed entomological, serological and molecular biology techniques to determine blackfly infectivity in pooled blackfly heads using qPCR and collection of dried blood spot from human population to carry out OV16 AP-ELISA to assess seroprevalence in children aged 5-9 years in six of 23 local government areas (LGAs) in Kaduna State.

This is an important study because it has demonstrated ongoing transmission in Kaduna State, where interruption of onchocerciasis transmission had been previously established in 2018. This posses a big threat to the onchocerciasis elimination targeted in 2030. This has been attributed to recent insecurity and the presence of migrants from other States where there could be ongoing transmission.

Nonetheless, the manuscript has a lot of technical, statistical and other challenges, and **requires major revisions before it can be accepted for publication.** In general, the manuscript is well written but have some grammatical mistakes and eros, these have been indicated in my review.

**Title of the manuscript: Recrudescence of transmission of onchocerciasis in Kaduna State, Nigeria: What is the way forward**

The title is appropriate but must be revised, Kaduna State has 23 LGAs, of which the authors investigated six. The results showed that only four communities located in two of the six LGAs investigated were positive for seroprevalence and/or blackfly infectivity. Considering that only four communities were positive, that title of the manuscript should be revised to read: **Recrudescence of transmission of onchocerciasis in some endemic communities in Kaduna State, Nigeria: What is the way forward**

**Abstract on the cover page**: The background of the abstract on the cover page is not well written and should be revised

**Objective of the study**: The objective of the study is clearly defined, articulated and appropriate for the study, however, no clear testable hypothesis was stated.

**Methods**:

The study area and population were well described, the study design is appropriate to address the stated objectives. The sample size was sufficient to ensure adequate statistical power to address the objectives, however, due to financial challenges encountered by the authors, not all samples collected were analyzed.

**Sampling of dried blood spot (DBS):** DBS samples were not collected from some communities, especially, Amuse community in the Kachia LGA. It is interesting to note that Amuse recorded the highest blackfly infectivity rate in this study but had no data on DBS. Can the authors explain why no DBS samples were collected from this community.

The Ov16 AP-ELISA methods was adequately described, however, the protocol requires some revision due to some omissions in its description, particularly when compared with the Golden et al. (2016) protocol. For example, certain wash steps are missing, and point 9 is unclear, it states that it was only "after incubation, that the plate was washed four times with PBST using a wash bottle.

Also, the rationale for using zip-lock bags during incubation should be clarified, whether this is due to the absence of plate sealers or if there is a specific purpose for their use.

The manuscript does not specify the wavelength at which the plates were read, which is crucial for reproducibility.

Therefore, the authors should clarify whether the protocol is adapted from an existing method or they are presenting a novel approach for OV 16 AP-ELISA.

Ethical approval was obtained for the study.

**Results**:

The worth of data obtained from this study does not commensurate the results generated in this manuscript. A detailed statistical analysis of the data would have presented some more interesting results. Therefore, data analysis by the authors are not adequate for the kind of study carried out, unless the authors have already published some of the data elsewhere or have plans of publishing some of the data elsewhere.

The map of the study area is unclear and should be replaced. Additionally, Figure 1 and Table 4 were not mentioned in the document.

Table 3: Human Landing Capture of Flies at Various Breeding Sites in the Study Areas:

The sub-headings in columns 6; “Number of unsorted flies captured” should be revised to read “Number of flies captured” while in columns 6; “Number of sorted black flies” should read “Number of blackflies captured”

Table 4: Real-Time Quantitative Polymerase Chain Reaction Assay of *S. damnosum s.l.:*

The title of the table should be revised to appropriately reflect the qPCR analysis. For example, Detection of *Onchocerca volvulus* DNA signal in *S. damnosum s.l.* pools using qPCR.

A comparison between AP-ELISA results (the OV16 sero-prevalence from DBS) and qPCR results (from pooled blackfly heads) varied significantly. for instance, sero-prevalence of 0.31% compared to blackflies infectivity rates of 27.78%. Can the authors provide some explanation for these results

Also, the authors should provide some images on the qPCR results indicating the Ct – values.

**Discussion:**

Lines 235-246: The authors attributed the low fly density in Chikun, Kaduna North and Kaduna South LGAs during the four months blackfly sampling period to the high-water level in river Kaduna, and further indicated that these were as a result of the effect of urbanization, climate change and pollution. However, the authors did not consider the possible effect of seasonal changes on blackfly densities, especially low water level during dry seasons. It has been observed that at high-water level, some rivers produce few fly numbers, but at moderate to low river levels, some rapids and breeding sites are created leading to production of more blackfly numbers.

The author should therefore check previous data on fly density during dry seasons in Chikun, Kaduna North and Kaduna South LGAs, when water level in river Kaduna were moderate to low.

Line 278-283: The claim by authors that O-150 PCR-ELISA is not sensitive to establish interruption of onchocerciasis transmission is incorrect, and cannot be assigned as a reason for ongoing transmission in the Kaduna State. It must be noted that the same test had been used to confirm interruption of transmission in many countries. Moreover, recent and currently studies using O-150 and OvND5 qPCR assays collaborate very well.

**Conclusion**:

Some of the conclusions are not well supported by the data presented. The authors did not effectively link some of their findings to the evidence they provided, this should be done.

Authors should clearly state the limitations of the study.

**Some corrections to be made by the authors.**

Line 76 – Check for corrections, "s" after "farmlands" should be deleted.

Lines 80-82 : Some spelling mistakes in SDGs; "End hunger, achieved food security should read "End hunger, achieve food security”

Ensure health lives should read Ensure healthy lives."

Line 102 – Is "LGA" an acronym? If so, please write in full.

Line 109 – What does "DG 2 and 3" mean? Does this refer to "SDG 2 and 3"?

Line 117 – Is "FC) Abuja" the same as "FCT Abuja." Please make the necessary correction.

Lines 131-132 – Connect the breeding sites to the LGAs and cite the appropriate table.

Line 135 – Provide a clearer, well-labelled version of the map.

Line 143 – Does "LGs" mean "LGAs"? otherwise define or ensure consistency.

Line 161 – What does "DBS" mean? Please write in full

Lines 171-173 – Were the vector collectors signed consent forms ? If yes, please state it clearly.

Line 182 – Write "HLCs" in full

Lines 187-190 – Elaborate on how the morphological assessment was conducted. Were the samples (*S. damnosum*) stored in isopropyl alcohol after assessment? Clarify whether ethanol (as stated in the supplementary qPCR) or isopropyl alcohol (as stated in the manuscript method) was used.

Line 194 – Which software version was used: 2.1 (as stated in the manuscript) or 2.0 (as stated in the supplementary for qPCR)?

Line 232 – Table 4 is not cited in the results. Please cite it appropriately.

Line 241: The phrase “the large number of fly” should read “the large number of flies.”

Lines 246-248: Please check the font size of the sentences; “Changes in the geographical distributions of different cytospecies of the *Simulium damnosum*” used a larger font size and should be adjusted to match the rest of the text.

Line 274 – Please write "CDTi." In full

The authors should list all primer sequences in the methods section or in the supplementary materials.

They should also specify the CT values obtained for the positive controls in the supplementary materials and explain why any CT value below 40 was considered positive with reference to the positive controls.
