# Supplementary material for: Recrudescence of transmission of onchocerciasis in some endemic communities in Kaduna State, Nigeria: What is the way forward?
Source: PLoS Negl Trop Dis. 2025 Aug 7;19(8):e0012495. doi: 10.1371/journal.pntd.0012495 (PMC12331076; doi:10.1371/journal.pntd.0012495)
Supplement: Attachment — Submitted filename: Response to Reviewers.docx [file pntd.0012495.s012.docx]

**NIGERIAN INSTITUTE FOR TRYPANOSOMIASIS (*AND ONCHOCERCIASIS*) RESEARCH**

No. 1 Surame Road, UngwanRimi GRA, Kaduna, Kaduna State, Nigeria

10^th^ January, 2025

Dear Editor,

PLoS NTD

**Responses to Reviewers’ Comments: PNTD-D-24-01243**

Reference to the reviewers’ comments on our manuscript with reference PNTD-D-24-01243, the authors responses are as follow:

**Manuscript Number: PNTD-D-24-01243**

| **Reviewers’ Comment** | **Authors’ responses** |
| --- | --- |
| Suggested Title: **Recrudescence of transmission of onchocerciasis in some endemic communities in Kaduna State, Nigeria: What is the way forward** | Accepted |
| The background of the abstract on the cover page is not well written and should be revised. | The Abstract has been revised accordingly |
| **Introduction**  **Lines 89-91:** Note that Tekle et al., 2012 did not claim the foci to have attained elimination based on skin snip microscopy. The survey did not follow the prevailing WHO Certification guidelines for onchocerciasis elimination. However, it is safe to mention that annual treatment with ivermectin could potentially interrupt transmission and eliminate onchocerciasis.  **Methods:**   (1) DBS samples were not collected from some communities, especially, Amuse community in the Kachia LGA. It is interesting to note that Amuse recorded the highest black fly infectivity rate in this study but had no data on DBS. Can the authors explain why no DBS samples were collected from this community? (2) The Ov16 AP-ELISA methods was adequately described, however, the protocol requires some revision due to some omissions in its description, particularly when compared with the Golden et al. (2016) protocol. For example, certain wash steps are missing, and point 9 is unclear, it states that it was only "after incubation, that the plate was washed four times with PBST using a wash bottle. Also, the rationale for using zip-lock bags during incubation should be clarified, whether this is due to the absence of plate sealers or if there is a specific purpose for their use. The manuscript does not specify the wavelength at which the plates were read, which is crucial for reproducibility. Therefore, the authors should clarify whether the protocol is adapted from an existing method or they are presenting a novel approach for Ov16 AP-ELISA.   **Results**:   (1) The worth of data obtained from this study does not commensurate the results generated in this manuscript. A detailed statistical analysis of the data would have presented some more interesting results. Therefore, data analysis by the authors are not adequate for the kind of study carried out, unless the authors have already published some of the data elsewhere or have plans of publishing some of the data elsewhere. (2) The authors should provide some images on the qPCR results indicating the Ct – values. (3) The map of the study area is unclear and should be replaced. (4) A comparison between AP-ELISA results (the OV16 sero-prevalence from DBS) and qPCR results (from pooled black fly heads) varied significantly. For instance, sero-prevalence of 0.31% compared to black flies infectivity rates of 27.78%. Can the authors provide some explanation for these results?   **Discussion:**   (1) Lines 235-246: The authors attributed the low fly density in Chikun, Kaduna North and Kaduna South LGAs during the four months black fly sampling period to the high-water level in river Kaduna, and further indicated that these were as a result of the effect of urbanization, climate change and pollution. However, the authors did not consider the possible effect of seasonal changes on black fly densities, especially low water level during dry seasons. It has been observed that at high-water level, some rivers produce few fly numbers, but at moderate to low river levels, some rapids and breeding sites are created leading to production of more black fly numbers.The author should therefore check previous data on fly density during dry seasons in Chikun, Kaduna North and Kaduna South LGAs, when water level in river Kaduna were moderate to low. (2) Line 278-283: The claim by authors that O-150 PCR-ELISA is not sensitive to establish interruption of onchocerciasis transmission is incorrect, and cannot be assigned as a reason for ongoing transmission in the Kaduna State. It must be noted that the same test had been used to confirm interruption of transmission in many countries. Moreover, recent and currently studies using O-150 and OvND5qPCR assays collaborate very well. There is a standard recommendation by the WHO for onchocerciasis elimination. Is OvND5 PCR that standard? Has it been approved for elimination decision making? There are indications that this particular target does not separate *O. volvulus* from the animal *Onchocerca ochengi* as it pools for both species   **Conclusion**:  Some of the conclusions are not well supported by the data presented. The authors did not effectively link some of their findings to the evidence they provided, this should be done.  Authors should clearly state the limitations of the study. | This has been revised accordingly (Lines 89 – 91)  **Limitation of the study**:  Samples were not collected from children in Amuse; first-line community with the highest infectivity rate in black flies due to high level of insecurity around the community during sample collection  The detail protocol has been attached as supporting document.  Results obtained from detail statistical analysis has been included in the manuscript.  Real time qPCR Ct-values for pools of black flies at various catching sites has been attached as supporting document for results  A clearer map of the study area has been included in the manuscript.  The statistical analysis of black flies included indicates overall prevalence of 0.32 with infectivity rate of 27.78%. Usually infectivity rate will be higher in the vector compared to human because more than twenty flies can feed on one positive human.  It has been revised accordingly (Lines 249 – 257)  This has been revised accordingly with references including WHO report on OvND5 qPCR assays (288- 302)  The conclusion has been revised accordingly and limitations of the study clearly stated. |

Yours sincerely,

**Timothy O. OLANREWAJU**

Corresponding Author

[timothyseye@gmail.com](mailto:timothyseye@gmail.com)
