# Supplementary material for: Recrudescence of transmission of onchocerciasis in some endemic communities in Kaduna State, Nigeria: What is the way forward?
Source: PLoS Negl Trop Dis. 2025 Aug 7;19(8):e0012495. doi: 10.1371/journal.pntd.0012495 (PMC12331076; doi:10.1371/journal.pntd.0012495)
Supplement: Attachment — Submitted filename: PNTD-D-24-01234- MK Mrach 1- 2025.docx [file pntd.0012495.s010.docx]

PLOS Neglected Tropical Diseases

**Manuscript Number: PNTD-D-24-01243R1**

**Title: Recrudescence of transmission of onchocerciasis in some endemic communities in Kaduna State, Nigeria: What is the way forward?**

1) **Recommendation term (Major Revision)**

**Abstract:**

**Methodology/Principal Findings**

**34**	It is not clear why six out of 23 LGAs in Kaduna State were selected for evaluation. Do the six LGAs constitute a biological onchocerciasis transmission zone (BOTZ)? Kaduna State is an administrative entity that has no impact on interrupting *O.volvulus* transmission. Administrative borders do not restrict the vector.

**Introduction**

**89**	This paper did not consult published papers from Cameroon, Central Africa Republic, and Uganda, which showed that a single annual dose of ivermectin during the same period failed to eliminate onchocerciasis. Is this a recrudescence or premature evaluation of the program before transmission interruption?

Two foci in Kaduna state were named but not shown as delineated. Are their borders adequately delineated, and where is their map based on an appropriate scale, or could it be considered one large undefined biological transmission zone?

**94-109 V**ector migration, or/and the movement of people due to conflict in situations where there is no vector control, can result in recrudescence. A map based on an appropriate scale that shows where the vectors are coming from or people moving from an onchocercaisis endemic area to an area where the disease had been eliminated.

2) **Methods**

**117-138 Study Area.** The major challenge is that the definition of the study area and the adjacent areas/states does not show where delineated biological transmission zones exist. The fact that neighbouring states are under treatment does not necessarily imply that they are related or not to Kaduna's biological transmission zone(s). It seems that not all rivers (big and small) have been prospected to determine whether they support breeding onchocerciasis vectors. This issue complicates the hypothesis that disease recrudescence occurred. It may have been the existence of a few or several undetermined low-level transmission hotspots that were missed during the study that resulted in the declaration of transmission interruption. Good maps are needed to demonstrate the interruption of transmission or that recrudescence occurred.

**139-162	Epidemiological evaluation:** The selection of six out of 23 LGAs shouldn't have been an issue since there were positive kids, but it may be old exposure acquired before the declaration of transmission interruption just before 2022. The absence of delineated biological transmission zones makes it difficult to determine whether the serosurveys were located in appropriate geographic areas.

**3. Results**

**203-237:** The entomological data clearly show that transmission is continuing. About 48.3% of the samples from 3107 children were analyzed, and only 2 kids were positive, leaving us with little to make sense of what is going on in Kaduna.

It could have helped to show some results from the previous published paper on interruption of transmission versus the recent one. I think some valuable maps were lacking.

4) **Discussion:**

Onchocerciasis is a vector-borne focal disease, and delineating biological transmission zones is key to interrupting transmission and testing whether recrudescence occurred. The results did not indicate that recrudescence occurred, and could have been failure of a single annual dose of ivermectin.

5) **a-238-245** I think that there is no need to repeat history in this paragraph.

**b-246- 335.** It seems the rest of the discussion does not apply in the current paper as recrudescence hasn't been proven. Whether this information represented the entire Kaduna State or at least one biological transmission zone of Kaduna State was not determined. It is true that population movement and vector migration can cause onchocerciasis recrudescence, but as a focal disease, the definition of a biological transmission zone is very important.

**c-** In the present circumstances, the paper could be presented as "failure of a single annual dose of ivermectin to interrupt Transmission in Kaduna State in 29 years (1998-2018)" as earlier mentioned in the introduction.
