# Supplementary material for: Recrudescence of transmission of onchocerciasis in some endemic communities in Kaduna State, Nigeria: What is the way forward?
Source: PLoS Negl Trop Dis. 2025 Aug 7;19(8):e0012495. doi: 10.1371/journal.pntd.0012495 (PMC12331076; doi:10.1371/journal.pntd.0012495)
Supplement: Attachment — Submitted filename: Osei-Atweneboana reviewers Comment_15032025.docx [file pntd.0012495.s011.docx]

**Reviewer’s name : Professor Mike Yaw Osei-Atweneboana**

Manuscript Number: PNTD-D-24-01243R1

**Manuscript title:** Recrudescence of transmission of onchocerciasis in some endemic communities in Kaduna State, Nigeria: What is the way forward?

The objective of the study is clearly defined, articulated and appropriate for the study, with clear testable hypothesis stated.

The study area and population were well described, the study design is appropriate to address the stated objectives. The sample size was sufficient to ensure adequate statistical power to address the objectives, however, due to financial challenges encountered by the authors, as a result not all samples collected were analyzed, and this has been indicated as a limitation of the study.

Consent and assent forms were signed for the collection of dried blood spots,

The authors have improved the statistical analysis to enhance the reporting of the results and discussion.

The tables and figures have been revised by the authors

Authors have clearly state the limitations of the study.

This is an important study because it has demonstrated ongoing transmission in some communities in Kaduna State, where interruption of onchocerciasis transmission had been previously established in 2018. This poses a big threat to the onchocerciasis elimination targeted for 2030.

General Comments

This is a comprehensive study, with the goal of determining recrudescence of onchocerciasis transmission in Kaduna State of Nigeria. The study employed entomological, serological and molecular biology techniques to determine blackfly infectivity in pooled blackfly heads using qPCR and collection of dried blood spot from human population to carry out OV16 AP-ELISA to assess seroprevalence in children aged 5-9 years in six of 23 local government areas (LGAs) in Kaduna State.

This is an important study because it has demonstrated ongoing transmission in Kaduna State, where interruption of onchocerciasis transmission had been previously established in 2018. This poses a big threat to the onchocerciasis elimination targeted in 2030.


Following the revision of the manuscript by the authors, the manuscript has improved, however, some sections require minor revisions before it can be accepted for publication.

**The following corrections should be made to the manuscript:**

Ln 28: The authors should indicate why the use of Diethylcarbamazine for human treatment was discontinued.

Ln 206: The statement, “the highest number 2,858 (71.48%) of black flies captured was from Kuratam community” is incorrect as should be revised.

Moreover, the total number of blackflies collected were 6,897, so the percentage of blackflies captured in Kuratam community cannot be 71.48% as indicated ; “2,858 (71.48%)”

Ln 308, the statement “returned when the situation is calm” should read “returned when the situation was calm”

The author has provided the supplement data on the qPCR analysis. However, the qPCR analysis showed significant numbers of *O.ochengi* infections in the blackfly population, but, the authors did not mention the presence of *O. Ochengi* in the study areas.

The authors should explain if the presence of *O. Ochengi* is important in the transmission dynamics of *O. volvulus*, and how this can influence the elimination of onchocerciasis in the study areas.
