# Supplementary material for: Recrudescence of transmission of onchocerciasis in some endemic communities in Kaduna State, Nigeria: What is the way forward?
Source: PLoS Negl Trop Dis. 2025 Aug 7;19(8):e0012495. doi: 10.1371/journal.pntd.0012495 (PMC12331076; doi:10.1371/journal.pntd.0012495)
Supplement: Attachment — Submitted filename: Responses to Reviewers.docx [file pntd.0012495.s013.docx]

**NIGERIAN INSTITUTE FOR TRYPANOSOMIASIS (*AND ONCHOCERCIASIS*) RESEARCH**

No. 1 Surame Road, UngwanRimi GRA, Kaduna, Kaduna State, Nigeria

23^rd^April, 2025

Dear Editor,

PLoS NTD

**Responses to Reviewers’ Comments: PNTD-D-24-01243R1**

Reference to the reviewers’ comments on our manuscript with reference PNTD-D-24-01243R1 the authors responses are as follow:

**Manuscript Number: PNTD-D-24-01243R1**

| **Reviewers’ Comments** | **Authors’ responses** |
| --- | --- |
| **Reviewer Number 1** |  |
| **Line34: Methodology/Principal Findings**  It is not clear why six out of 23 LGAs in Kaduna State were selected for evaluation. Do the six LGAs constitute a biological onchocerciasis transmission zone (BOTZ)?Kaduna State is an administrative entity that has no impact on interrupting *O.volvulus* transmission.Administrative borders do not restrict the vector. | The six LGAs constituting the study areas were purposively selected due to their ecological, socioeconomic indices and the presence of onchocerciasis vector. Five LGAs out of the six LGAs have either river Kaduna or river Gurara transverse them and they are known for breeding black flies. All the LGAs are also known to be insecure due to banditry and kidnapping, communal clashes, farmers-herders clashes and insurgency as earlier stated in the manuscript (Lines 99-104).  It is imperative to state that Federal Ministry of Health (FMoH)/Nigeria Onchocerciasis Elimination Committee (NOEC) classified Kaduna State as onchocerciasis transmission zone in Nigeria (FMoH, 2017) and therefore, the scope of our study was within Kaduna State transmission zone. |
| **Introduction**  **Lines 89:** This paper did not consult published papers from Cameroon, Central Africa Republic, and Uganda, which showed that a single annual dose of ivermectin during the same period failed to eliminate onchocerciasis. Is this a recrudescence or premature evaluation of the program before transmission interruption?  Two foci in Kaduna state were named but not shown as delineated. Are their borders adequately delineated, and where is their map based on an appropriate scale, or could it be considered one large undefined biological transmission zone?  **Line 94 – 109:** Vector migration, or/and the movement of people due to conflict in situations where there is no vector control, can result in recrudescence. A map based on an appropriate scale that shows where the vectors are coming from or people moving from an onchocerciasis endemic area to an area where the disease had been eliminated.  **Methods:**  **Line 117-138 Study Area.** The major challenge is that the definition of the study area and the adjacent areas/states does not show where delineated biological transmission zones exist. The fact that neighbouring states are under treatment does not necessarily imply that they are related or not to Kaduna's biological transmission zone(s). It seems that not all rivers (big and small) have been prospected to determine whether they support breeding onchocerciasis vectors. This issue complicates the hypothesis that disease recrudescence occurred.It may have been the existence of a few or several undetermined low-level transmission hotspots that were missed during the study that resulted in the declaration of transmission interruption. Good maps are needed to demonstrate theinterruption of transmission or that recrudescence occurred.  **139-162 Epidemiological evaluation:** The selection of six out of 23 LGAs shouldn't have been an issue since there were positive kids, but it may be old exposure acquired before the declaration of transmission interruption just before 2022. The absence of delineated biological transmission zones makes it difficult to determine whether the sero surveys were located in appropriate geographic areas  **Results**:  **203-237:** The entomological data clearly show that transmission is continuing. About 48.3% of the samples from 3107 children were analyzed, and only 2 kids were positive, leaving us with little to make sense of what is going on in Kaduna. It could have helped to show some results from the previous published paper on interruption of transmission versus the recent one. I think some valuable maps were lacking.  **Discussion:**  **246- 335:** It seems the rest of the discussion does not apply in the current paper as recrudescence hasn't been proven. Whether this information represented the entire Kaduna State or at least one biological transmission zone of Kaduna State was not determined. It is true that population movement and vector migration can cause onchocerciasis recrudescence, but as a focal disease, the definition of a biological transmission zone is very important.  **Conclusion**:  In the present circumstances, the paper could be presented as "failure of a single annual dose of ivermectin to interrupt Transmission in Kaduna State in 29 years (1998-2018)"as earlier mentioned in the introduction. | It is important to note that FMoH/NOEC in Nigeria declared Kaduna State to have interrupted transmission of onchocerciasis due to prolong use of ivermectin in line with the WHO (2016) and FMoH (2017) elimination guidelines for epidemiological and entomological stop-MDA assessments. The outcome was published by Isiyaku et al. (2022). Meanwhile, Tekle et al. (2012) had earlier reported the potential of Kaduna becoming first State to eliminate onchocerciasis through consistent annual mass administration of ivermectin in the operational areas of the African Programme for Onchocerciasis Control (APOC). Based on the above documentary evidences, Kaduna met stop-MDA thresholds and was declared to have interrupted onchocerciasis. Any other episodes that occur after interruption is possible recrudescence in line with known procedure in epidemiology.  As earlier stated above, FMoH classified a state as transmission zone in Nigeria and it is considered as such for Kaduna in this study.  Kaduna State has a minimum of 117,880 internally displaced persons (IDPs) as a result of banditry and kidnapping, communal clashes, farmers-herders clashes and insurgency and natural disaster.Also, it is important to state that 38,900 IDPs (33%) of the total IDPs identified in Kaduna by International Organization for Migration in year 2023 round 12 assessment migrated to Kaduna State from other neighbouring States.  As stated in our manuscript (Lines 275 – 277), *S. damnosum* can move more than 400km away in large numbers in search of favourable conditions. This study did not investigate vector population movement from one transmission zone to another in Nigeria but possible transmission of Onchocerciasis in some parts of Kaduna State where the level of insecurity is high with history of abundance of black flies (Lines 82-85; 99 -104). The authors have recommended further studies on vector migration, human movement and social-cultural issues in relation to elimination of Onchocerciasis in Kaduna State (Lines 341 – 344).  As earlier mentioned, Kaduna State is a transmission zone (FMoH, 2017) and it was considered as such in this study.  The selected river system and the communities have been documented as breeding sites of *S. damnosum* in Kaduna State. Two of the communities (Kuratam and Gurara) were part of the communities where Isiyaku et al. (2022) carried out epidemiological and entomological assessments for stop-MDA in 2018. Their investigations revealed that neither vector nor humans was infected with *O. volvulus* in the areas. However, four years after we found *O. volvulus* both in vector and humans in the same areas. This result clearly revealed that recrudescence of transmission of onchocerciasis has occurred in the areas.  As earlier mentioned, Kaduna State is a transmission zone (FMoH, 2017) and it was considered as such in this study.  We humbly differ on the suggested title by the reviewer. As explained above, interruption of transmission of onchocerciasis in Kaduna has been established by Isiyaku et al. (2022). Any infection in Kaduna after that is considered recrudescence of transmission of onchocerciasis. Therefore, we stay by the title proposed by our team.  “**Recrudescence of transmission of Onchocerciasis in some endemic communities in Kaduna State, Nigeria: What is the way forward?**”. |
| **Reviewer Number 2** |  |
| (1) Please reconcile information on lines 127 -128 with that on lines 133 – 136. Provide a better map with higher resolution (2) **Lines 176 – 178**: What if you have more than one community within this distance; say one 8km and the other 17km? (3) Line 249 – 250: Is it breeding sites that are washed away? Please clarify (4) Check Lines 338 – 340 (5) Check line 384 | Lines 127 -128 explained the purposive selection of the six LGAs that comprise the study areas while lines 133 – 136 that five out of the six LGAs had *S. damnosum* breeding sites.  Sample collection points within three LGAs (Kaduna North, Kaduna South and Chikun) were closed, increasing the resolution scale of the present map will make the map blurred  We include all the communities within the distance. For instance, Kuratam and Atara are less than 20km apart so both communities were considered first-line communities (Table 1).  The statement has been revised accordingly in the manuscript. “….rainfall and stormy weather play major role in washing away most breeding sites of black flies during the rainy season”.  The statement has been revised accordingly in the manuscript. “Further studies would determine whether the recrudescence observed in this study is driven by vector migration, human migration, socio-cultural issues or insecurity.”  The reference has been revised accordingly in the manuscript. “…of Cross River State, Nigeria after two decadesof mass drug administration with Ivermectin”. |
| **Reviewer Number 3** |  |
| **Line 28**: The authors should indicate why the use of diethylcarbamazine for human treatment was discontinued.  **Line 206**: The statement, “the highest number 2,858 (71.48%) of black flies captured was from Kuratam community” is incorrect as should be revised. Moreover, the total number of black flies collected were 6,897, so the percentage of black flies captured in Kuratam community cannot be 71.48% as indicated ; “2,858 (71.48%)”  **Line 308**: The statement “returned when the situation is calm” should read “returned when the situation was calm”  The author has provided the supplement data on the qPCR analysis. However, the qPCR analysis showed significant numbers of *O. ochengi*infections in the black fly population, but, the authors did not mention the presence of *O. Ochengi* in the study areas. The authors should explain if the presence of *O. Ochengi* is important in the transmission dynamics of *O. volvulus*, and how this can influence the elimination of onchocerciasis in the study areas. | Reason for discontinuation of diethylcarbamazine for human treatment has been indicated within the Abstract. “…due to toxicity”.  The percentage has been presented accurately in the revised manuscript. “The highest number 2,858 (41.44%) of black flies captured was from Kuratam community…”  The statement has been revised accordingly in the manuscript. “… returned when the situation was calm”.  Though *Simulium* complex can transmit both *O. volvulus* and *O. Ochengi* in Onchocerciasis endemic areas, humans cannot serve as host for *O. Ochengi* but can only host *O. volvulus*. Therefore, our focus is human onchocerciasis and we have presented our data as such. |

Yours sincerely,

**Timothy O. OLANREWAJU**

Corresponding Author

[timothyseye@gmail.com](mailto:timothyseye@gmail.com)
