# Supplementary material for: Recrudescence of transmission of onchocerciasis in some endemic communities in Kaduna State, Nigeria: What is the way forward?
Source: PLoS Negl Trop Dis. 2025 Aug 7;19(8):e0012495. doi: 10.1371/journal.pntd.0012495 (PMC12331076; doi:10.1371/journal.pntd.0012495)
Supplement: Attachment — Submitted filename: Responses to the Editor Comments.docx [file pntd.0012495.s014.docx]

**NIGERIAN INSTITUTE FOR TRYPANOSOMIASIS (*AND ONCHOCERCIASIS*) RESEARCH**

No. 1 Surame Road, UngwanRimi GRA, Kaduna, Kaduna State, Nigeria

17^th^ June, 2025

Dear Editor,

PLoS NTD

**Responses to the Editor’s Comments: PNTD-D-24-01243R2**

Reference to the editor’s comments on our manuscript with reference PNTD-D-24-01243R2 the authors responses are as follow:

**Manuscript Number: PNTD-D-24-01243R2**

| **Comments** | **Authors’ responses** |
| --- | --- |
|  |  |
| The authors did not include a version which accurately showed track changes. This makes it hard for reviewers to review properly. Please make sure documents are correct when submitting.  I believe it is PLOS NTD formatting rules to have the Methods before the Results section. Please check the style guide. The Limitations and Conclusions should stay after the Discussion.  The map is improved and useful, however, it is still small and hard to see. If the authors are concerned with the map blurring, consider 2 separate maps, one showing Chikun, and Kadunga North and South, and one showing Kachia and Kagarko. It was unclear where Zangon-Kataf was. I could not find it on the map.  As the rivers are referred to often in the manuscript, they should be labeled on the map. It would also help to have the symbols show which sites had positive DBS samples.  The authors make the key assertion that recrudescence has taken place in this area. However, one alternative explanation is that transmission was never actually interrupted in the first place. The authors cite the Tekle et al. and Isiyaku et al. papers as evidence of interruption, but don’t really interrogate the results in those papers. It would be very relevant to the community to use the Discussion section to compare the authors’ results (and the geographic areas studied) with the results in the earlier papers. Which data from those papers make the authors think that there was a definitive demonstration of interruption of transmission? Could some foci have been missed? Just repeating that Kaduna is a transmission zone is not enough; were there focal areas within Kaduna shown to have been interrupted? Were those areas near where this study took place? This discussion should help these authors make the case that this is recrudescence. | The revised sections are highlighted in yellow in “Most latest revised manuscript with track changes” attached document.  Methods section as be placed before the Results (Lines 117 – 216)  Revised maps have been inserted accordingly  Tekle et al. (2012) and Isiyaku et al. findings have been discussed and how the geographical areas of studies are related to our study (Lines 280 – 301)  Earlier studies by Crosskey (1956) identified five transmission foci along the rivers in Kaduna State (Zaria, Gurara, Kaduna Central, Birnin Gwari and Kauru/Lere). The present study covered only two transmission foci (Gurara and Kaduna Central) out of five transmission foci in the Kaduna State. Tekel et al. (2012) evaluated impact of continuous treatment with ivermectin on transmission of onchocerciasis in two transmission foci of Birnin Gwari and Kauru/Lere and reported zero prevalence of *O. volvulus* in 3,703 skin-snipped individuals from 27 communities evaluated after 15 – 17 years continuous treatment with ivermectin. Furthermore, Isiayaku et al. (2022) carried out epidemiological and entomological assessments of onchocerciasis for stop-MAM assessment in all the five transmission foci in Kaduna State within 2016 and 2018 after 20 years of consistent treatment with ivermectin in accordance to WHO 2016 onchocerciasis elimination guidelines. While Tekel et al. (2012) alluded to the possibility of elimination of onchocerciasis in Kaduna State after consistent treatment with ivermectin, Isiayaku et al. (2022) on the other hand categorically documented attainment of interruption of transmission of onchocerciasis in Kaduna State after prolong administration of ivermectin.  After five years post interruption of transmission of onchocerciasis in Kaduna State, we investigated seven black flies breeding sites of which two (Gurara with Latitude 9.665326; Longitude 7.883456 and Kuratam with Latitude 9.600633; Longitude 7.816031) were among the sites earlier investigated by Isiayaku et al. (2022). We obtained 25% and 24% *O. volvulus* infectivity rate in black flies captured at Gurara and Kuratam breeding sites respectively as against zero prevalence reported by Isiayaku et al. (2022). Therefore, the high *O. volvulus* infectivity rate in black flies observed in this study indicated recrudescence of transmission of onchocerciasis in the study areas. |
| Abstract (line 42)  0.32 should have a percentage (“) after it, correct? This same number does not have a percentage in the Results section either.  To non-Oncho specific readers it may be unclear as to the relevance of the UCL .371 in line 43. Consider adding both LCL and UCL, or explaining.  Line 56 suggest saying “…Kaduna State four years after a stop MAM decision.”    Line 74-77 can you provide citations for this sentence?    Line 77, after the word “include” remove the semicolon.    Line 78, impacts instead of impact.    Line 90 define MAM the first time it is used    Line 90 space after the number 17    Table 1 is missing the prevalence number to go with the LCL and UCL.  Line 268: define DBS first time it is used.    Line 319 flies instead of fly    Line 329 space after the number 7 | The figure 0.32 has been written with percentage (0.32%) in the Abstract section and Results section (Line 42; 235)  UCL has been written in full in the Abstract section and Results section (Line 43; 219; 234)  Line 56 has been revised according “….Kaduna State four years after a stop-MAM decision.”  Citation has been included “Ubachukwu, 2006” (Line 77; 429)  Line 77 has been revised according. “…. include…”  Line 78 has been revised according. “…. impacts…”  MAM has been written in full as “…mass administration of medicine…” before abbreviation (Line 90)  Line 90 has been revised according “…17 years…”  The prevalence number (0.13) has been inserted in Table 1  Dried blood samples (DBS) has been mention first in the Method section. (Line 162)  The word “fly” has been revised according “…flies…” (Line 209)  Line 359 has been revised according “…5-7 years…” |

Yours sincerely,

**Timothy O. OLANREWAJU**

Corresponding Author

[timothyseye@gmail.com](mailto:timothyseye@gmail.com)
